# Searching for Suitable [Cu(N^N){(PPh_2_)_2_C_2_B_9_H_10_}] Thermally Activated Delayed Fluorescent Dopants: Optimization of the Quantum Yield through the 2‑(4-Thiazolyl)benzimidazole Diimine Functionalization

**DOI:** 10.1021/acs.inorgchem.5c05030

**Published:** 2026-05-12

**Authors:** Irati Barriendos, Olga Crespo, M. Concepción Gimeno

**Affiliations:** Departamento de Química Inorgánica, 16765 Instituto de Síntesis Química y Catálisis Homogénea (ISQCH). Universidad de Zaragoza-CSIC, Zaragoza E-50009, Spain

## Abstract

The quantum yield of thermally stable, neutral [Cu­(dppnc)­(R-tbz)] complexes, where dppnc = {(PPh_2_)_2_C_2_B_9_H_10_}^−^, exhibiting thermally activated delayed fluorescence (TADF), has been optimized to values as high as 42% through strategic variation of the substituent on the thiazolyl unit of the Htbz (2-(4-thiazolyl)­benzimidazole) ligand. A comprehensive analysis of structural features, geometric distortion, steric hindrance, and Δ*E*(S_1_-T_1_) and *E*(T_1_) reveals that maximizing quantum yield requires the razionalization of other nonradiative routes, in addition to those affecting TADF. This study provides key insights for the rational design of highly emissive copper-based complexes. In contrast, the use of the analogous neutral diphosphane [dppcc = (PPh_2_)_2_C_2_B_10_H_10_], forming the corresponding cationic [Cu­(dppcc)­(R-tbz)]^+^ complexes, allows modulation of the emission energy but results in significantly lower quantum yields.

## Graphical abstract

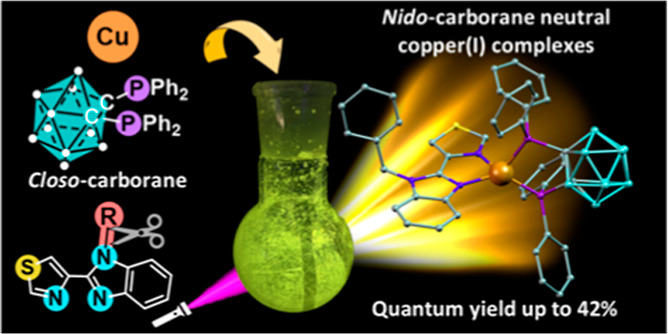

## Introduction

Heteroleptic copper luminescent complexes of the formula [Cu­(N^N)­(P^P)]^*n*^ (*n* = 0, +1) have emerged as a relevant group of emissive materials in response to the search for less expensive and environmentally friendly alternatives to heavy-metal emissive species. The nature of the observed emissions at room temperature in most of these heteroleptic copper systems has been classified as thermally activated delayed fluorescence (TADF). Through theoretical studies, the origin of these emissions has been primarily assigned to metal–ligand (diphosphane, L) to ligand (diimine, L′) charge transfer transitions (MLL’CT) mixed, in some cases, with intraligand (diimine) transitions (IL’).^1^

TADF has emerged as a promising alternative to phosphorescence in boosting the internal quantum efficiency of light-emitting devices such as OLEDs and LECs by enabling 100% of the generated excitons, bypassing the requirement for a heavy atom.^2^ Therefore, in the initial phase, the emphasis of the study of these copper TADF emitters was guided by their capacity for designing ecofriendly and cheaper lighting devices. However, they’ve also shown their skill both as catalysts and sensitizers in photocatalysis.^3–10^

A comprehensive investigation into the potential of these compounds and the modulation of their luminescent properties has been carried out, highlighting their structural tunability and the role of both ligands.^11–14^ These studies have been reviewed in different publications, underscoring that not many neutral diimine/diphosphane heteroleptic copper­(I) complexes have been reported.^1,2,13,15–21^ Given that solubility, aggregation suitability, and the absence of noninnocent anions in the media can be important factors to improve the potential of these complexes, our interest has been directed toward the design of neutral heteroleptic diimine/diphosphane copper­(I) complexes. With this objective, we have selected the anionic *nido*-carborane diphosphane [(PPh_2_)_2_C_2_B_9_H_10_]^−^ (dppnc^–^), which exhibits electronic delocalization via the carborane cluster and acts as an electron-withdrawing ligand, promoting the synthesis of gold emissive complexes, offering an advantage over the analogous cationic derivatives with the neutral [(PPh_2_)_2_C_2_B_10_H_10_] (dppcc) diphosphane.^22^

Only a few copper [Cu­(N^N)­(P^P)] complexes incorporating the dppnc^–^ diphosphane have been reported, mostly containing phenanthroline-type diimines.^9,16,23^ In this context, we have demonstrated the impact of the presence of the analogous anionic dppnc^–^ or neutral dppcc diphosphanes in the emissive properties of the cationic [Cu­(dppcc)­(Htbz)]­PF_6_ (*closo-* **I**) and neutral [Cu­(dppnc)­(Htbz)] (*nido-* **II**) complexes (Figure 1).^24^ TADF has been found for the neutral complex *nido-* **II**, with Φ = 16% in the solid state, whereas the cationic complex (*closo-* **I**) does not exhibit TADF, but its emission is very sensitive to temperature changes and to the media, although it displays a significantly lower quantum yield (1%).

**Figure 1:**
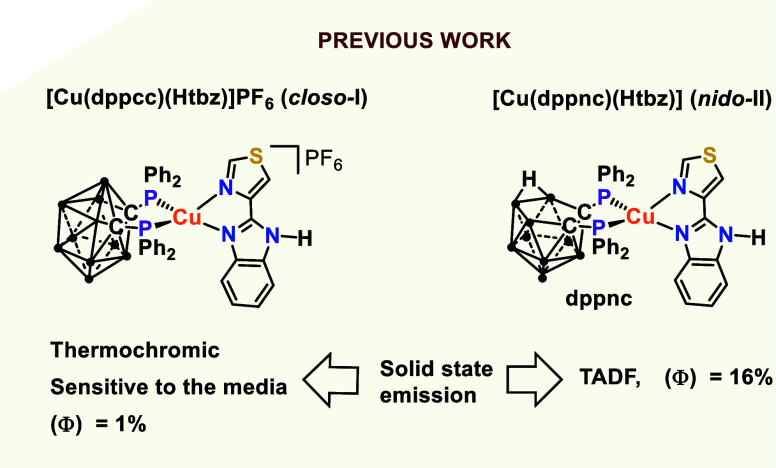
*Closo* **-(I)** and *nido* **-(II)** complexes reported with the unsubstituted 2-(4-thiazolyl)­benzimidazole (Htbz) ligand.

Given the promising emissive properties (TADF and acceptable quantum yield) of [Cu­(dppnc)­(Htbz)], we focused on enhancing these properties. Previous studies have shown that the emission quantum yield of the cationic compound [Cu­(Dpephos)­(Htbz)]­PF_6_ increases upon substitution of the NH hydrogen atom at the Htbz ligand with different R substituents. Remarkably, some [Cu­(Dpephos)­(R-tbz)]­PF_6_ have been used in device development.^25,26^

This finding has directed our focus toward designing a series of R-tbz ligands (Figure 2) and investigating how the functionalization of the Htbz ligand affects the emissive properties of neutral *nido-*carborane complexes [Cu­(dppnc)­(R-tbz)], with specific attention to the influence of different parameters on the quantum yield. The thermochromic response and media sensitivity of *closo*-species [Cu­(dppcc)­(R-tbz)]­PF_6_, compared with [Cu­(dppcc)­(Htbz)]­PF_6_ (*closo-* **I**), have also been studied. We have further assessed the outcome of replacing the thiazolyl moiety in the N^N ligand by an oxazolyl group to further understand the impact of heterocycle identity on the photophysical properties. Our objective also includes contributing to a comprehensive data set that correlates steric and excited-state parameters with Φ, thereby facilitating the rational design of future complexes.

**Figure 2:**
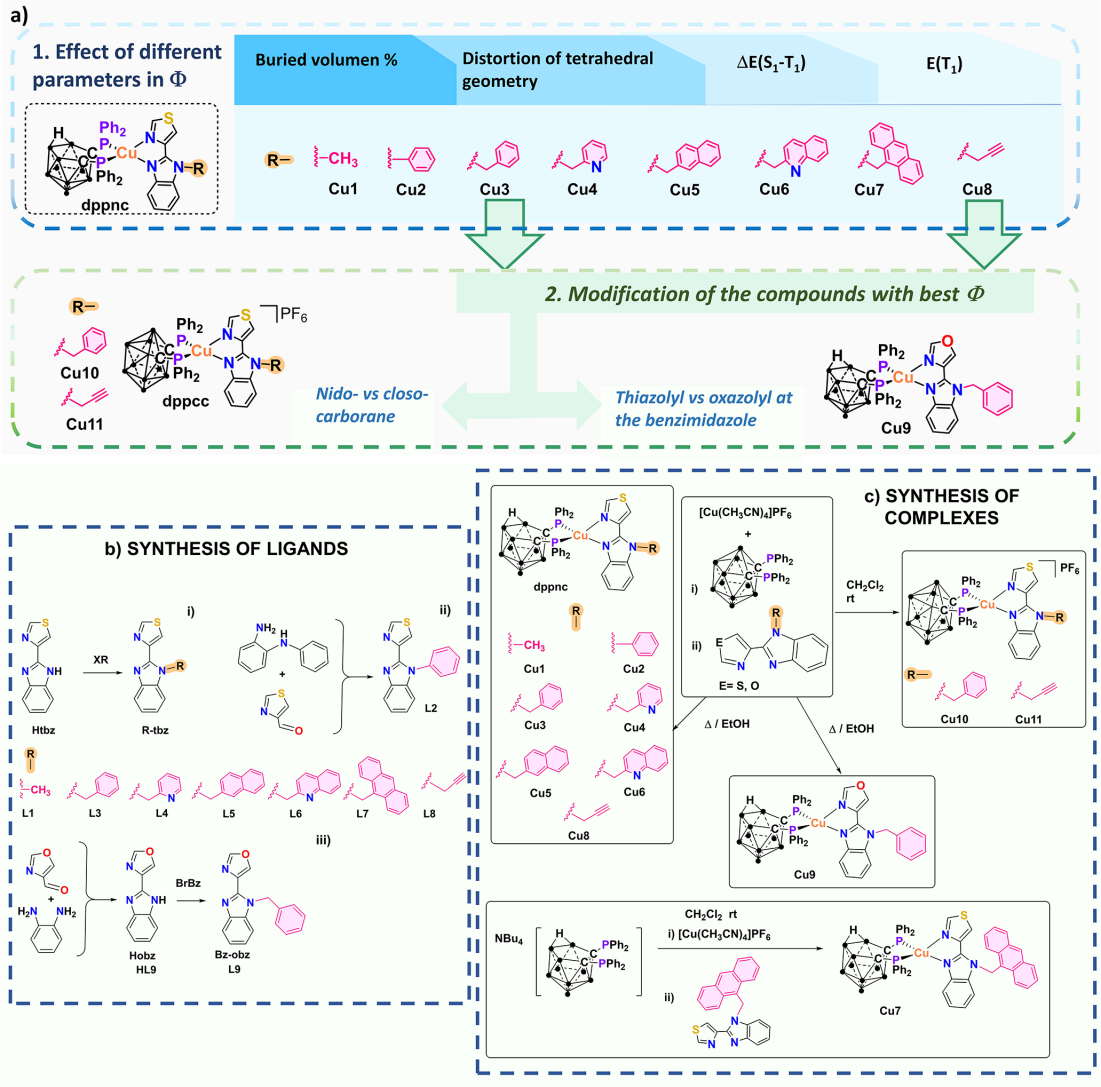
(a) Design strategy followed in this study. (b) Synthesis of ligands. (c) Synthesis of complexes.

## Discussion

### Design Strategy, Synthesis, and Structural Considerations

The substituents used for the preparation of ligands **L1**–**L9** (Figure 2) have been selected in order to evaluate the donor/acceptor nature and the extent of the aromaticity. Once the new ligands were prepared, the strategy resumed in Figure 2 was followed. In the initial step, the *nido* complexes were synthesized, and their emissive properties were studied with special attention to the analysis of steric, geometric, electronic, and energetic parameter effects on the quantum yield. To complete the study, we have also selected those R-tbz ligands leading to the best quantum yield and compared the emissive properties of the analogous *nido-* and *closo*-complexes. In addition, as the Bz-tbz ligand leads to the *nido*-compound with the highest quantum yield, we explored the impact of the heteroatom by replacing the 4-thiazolyl unit with the 4-oxazolyl moiety, and compound [Cu­(dppnc)­(Bz-obz)] was prepared to assess the influence of this substitution on the photophysical properties.

The R-tbz and Bz-obz ligands (Figures 2 and S1) have been afforded from the reaction of the XR precursor with 2-(4-thiazolyl)­benzimidazole in basic media or via the Debus–Radziszewski coupling of the corresponding diimine and aldehyde. The synthesis of the heteroleptic complexes [Cu­(dppnc)­(R-tbz)] (Figures 2 and S2) was achieved through a two-step procedure to avoid the formation of the bis­(diimine)­copper complexes. The first step is the reaction of [Cu­(NCCH_3_)_4_]­PF_6_ with diphosphane, followed by the addition of diimine in the second step. In the formation of the *nido*-complexes, the nucleophilic attack, leading to the *nido*-diphosphane and the neutral copper complexes, is provided by the use of ethanol as a solvent (Scheme S2). Compound **Cu7** is obtained from the tetrabutylammonium salt of the *nido*-diphosphane.

The presence of a broad signal at approximately −2 ppm proves the partial degradation of the carborane cluster and the presence of *nido*-diphosphane in the neutral complexes. This signal corresponds to the bridging hydrogen atom on the carborane open face. Due to the complicated pattern of the ^1^H NMR spectra, not all the hydrogen atoms can be assigned (Figures S3–S60).

In addition to NMR spectroscopy and mass spectrometry characterization (Figures S61–S76), single-crystal X-ray diffraction studies of the *nido-*complexes **Cu2**, **Cu3**, **Cu5**, **Cu6**, **Cu8**, and **Cu9** and the *closo*-derivative **Cu11** have been carried out (Figures S122–S129 and Table S1). Refinement of the crystal structure of **Cu9** leads to a high residual factor (R_1_) and weighted residual factor (WR_2_). The residual electron density, which could not be fitted to a model, was treated with Squeeze, but it still remains electron density near the copper atom and one of the phosphorus atoms. The checkCIF corresponding to the study of **Cu9** highlights these problems, and the crystal data (which confirm the connectivity) have not been deposited in the CCDC, but the ESI contains the molecular diagram and unit cell details. It is outstanding that in complexes **Cu5** and **Cu6** with 2-naphtylmethyl and 2-quinolinylmethyl, the molecules exhibit dimeric aggregation via π···π stacking interactions involving imidazole rings of adjacent molecules (Figure 3).

**Figure 3:**
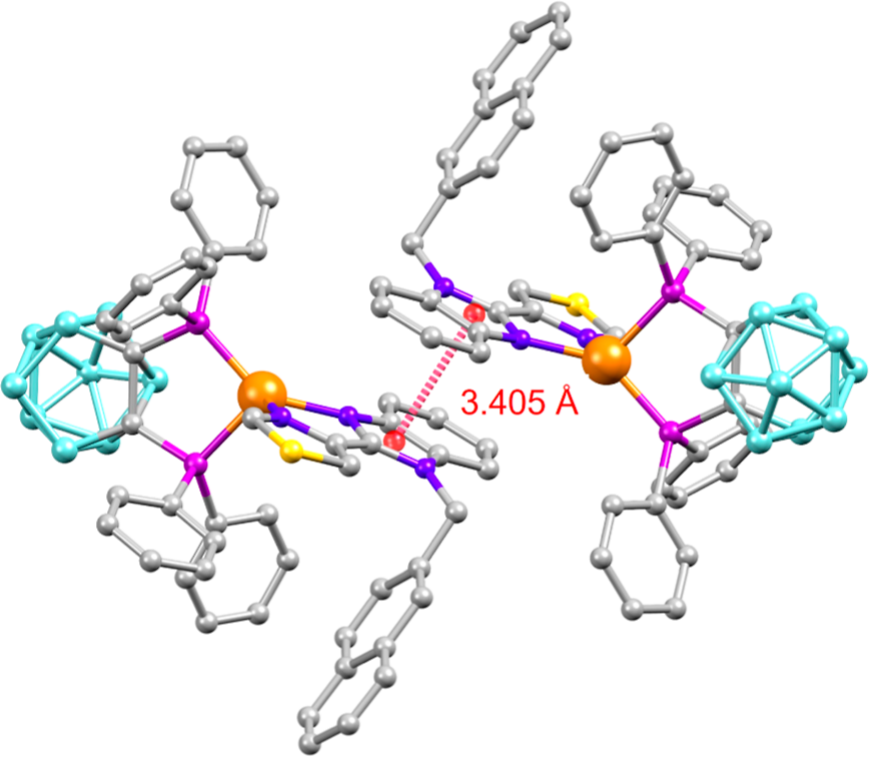
Molecular diagram of compound **Cu5** displaying the π···π interactions involving imidazole rings of different molecules.

The distortion from the ideal tetrahedral environment displayed by the copper atom in the complexes may be related to the angle (α) between the planes defined by the two phosphorus atoms and the copper center and the plane formed by the two nitrogen atoms and the copper center, which should be 90° for the ideal tetrahedral geometry. The observed values for the complexes are listed in Table 1.

**Table 1:** Calculated α Angle from the X-ray Data for Different Complexes^a^

compound	Cu2	Cu3^a^	Cu4	Cu5	Cu6	Cu8^a^
**α**	89.54 89.96	87.90 85.53	89.41	88.83	88.58	82.38

a Complexes which display the highest quantum yields (Tables 2 and 3).

X-ray data enable calculation of the buried volume percentage (%*V*_bur_), which specifies the volume occupied by a ligand or a set of ligands within a spherical region of a defined radius around the metal center^27,28^ and may be related to the steric hindrance around the copper atom. Significant steric hindrance inhibits the transition from a tetrahedral to a square planar geometry around the copper atom. This phenomenon is of relevance not only to ground-state distortions but also to those in the excited state, where severe distortion could facilitate nonradiative relaxation via the square planar geometry.

Table 2 shows the calculated %*V*_bur_ for the diphosphane {%*V*_bur_ (P^P)}, diimine {%*V*_bur_ (N^N)}, and both ligands {(N^N) + (P^P)} together {total %*V*_bur_} from the crystal X-ray studies, and the corresponding topographical steric maps for **Cu4** are shown in Figure 4 (see Figures S114–S121 for the rest of the complexes). The position of the R substituent at the N^N ligand, opposite to the ligand face coordinated to the Cu atom, leads to slight variations in %*V*_bur_ (N^N) values. The highest %*V*_bur_ (N^N) values are found for **Cu4** (*R* = 2-pyridylmethyl), **Cu5** (*R* = 2-naphtylmethyl), and **Cu9**, revealing a small increment for complexes with the benzyl R substituent by substituting thiazolyl (**Cu3**) with the oxazolyl (**Cu9**) group.

**Table 2:** %*V*_bur_ for P^P, N^N, and {(N^N) + (P^P)} Blocks in Complexes **Cu1**–**Cu9**, Emission Quantum Yields [Φ (%)], and First Excited Triplet Energy [*E*(T_1_)]^a^

	%*V*_bur_ ^a^			Φ (%)^b^	*E*(T_1_) (cm^–1^)^c^
	P^P	N^N	{(N^N) + (P^P)}		
**Cu1**				4	20362
**Cu2**	53.4	33.3	84.7	23	20591
	52.9	33.6	86.0		
**Cu3**	52.0	33.1	84.4	42	20032
	52.3	33.2	85.0		
**Cu4**	52.7	33.8	84.1	3	20377
**Cu5**	53.4	34.0	85.1	13	20130
**Cu6**	51.3	33.4	82.9	13	20287
**Cu8**	52.8	33.5	85.0	31	21168
**Cu9**	53.6	33.8	84.5	10	20064

a Calculated from the crystal structure data, see Supporting Information for additional details.

b Obtained for powder samples at room temperature.

c Calculated from the λ_onset_ of the emission spectrum at 77 K. The emission band profile of **Cu7** at 77 K does not allow accurate calculation.

**Figure 4:**
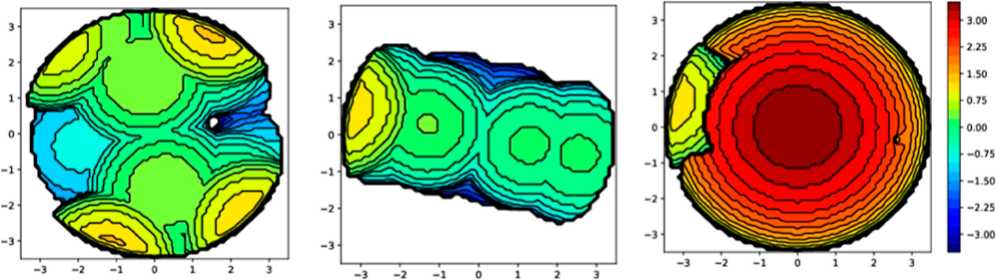
From left to right, the image shows the topographical steric maps in **Cu4** for dppnc, L4, and {(dppnc) + L4}.

**Table 3:** Emissive Properties of Complexes **Cu1**–**Cu11** in the Solid State^a^

compound		λex^a^	λem^a^	Φ (%)	*K*_r_ (ms^–1^)	*K*_nr_ (ms^–1^)	τ^b^ (ms)
**Cu1**	rt	420	554	4	6.4 × 10^3^	1.5 × 10^5^	0.256
	77 K	440	555				3.634
**Cu2**	rt	400	535	23	1.5 × 10^2^	5.0 × 10^2^	0.035
	77 K	400	555				2.606^d^
**Cu3**	rt	420	530	42	2.6 × 10^2^	1.5 × 10^2^	0.108
	80 K	400	545				2.387*^d^
**Cu4**	rt	360	532	3	8.7 × 10^2^	2.8 × 10^4^	0.026
	77 K	360	548				1.941
**Cu5**	rt	420	560	13	1.8 × 10^3^	1.2 × 10^4^	0.231
	77 K	420	565				2.240
**Cu6**	rt	420	533	13	1.8 × 10^2^	1.6 × 10^3^	0.023
	77 K	390	554				3.046
**Cu7**	rt	400	540	<1	-	-	low int
	77 K	-	-	-	-	-	-
**Cu8**	rt	420	520	31	2.1 × 10^2^	4.7 × 10^2^	0.066
	77 K	400	535				2.439^d^
**Cu9**	rt	415	532	10	2.9 × 10^2^	2.6 × 10^3^	0.029
	77 K	425	570				1.524
**Cu10**	rt	370	698	1^c^	-	-	0.067
	77 K	385	500		-	-	
		365	698				0.572
**Cu11**	rt	370	698	2^c^	-	-	0.094
	77 K	384	486		-	-	
		367	700				1.660

a Emission (λem) and excitation (λex) maxima.

b Fitted to a monoexponential curve marked with*; the rest were fitted to a double exponential function (see Supporting Information).

c Not possible to separate both components.

d Data at 80 K.

Greater differences than those observed in %*V*_bur_ (N^N) are found in the %*V*_bur_ (P^P) values, with the lowest values found for **Cu3** and **Cu6**. The lowest total %*V*_bur_ value is found for **Cu6**.

The analysis of complexes with two molecules in the asymmetric unit shows that for **Cu3**, the total %*V*_bur_ and those corresponding to the ligands are very similar for both molecules, but for compound **Cu2**, total %*V*_bur_ and P^P %*V*_bur_ are quite different for the two molecules, which could point to the influence of packing effects in addition to the steric effect in isolated molecules.^29^ In this sense, the π···π interactions observed in **Cu5** and **Cu6** are shorter in **Cu6**, which could lead to a slight separation of the diimine ligand from the copper center and a possible explanation of the smaller %*V*_bur_ (N^N) and total %*V*_bur_ observed in **Cu6**, compared with those in **Cu5.**

### Photophysical Properties

In the solid state, both at room temperature and at 77 K, the complexes exhibit yellow (*nido*-) or red (*closo*-) emissions, perceivable to the naked eye with excitation maxima wavelength ranges of 360–420 nm at rt and 360–440 nm at 77 K [Figure 5a, Table 3, Figures S77–S87 (emission and excitation spectra), and Figures S88–S107 (lifetime decay curves)].

**Figure 5:**
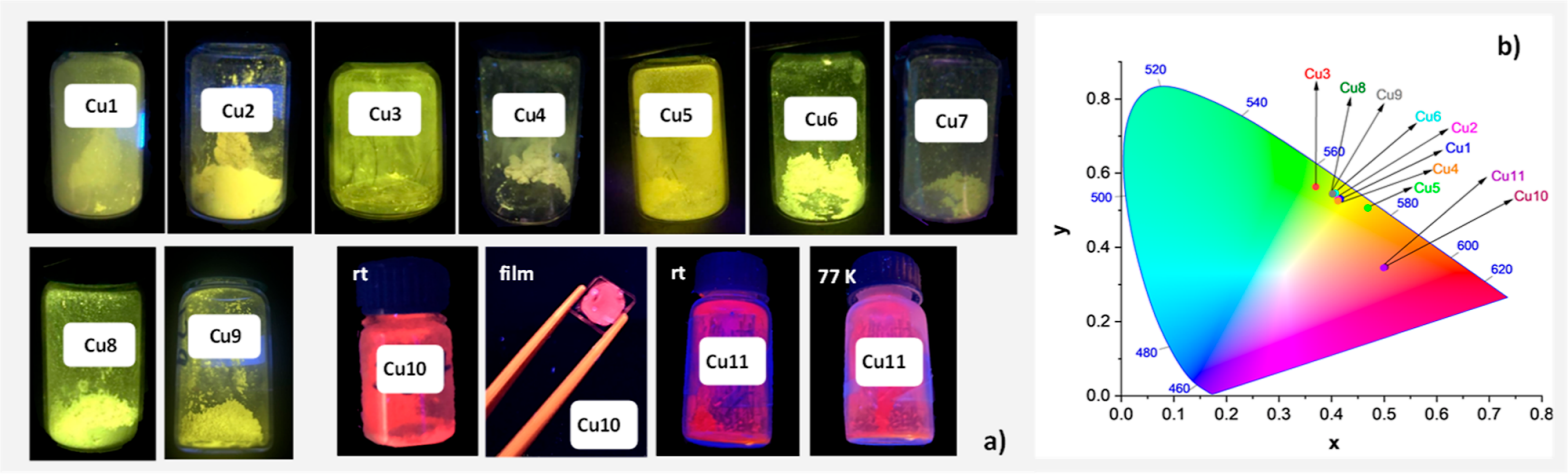
(a) Solid samples of complexes **Cu1**–**Cu11** under UV light (λ = 350 nm). (b) *x* and *y* CIE 1931 color coordinates for complexes **Cu1**–**Cu11**.

From the quantum yield measurements, after removal of excitation light, the 1931 CIE color coordinates x and y are obtained (Figure 5b). In heteroleptic [Cu­(N^N)­(P^P)]^+^ complexes, copper and diphosphane orbitals are reported to contribute mainly to the HOMO, to which the N^N ligand does not contribute or contributes to a much lesser extent. These N^N ligand orbitals contribute mainly to the LUMO. Thus, the transitions are described as metal ligand (diphosphane) to ligand (diimine) charge transfer transitions (MLL’CT) mixed with intraligand (N^N) transitions (IL’).

For the complexes presented in this study, the N^N ligand appears to contribute to both the HOMO and the LUMO. This inference is supported by the observation that the emission energy is only slightly affected by variation of the R substituent on the N^N ligand. As a result, the introduction of either donor (-Me) or acceptor (-Ph) substituents does not significantly shift the emission color, likely because both frontier orbitals are influenced simultaneously. If the diimine ligand contributed exclusively to the LUMO, donor substituents would be expected to raise its energy and thus increase the emission energy. This effect is not observed when comparing **Cu1**, bearing a methyl group, with **Cu2**, bearing a phenyl substituent.^30^

The contribution of the diphosphane to the HOMO orbital has been observed for the unsubstituted complexes [Cu­(dppcc)­(Htbz)]­PF_6_ (*closo-* **I**) and [Cu­(dppnc)­(Htbz)] (*nido-* **II**).^24^ The copper atom participates in the HOMO in both complexes; however, in the *closo*-compound, the phosphorus and phenyl rings of the diphosphane contribute to the HOMO, and in the *nido*-compound, it is the *nido*-carborane cage which contributes to the HOMO. As a result, emissions at room temperature shift from yellow for the *closo*-complexes to red for the *nido*-complexes.

In the following discussion, we analyze the results in terms of the *closo-* or *nido-*nature of carborane diphosphane.

### TADF Studies

For the neutral complexes **Cu1**–**Cu9** with the *nido*-diphosphane, the red shift of the emission maxima observed in most of the cases and an important increment of the lifetime upon cooling let us to propose the expected TADF nature of the emissions at room temperature.^12,31^ The complexes with higher quantum yields (**Cu2**, **Cu3**, and **Cu8**) have been selected in order to prove this point. The lifetime of the first excited singlet and triplet states, as well as the energy gap Δ*E*(S_1_-T_1_), have been calculated by measuring the lifetime at different temperatures and fitting the data to eq 1 (K_B_ = Boltzmann constant) using the least-squares fitting method (Figures 6, S108, and S109).

$$\tau =\frac{3+e^{-\Delta E\left(S_{1}-T_{1}\right)/K_{B}T}}{3\left(\frac{1}{\tau \left(T_{1}\right)}\right)+\left(\frac{1}{\tau \left(S_{1}\right)}\right)e^{-\Delta E\left(S_{1}-T_{1}\right)/K_{B}T}}$$ 1

**Figure 6:**
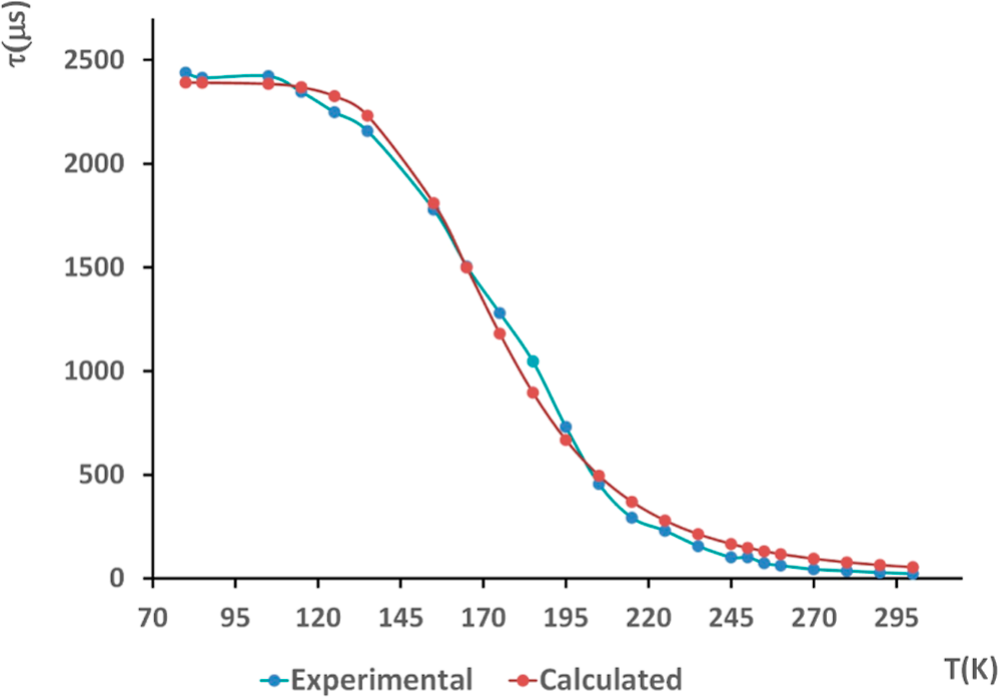
Temperature dependence of the emission lifetime of complex **Cu8** in the solid state and the corresponding fitting values using eq 1 (Chi-Square = 0.996).

The room temperature lifetimes lie in the plateau corresponding to low lifetime values, which is in line with TADF behavior at room temperature.^32^ The Δ*E*(S_1_-T_1_) energy gaps (Table 4) are within the range to consider their suitability as dopants for light devices (around or below 1000 cm^–1^). Prompt fluorescence lifetimes are 1 or 2 orders of magnitude shorter than TADF. The first excited triplet energy has been estimated from the onset value of the emission spectra (Tables 2 and 4).

**Table 4:** Lifetimes of the Delayed Fluorescence (DF) and the S_1_ and T_1_ Excited States (μs). Energy Gap Δ*E*(S_1_-T_1_) (cm^–1^) and *E*(T_1_) (cm^–1^) Energy for a Selection of [Cu­(dppnc)­(R-tbz)] Complexes^a^

compound	τ_DF_(S_1_)	τ(S_1_)^a^	τ(T_1_)^a^	Δ*E*(S_1_-T_1_)^a^	*E*(T_1_)^b^
**Cu2**	35	0.122	2610	1170	20591
**Cu3**	108	0.524	1834	896	20032
**Cu8**	66	0.096	2390	1094	21168
*nido-* **II** ^**[24]**^				1097	

a From fitting lifetime data at different temperatures to eq 1, K_B_ = Boltzmann constant.

b Calculated from the onset of the emission spectrum at 77 K.^33^.

### Quantum Yields

As explained in the Introduction section, substitution of the NH proton in [Cu­(Dpephos)­(Htbz)]­PF_6_ leads to higher quantum yields. It has been claimed that the substitution avoids hydrogen interactions, which have been claimed to quench the emission. We have studied a wide variety of substituents, and substitution of the NH hydrogen atom may lead either to an increase or a decrease in quantum yield (Figure 7), compared with that of *nido-* **II** (16%). The higher quantum yields were observed for R = benzyl (**Cu3**, 42%), propargyl (**Cu8**, 31%), and phenyl (**Cu2**, 23%); other substituents reduce the quantum yield with the lowest found for *R* = 9-anthrylmethyl (**Cu7**).

**Figure 7:**
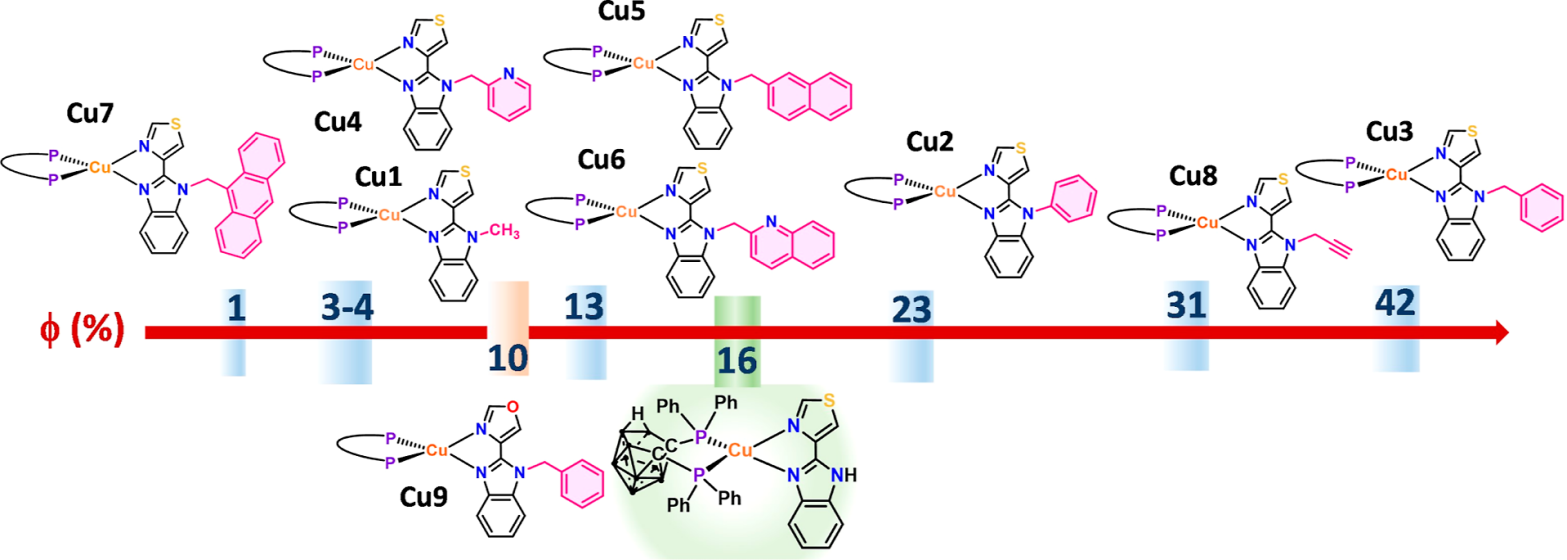
Quantum yields of complexes [Cu­(dppnc)­(R-tbz)], compared with the non-substituted compound [Cu­(dppnc)­(Htbz)] (*nido-* **II**).

The high quantum yield and Δ*E*(S_1_-T_1_) energy gaps of complexes **Cu2**, **Cu3**, and **Cu8** reveal promising potential for their implementation in optical devices. Consequently, their thermal stability has been studied, and the corresponding curves are provided in Figures S111–S113. The three complexes are stable up to 300 °C. The total weight loss does not exceed 30% (**Cu3**) or 17% (**Cu2**, **Cu8**) and proceeds in two steps. The thermal stability (up to about 300 °C) is comparable to that reported for complexes such as [Cu­(Dpephos)­(Htbz)]^+^, [Cu­(Dpephos)­(R-tbz)]^+^ (R = different substituents), or [Cu­(P^P)­(tbz)] (P^P = Dpephos, Xantphos).^25,26,36^

Figure 7 reveals that it is difficult to find a trend relating electronic properties of the R fragment incorporated to the N^N ligand and the resulting changes in the quantum yield compared with the unsubstituted compound *nido-* **II**. The introduction of the donor fragment Me (**Cu1**) diminishes Φ, while the acceptor group Ph (**Cu2**) leads to an increase in the quantum yield. The effect of different groups bonded to the nitrogen through a methylene group is very different. For R = benzyl (**Cu3**), the quantum yield increases; for R = PyCH_2_ (**Cu4**), it decreases; and for *R* = 2-quinolinylmethyl (**Cu6**) and 2-naphtylmethyl (**Cu5**), it decreases and almost leads to the same quantum yields.

The lowest quantum yield observed for *R* = 9-anthrylmethyl (**Cu7**) may be associated with the reported well-documented tendency of anthracene and its derivatives to undergo aggregation, leading to aggregation-induced quenching (AIQ).^34,35^ An increase of the aromaticity of the group bonded to the methylene unit [phenyl (**Cu3**) < naphthyl (**Cu5**) < anthryl (**Cu7**)] leads to a progressive decrease of the quantum yield, which may be understood in terms of the aggregation-induced quenching (AIQ), favored by the presence of π···π interactions, as commented on above.^34,35^ Extension of aromaticity also favors vibrational deactivation pathways.

The presence of the nitrogen atom in substituents PyCH_2_ (**Cu4**, Φ = 3%) and 2-quinolynyl-methyl (**Cu6**, Φ = 13%) affects the aromaticity of the ring and introduces *n* → π* transitions which have been reported to reduce quantum yield.^37^ This diminishment is observed when **Cu3** is compared with **Cu4**, but not when **Cu5** is compared with **Cu6.**

As the benzyl substituent leads to the highest quantum yield, it has been selected for the heteroatom modification, and the resulting compound **Cu9**, featuring the oxazolyl group at the N^N core, exhibits a lower quantum yield than **Cu3**.

These data demonstrate that different deactivation pathways may compete and must have been taken into account, in addition to those related to the deactivation of the TADF mechanism [low energy of the T_1_ state^28^ (see below) or distortion from the square planar geometry]. All of the mechanisms together will contribute to the final quantum yield of the TADF emission.

### Energies of Excited States Affecting TADF

Both the Δ*E*(S_1_-T_1_) energy gap and *E*(T_1_) energy have been reported to affect the quantum yield for TADF emitters. A small Δ*E*(S_1_-T_1_) energy gap is essential in order to favor RISC. In addition, low *E*(T_1_) energies would enhance deactivation of the excited state, leading to lower quantum yields.^28^ Thus, from the onset of the emission spectra at 77 K, we calculated the *E*(T_1_) energies for complexes **Cu1**– **Cu9**. Table 2 shows that values do not follow the expected trend, as similar *E*(T_1_) energies may lead to very different quantum yields. If we do not take into account complexes for which the presence of *n* → π* transitions or AIQ would compete with TADF and focus on **Cu2**, **Cu3**, and **Cu8**, which also show the higher quantum yields, we can observe that the quantum yield increases when Δ*E*(S_1_-T_1_) decreases, but increasing *E*(T_1_) does not lead to higher quantum yields. Thus, small Δ*E*(S_1_-T_1_) values may compensate for low *E*(T_1_) values, leading to higher K_RISC_ constants and the highest quantum yield in **Cu3** (Figure 8).

**Figure 8:**
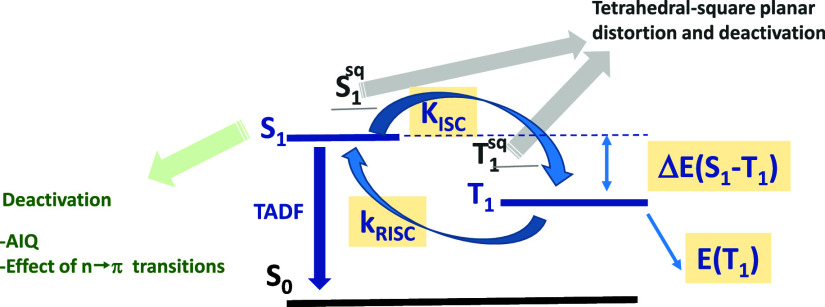
Different deactivation pathways and parameters governing quantum yield.

Taking into account that TADF behavior, instead of mixed phosphorescent-TADF emission, is observed at room temperature (see above), moderate spin–orbit coupling (SOC) is expected, as phosphorescence has been reported to be favored over TADF for strong SOC.^38^

### Steric and Geometric Factors

Distortion from the Tetrahedral Geometry and steric Factors (%V_bur_). Distortion from tetrahedral to square planar geometry has been described as a deactivation route.^4^ A relationship between higher distortion from the ideal tetrahedral geometry around copper in the ground state (smaller α angle from the X-ray structure) and lower Φ is not inferred from Table 1, as among the complexes studied, complexes **Cu3** and **Cu8** stand out for their higher distortion (smaller α angles) and higher quantum yields.

This fact does not preclude a distortion in the excited state, leading to square planar deformation suitable for non-radiative deactivation. In this sense, we have studied %*V*_Bur_ (Table 2). It must be highlighted that these data correspond to the ground state, but they may provide an indication about the steric hindrance toward the copper atom and the resistance to deformation from tetrahedral to square planar geometry. In fact, for different series of complexes, a correlation between %*V*_Bur_ and Φ has been found.^27,28^ Overall (see above), our studies reveal the key role of the packing effects in %*V*_bur_.

The lowest %*V*_bur_ has been found for **Cu6,** which does not show the lowest quantum yield. As explained above, for **Cu6**, other deactivation routes in addition to that related to tetrahedral geometry deformation should be taken into account. Thus, a more realistic analysis consists of the comparison among **Cu2**, **Cu3**, and **Cu8**, for which no AIQ and *n* → π* transitions are expected to compete with TADF. Similar %*V*_bur_ values have been found for **Cu2**, **Cu3**, and **Cu8**, which fits with the proposal that geometry distortion toward the copper center does not appear to be the determining factor for the differences in quantum yields, which instead seem to be governed by equilibrium between Δ*E*(S_1_-T_1_) and *E*(T_1_).

A resume of the different deactivation pathways is given in Figure 8.

### Closo-Derivatives

The R substituents that led to *nido*-complexes with the highest quantum yields (benzyl and propargyl) were selected to synthesize the analogous *closo*-derivatives **Cu10** and **Cu11**, which are red emissive at room temperature (Table 3, Figures 5 and 9). The aim was to investigate three key aspects: (i) whether *closo*-derivatives display lower Φ than the analogous *nido*-compounds, as previously reported for other three-coordinated group 11 complexes,^22^ (ii) whether the medium sensitivity observed for the [Cu­(dppcc)­(Htbz)]­PF_6_ (*closo-* **I**) is maintained upon substitution of the hydrogen at the NH unit, and (iii) whether the TADF emissive behavior is exclusive of *nido*-derivatives.

**Figure 9:**
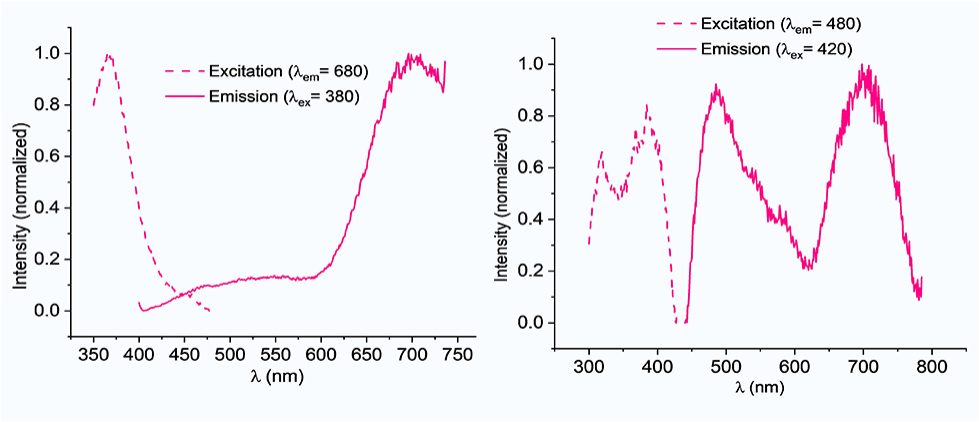
Illustration of the dual emission dependence on the excitation wavelength of **Cu11** at 77 K.

As expected, we have confirmed that the *closo-* **I**, **Cu10**, and **Cu11** complexes show lower quantum yields than the analogous *nido*-derivatives *nido-* **II**, **Cu3**, and **Cu8** (Table 3). The additional negative charge, mostly focused in the carborane cage, in the *nido*-derivatives may favor the MLL’CT transitions which originate the emissions and lead to a general increase of quantum yield for *nido*-derivatives compared with analogous *closo*-derivatives.

The pronounced thermochromism of compound **I** results in an emission shift from red at room temperature to yellow at 77 K, a phenomenon not observed in **Cu10** and **Cu11,** which exhibit a more intricate behavior. At room temperature, they are red emitters, as *closo-* **I**, but at 77 K, both exhibit dual emission. In addition to the red emission, a new emission appears in the yellow region. For both complexes, the intensity of the band in the yellow region increases upon excitation from 370 to 420 nm, and a higher intensity for the band in the red region is observed upon excitation at lower wavelengths (Figure 9). The low intensity of the yellow component precluded measurement of its lifetime (Table 3).

## Conclusions

The photoluminescence quantum yield of neutral [Cu­(dppnc)­(R-tbz)] complexes exhibiting thermally activated delayed fluorescence (TADF) has been optimized up to 42% through substitution with the thiazolyl–benzimidazole ligand. The results indicate that the nature of substituents replacing the NH proton modulates the quantum yield. AIQ and the n→π transitions compete with TADF, lowering the quantum yields. For R = Ph, CH_2_Ph, and CH_2_–C≡CH, these mechanisms do not act, and the studies reveal that small Δ*E*(S_1_-T_1_) may compensate for low *E*(T_1_) energies. In addition, similar %*V*_bur_ values have been found for the three complexes. Thus, geometry distortion toward the copper center does not appear to be the determining factor for the differences in quantum yields.

The photophysical properties of the most emissive neutral *nido*-carborane diphosphane complexes (R = propargyl, benzyl) were compared to those of their cationic *closo*-analogues [Cu­(dppcc)­(R-tbz)]­PF_6_. Two points are noteworthy: (i) substitution at the NH position of the benzimidazole ring in the *closo-*derivatives did not significantly improve quantum yields in the *closo*-derivatives studied, and (ii) the data point to an essential role of the *nido*-carborane diphosphane in enabling efficient TADF.

This study contributes to the relatively small but growing family of neutral [Cu­(N^N)­(P^P)] TADF emitters, some of which demonstrate excellent quantum yields and promising potential for optoelectronic applications.

Furthermore, the steric, geometric, and electronic parameters evaluated here can contribute to a valuable data set to inform structure–property relationships, guiding the rational design of next-generation copper­(I)-based luminescent materials.

## Experimental Section

### Instrumentation

A Bruker AV 400 or 300 was used for NMR spectra measurements. Chemical shifts (ppm) reported are relative to the solvent peaks of the deuterated solvent.^39^ High-resolution mass spectra-ESI (HRMS-ESI) were carried out in a Bruker MicroTOF-Q spectrometer equipped with an API-ESI source and a QTOF mass analyzer, both allowing a maximum error in the measurement of 5 ppm. The thermogravimetric analyses were carried out in TA Instruments SDT2960 equipment at a rate of 10 °C min^–1^ under a nitrogen atmosphere until 600 °C and under an air atmosphere from 600 to 750 °C.

Steady-state photoluminescence spectra and lifetimes were recorded in a FluoTime300 PicoQuant spectrometer. Films were placed in an adjustable front-face holder, and solid powders were placed within a quartz tube and placed inside a quartz dewar. Lifetime studies at different temperatures were carried out in an Optistat DN Oxford variable temperature liquid nitrogen cryostat under Ar; the rest of the measurements were carried out under air.

Quantum yields were measured by the absolute method, under air, using a Hamamatsu Quantaurus-QY C11347 compact one-box absolute quantum yield measurement system. The reproducibility of the measurements was confirmed for solids by comparing three or more measurements performed with different solid amounts. The relative uncertainty for the absolute method has been determined as less than 6% through studies carried out for different substances using both the absolute method and the comparative one.^40^

### Crystallography

The crystals suitable for X-ray studies obtained by diffusion were mounted on a MiTeGen Crystal micromount and transferred to the cold gas stream of a Bruker D8 VENTURE (2) diffractometer. Data were collected using monochromated MoΚα radiation (λ = 0.71073 Å). Scan type is ω. Absorption correction based on multiple scans was applied with the program SADABS.^41^ The structures were refined on F^2^ using the program SHELXL-2018.^42^ All non-hydrogen atoms were refined anisotropically. Hydrogen atoms were included using a riding model. CCDC depositions 2446989 (**Cu2**), 2446993 (**Cu3**), 2446984 (**Cu4**), 2446978 (**Cu5**), 2446982 (**Cu6**), 2446986 (**Cu8**), and 2446991 (**Cu11**) contain the supplementary crystallographic data. These data can be obtained free of charge from The Cambridge Crystallography Data Center.

### Calculation of the Buried Volume

The SambVca 2.1 package^43^ (https://www.molnac.unisa.it/OMtools/sambvca2.1/idex.html) was used to calculate buried volumes^44^ and topographical steric maps.^45^ The center of the sphere was defined by the copper atom, and the selected parameters were as follows: Bondi radii: 1.17; sphere radius *r* = 3.5 Å; mesh spacing: 0.10, and the H atoms are excluded. The following selection of the axis and atoms were used for each %*V*_bur_: diphosphane [%*V*_bur_(P^P)]: the P atoms of the diphosphane were selected to define the negative *Z*-axis, and the C and N atoms of the carbene ligand coordinated to the copper center of the carbene ligand to define the *XZ*-plane. All atoms except those of the diphosphane ligand were deleted; diimine [%*V*_bur_(N^N)]: the N atoms of the diimine ligand coordinated to the copper center were used to define the negative *Z*-axis and the P atoms of the diphosphane to define the *XZ*-plane. All the atoms except those of the diimine ligand were deleted; diphosphane + diimine: [%*V*_bur_{P^P) + (N^N)}]: the N atoms of the diimine ligand coordinated to the copper center were used to define the negative *Z*-axis and the P atoms of the diphosphane to define the *XZ*-plane. The copper atom was deleted.

### Synthesis and Characterization

All experiments and manipulations were carried out using degassed solvents and under an argon atmosphere. All starting materials are commercially available and were used as received without further purification. The diphosphanes 1,2-(PPh_2_)_2_-1,2-C_2_B_10_H_10_ and [NBu_4_]­[7,8-(PPh_2_)_2_-7,8-C_2_B_9_H_10_] were prepared using a published procedure.^46,47^ Further details are included in the Supporting Information.

## Supplementary Material



## Notes

Experimental details, NMR spectra, mass spectra, X-ray crystal bond distances and angles, TGA curves, photophysical studies (emission, excitation spectra, lifetime fitting curves, 1931 CIE color coordinates, and temperature dependence of the emission lifetime), and buried volume percentages (PDF)
