# Supplementary material for: Searching for Suitable [Cu(N^N){(PPh2)2C2B9H10}] Thermally Activated Delayed Fluorescent Dopants: Optimization of the Quantum Yield through the 2‑(4-Thiazolyl)benzimidazole Diimine Functionalization
Source: Inorg Chem. 2026 May 12;65(20):10904–13. doi: 10.1021/acs.inorgchem.5c05030 (PMC13213895; doi:10.1021/acs.inorgchem.5c05030)
Supplement: Supplementary file 1 [file ic5c05030_si_001.pdf]

Supporting information

Searching for suitable $[Cu(N^\wedge N)\{(PPh_2)_2C_2B_9H_{10}\}]$ thermally activated delayed fluorescent dopants. Optimization of the quantum yield through the 2-(4-thiazolyl)benzimidazole diimine functionalization

Irati Barriendos,[a] Olga Crespo*[a] and Mª Concepción Gimeno*[a]

[a] Departamento de Química Inorgánica, Instituto de Síntesis Química y Catálisis Homogénea (ISQCH). Universidad de Zaragoza-CSIC. E-50009 Zaragoza, Spain.

*Corresponding author E-mail: ocrespo@unizar.es (O. C.), gimeno@unizar.es (M. C. G.)

S1.- Experimental section 2

S2.- NMR spectra 10

S3.- Mass Spectra 39

S4.- Emission and excitation spectra 43

S5.- Lifetime fitting curves 47

S6.- TADF fitting 57

S7.- CIE 1931 x, y coordinates 58

S8.- Thermal gravimetric analysis (TGA) curves 58

S9.- % Buried volumes 60

S10.- Crystal X-ray data: Diagrams, bond distances (Å) and angles (°) 62

S1.- Experimental section

S1. Synthesis and characterization

All experiments and manipulations were carried out using degassed solvents and under argon atmosphere. All starting materials are commercially available and were used as received without further purification. The diphosphanes 1,2-$(PPh_2)_2$-1,2-$C_2B_{10}H_{10}$ and $[NBu_4][7,8-(PPh_2)_2-7,8-C_2B_9H_{10}]$ were prepared using a published procedure.[1,2]

S1.1 Preparation and characterization of the ligands L1-L9

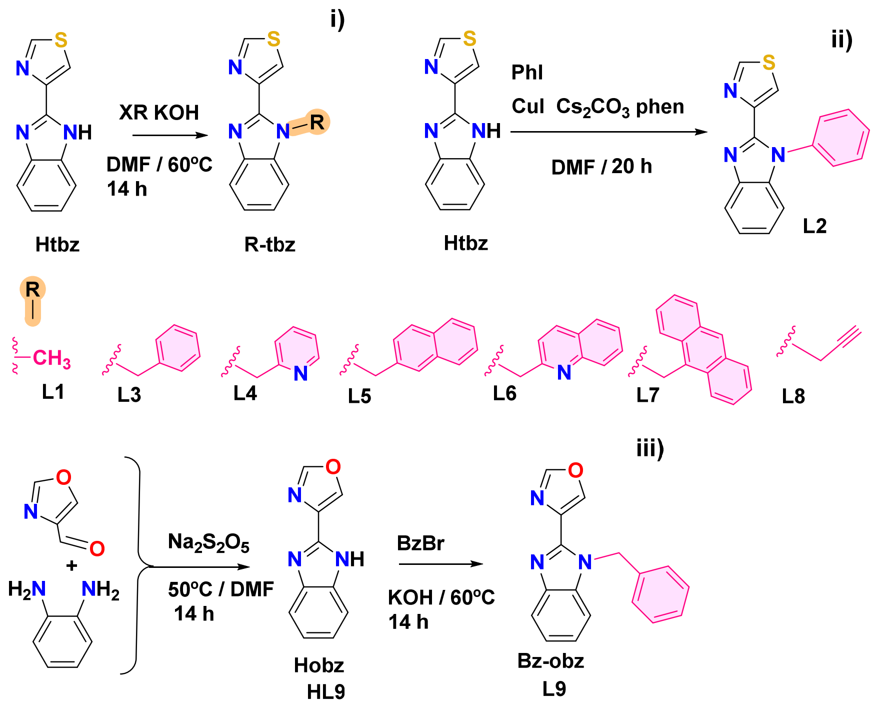

Figure S1. General procedure for the synthesis of the ligands. **L1-L9**, *i)* **L1, L3-L8**; *ii)* **L2**; *iii)* **L9**.

Synthesis of L1, L3, L4, L5, L6, L7, L8 and L9

L8 was prepared using a formerly reported method.[3] **L1**, **L3**, **L6** were prepared according to previously published methods too but with some modifications.[4,5] A mixture of 2-(4-Thiazolyl)benzimidazole (2 mmol, 402.5 mg) or 2-(4-Oxazolyl)-1*H*-benzimidazole (2 mmol, 364.5 mg), potassium hydroxide (5 mmol, 280.5 mg) and the corresponding alkyl halide (2.1 mmol; iodomethane: 134 µL, benzyl bromide: 250 µL, 2-(Bromomethyl)pyridine: 531.2 mg, 2-(Bromomethyl)naphthalene: 464.3 mg, 2-(Bromomethyl)quinoline: 449.6 mg, 9-(Chloromethyl)anthracene: 476.1 mg) were dissolved in DMF (10 mL). The reaction mixture was stirred at 60 °C for 14 hours. After

cooled to rt, the mixture was diluted in water (20 mL) and extracted with dichloromethane (3 x 10 mL). The combined organic phases were dried over $MgSO_4$, filtered, and the solvent was evaporated under reduced pressure. Addition of n-hexane (10 mL) led to the precipitation of the corresponding ligand as a white solid.

L1 (R = methyl)

374.6 mg. Yield: 87 %. The spectroscopic data agree with the values provided in the literature.

^{1}H NMR (300.1 MHz, $CDCl_3$, rt, ppm) δ = 8.94 (dd, J = 2.2, 1.0 Hz, 1H), 8.31 (dd, J = 2.2, 1.0 Hz, 1H), 7.86 – 7.74 (m, 1H), 7.48 – 7.28 (m, 3H), 4.24 (s, 3H).

L3 (R = benzyl)

520.5 mg. Yield: 89 %. The spectroscopic data agree with the values provided in the literature.

^{1}H NMR (300.1 MHz, $CDCl_3$, rt, ppm) δ = 8.88 (d, J = 2.2 Hz, 1H), 8.32 (d, J = 2.2 Hz, 1H), 7.87 – 7.78 (m, 1H), 7.37 – 7.10 (m, 8H), 6.08 (s, 2H).

L4 (R = 2-pyridilmethyl)

397.4 mg. Yield: 68 %. HRMS (ESI-QTOF) m/z**:** $[M+Na]^+$ Calculated for $C_{16}H_{12}N_4NaS$ 315.0675; Found 315.0667.

^{1}H NMR (300.1 MHz, DMSO-d_6, rt, ppm) δ = 9.26 (d, J = 2.1 Hz, 1H), 8.55 (d, J = 2.1 Hz, 1H), 8.43 (ddd, J = 4.8, 1.8, 1.0 Hz, 1H), 7.77 – 7.61 (m, 2H), 7.57 – 7.42 (m, 1H), 7.31 – 7.16 (m, 3H), 7.03 (dt, J = 7.9, 1.0 Hz, 1H), 6.19 (s, 2H). $^{13}C\{^1H\}$ APT (75.5 MHz, DMSO-d_6, rt, ppm) δ = 156.6 (s, 1C), 155.2 (*, 1C), 149.2 (s, 1C), 147.0 (s, 1C), 146.7 (s, 1C), 142.5 (s, 1C), 137.0 (s, 1C), 135.9 (s,1C), 122.9 (s, 1C), 122.6 (s, 1C), 122.5 (s, 1C), 122.4 (s, 1C), 120.9 (s, 1C), 119.1 (s, 1C), 110.0 (s, 1C), 49.5 (s, 1C).

* = Signal determined by HSQC (^1H-$^{13}C\{^1H\}$) experiment.

L5 (R = 2-naphthylmethyl)

570.2 mg. Yield: 83 %. HRMS (ESI-QTOF) m/z: $[M+Na]^+$ Calculated for $C_{21}H_{15}N_3NaS$ 364.0879; Found 364.0879.

^{1}H NMR (300.1 MHz, DMSO-d_6, rt, ppm) δ = 9.32 (d, J = 2.1 Hz, 1H), 8.58 (d, J = 2.1 Hz, 1H), 7.89 – 7.67 (m, 4H), 7.63 (s, 1H), 7.59 – 7.52 (m, 1H), 7.45 (dt, J = 6.3, 3.4 Hz, 2H), 7.37 – 7.16 (m, 3H), 6.26 (s, 2H). $^{13}C\{^1H\}$ APT (75.5 MHz, DMSO-d_6, rt, ppm) δ = 155.5 (*, 1C), 146.9 (s, 1C), 146.6 (s, 1C), 142.6 (s, 1C), 135.7 (s, 1C), 135.1 (s, 1C), 132.7 (s, 1C), 132.2 (s, 1C), 128.3 (s,1C), 127.6 (s, 1C), 127.5 (s, 1C), 126.4 (s, 1C), 126.0 (s, 1C), 125.1 (s, 1C), 124.8 (s, 1C), 122.9 (s, 1C), 122.8 (s, 1C), 122.5 (s, 1C), 119.2(s, 1C), 111.1 (s, 1C), 47.8 (s, 1C).

* = Signal determined by HSQC (^1H-$^{13}C\{^1H\}$) experiment.

L6 (R = 2-quinolylmethyl)

503.5 mg. Yield: 73 %. The spectroscopic data agree with the values provided in the literature.

^{1}H NMR (300.1 MHz, $CDCl_3$, rt, ppm) δ = 8.83 (d, *J* = 2.2 Hz, 1H), 8.41 (d, *J* = 2.2 Hz, 1H), 8.18 – 8.06 (m, 1H), 8.03 – 7.90 (m, 1H), 7.83 (dt, *J* = 8.0, 1.2 Hz, 1H), 7.76 – 7.67 (m, 2H), 7.50 (ddd, *J* = 8.0, 6.9, 1.2 Hz, 1H), 7.44 – 7.35 (m, 1H), 7.33 – 7.27 (m, 1H), 7.26 – 7.14 (m, 1H), 7.03 (d, *J* = 8.5 Hz, 1H), 6.36 (s, 2H).

L7 (R = 9-anthrylmethyl)

446.3 mg. Yield: 57 %. HRMS (ESI-QTOF) m/z: $[M+Na]^+$ Calculated for $C_{25}H_{17}N_3NaS$ 414.1035; Found 414.1028.

^{1}H NMR (300.1 MHz, $CDCl_3$, rt, ppm) δ = 9.02 (d, *J* = 2.2 Hz, 1H), 8.53 (s, 1H), 8.45 (d, *J* = 2.2 Hz, 1H), 8.41 – 8.31 (m, 2H), 8.11 – 8.00 (m, 2H), 7.68 (d, *J* = 8.1 Hz, 1H), 7.52 – 7.38 (m, 4H), 7.10 (s, 2H), 7.03 (t, *J* = 7.7 Hz, 1H), 6.65 (t, *J* = 7.7 Hz, 1H), 6.26 (d, *J* = 8.3 Hz, 1H).

L9 (R = benzyl)

280.1 mg. Yield 51%. HRMS (ESI-QTOF) m/z: $[M+Na]^+$ Calculated for $C_{17}H_{13}N_3NaO$ 298.0951; Found 298.0940

^{1}H NMR (300.1 MHz, DMSO-d_6, rt, ppm) δ = 8.06 (d, *J* = 1.1 Hz, 1H), 7.81 (d, *J* = 1.1 Hz, 1H), 6.85 – 6.59 (m, 3H), 6.52 – 6.26 (m, 6H), 5.16 (s, 2H).

Synthesis of 2-(4-Oxazolyl)-1H-benzimidazole (LH9)

HL9 was prepared using the previously reported procedure for similar ligands with some modifications.[6] A mixture of o-phenylene diamine (2 mmol, 216.3 mg), 4-Oxazolecarboxaldehyde (2 mmol, 194.1 mg) and sodium metabisulfite (4 mmol, 760.5 mg) was dissolved in DMF (10 mL). The reaction mixture was stirred at 50 °C for 10 hours. After cooled to rt, the mixture was diluted in water (20 mL) and extracted with ethyl acetate (3 x 10mL). The combined organic phases were dried over $MgSO_4$, filtered, and the solvent was evaporated under reduced pressure. Addition of n-hexane (10 mL) led to the precipitation of HL9 as a pale brown solid. Yield: 230.5 mg, 62 %.

^{1}H NMR (300.1 MHz, $CDCl_3$, rt, ppm) δ =9.91 (br, 1H), 8.47 (s, 1H), 8.01 (s, 1H), 7.78 (br, 1H), 7.51 (br, 1H), 7.30 (dd, *J* = 6.0, 3.2 Hz, 2H).

Synthesis of L2 (R = phenyl)

L2 was prepared using the previously reported procedure for similar ligands[7] , from 2-(4-Thiazolyl)benzimidazole (5 mmol, 1006.2 mg), phenyl iodide (7 mmol, 784 μL), CuI (1.7

mmol, 323.8 mg), 1,10-phen (3 mmol, 540.6 mg) and Cs_2CO_3 (8 mmol, 325.8 mg). Yield: 645 mg, 45%. HRMS (ESI-QTOF) m/z: $[M+Na]^+$ Calculated for $C_{16}H_{11}N_3$ Na S 300.0566; Found 300.0568.

^{1}H NMR (300.1 MHz, DMSO-d_6, rt, ppm) δ = 9.37 (d, J = 2.1 Hz, 1H), 8.58 (d, J = 2.1 Hz, 1H), 7.89 – 7.67 (m, 4H), 7.63 (br s, 1H), 7.59 – 7.62 (m, 1H), 7.45 (m, 2H), 7.37 – 7.16 (m, 3H), 6.26 (s, 2H).

^{13}C{^{1}H} APT (75.5 MHz, DMSO-d_6, rt, ppm) δ = 154.4 (*, 1C),146.8 (s, 1C), 146.0 (s, 1C), 142.3 (s, 1C), 136.7 (s, 1C), 136.6 (s, 1C), 129.5 (s,1C), 128.5 (s, 1C), 127.4 (s, 1C), 123.6 (s, 1C), 122.9 (s, 1C), 122.6 (s, 1C), 119.4(s, 1C), 110.6 (s, 1C).

* = Signal determined by HSQC (^{1}H-^{13}C{^{1}H}) experiment.

S1.2 Preparation and characterization of complexes Cu1-Cu11

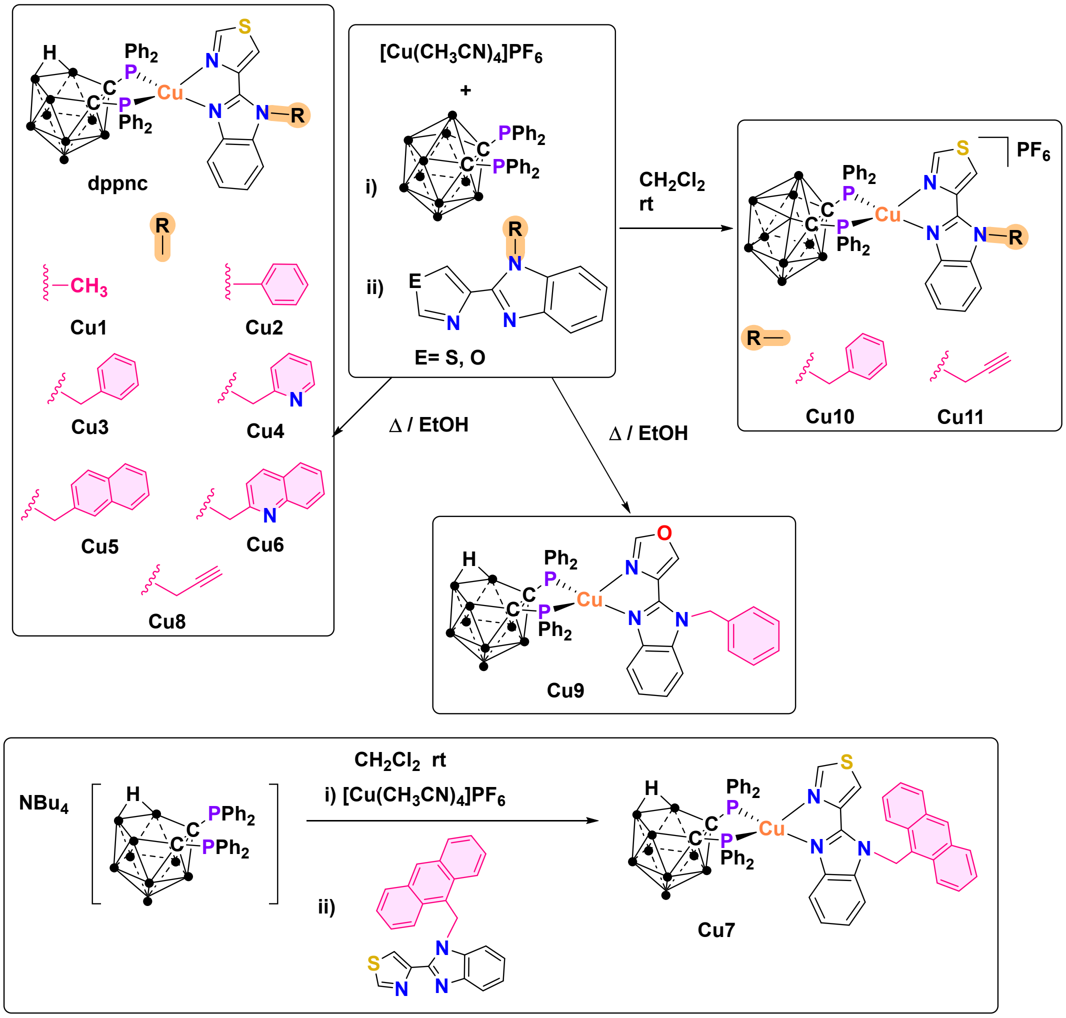

Figure S2. General procedure for the synthesis of the complexes **Cu1-Cu11**

Synthesis of complexes Cu1, Cu2, Cu3, Cu4, Cu5, Cu6, Cu8 and Cu9

To a solution of $[Cu(CH_3CN)_4](PF_6)$ (0.3 mmol, 111.8 mg) in ethanol (10 mL) $[1,2\text{-}(PPh_2)_2\text{-}1,2\text{-}C_2B_{10}H_{10}]$ (0.3 mmol, 153.80 mg) was added. The mixture was stirred for 30 min, and the corresponding substituted derivative of 2-(4-thiazolyl)benzimidazole (0.3 mmol; **L1**: 60.4 mg, **L2**: 83.2 mg, **L3**: 87.4 mg; **L4**: 87.7 mg **L5**: 113.1 mg; **L6**: 113.7 mg; **L8**: 71.7 mg; **L9**: 81.7 mg) was added. The mixture was refluxed for 2 h. The resulting precipitate was filtered, dissolved in dichloromethane (10 mL), and filtered through celite®. The concentration of the solution (5 mL) and addition of n-hexane (10 mL) led to the precipitation of a yellow solid which was dried under vacuum.

Cu1 (R = methyl)

159.2 mg. Yield: 68 %. HRMS (ESI-QTOF) m/z: $[M+H]^+$ Calculated for $C_{37}H_{40}B_9Cu N_3P_2S$ 782. 2546; Found 782.2575.

^{1}H NMR (300.1 MHz, acetone-*d$_6$*, rt, ppm) δ = 8.73 (d, *J* = 1.7 Hz, 1H, H2), 7.81 (d, *J* = 8.2 Hz, 1H), 7.66 – 7.08 (m, 24H), 4.35 (s, 3H, H3), -1.82 (br s, 1H, H-BHB) .^{31}P{^{1}H} NMR (121.5 MHz, acetone-*d$_6$*, rt, ppm) δ = 16.5.

Cu2 (R = phenyl)

174.4 mg. Yield: 68 %. HRMS (ESI-QTOF) m/z: $[M+Na]^+$ Calculated for $C_{42}H_{41}B_9CuN_3NaP_2S$ 866.2528; Found 866.2537.

^{1}H NMR (400.1 MHz, acetone-*d$_6$*, *rt,* ppm) δ = 7.87–6.94 (m, 31H), -1.87 (br s, 1H, H-BHB). ^{31}P{^{1}H} NMR (162.0 MHz, acetone-*d$_6$*, *rt,* ppm) δ = 17.4.

Cu3 (R = benzyl)

180.2 mg. Yield: 70 %. HRMS (ESI-QTOF) m/z: $[M+Na]^+$ Calculated for $C_{43}H_{43}B_9CuN_3NaP_2S$ 880.2679; Found 880.2719.

^{1}H NMR (400.1 MHz, acetone-*d$_6$*, *rt,* ppm) δ = 8.41 (d, *J* = 1.7 Hz, 1H, H2), 7.79 (d, *J* = 8.2 Hz, 1H, H4), 7.64 – 7.11 (m, 29H), 6.05 (s, 2H, H3), -1.86 (br s, 1H, H-BHB). ^{31}P{^{1}H} NMR (162.0 MHz, acetone-d$_6$, rt, ppm) δ = 16.9. ^{13}C{^{1}H} APT (100.6 MHz, acetone-d$_6$, rt, ppm) δ = 147.3 (s, 1C), 143.3 (s, 1C), 140.9 (s, 1C), 137.8 (s, 1C), 137.4 (t, *J* = 15.0 Hz, 2C), 136.6 (t, *J* = 19.0 Hz, 2C), 136.3 (s,1C), 135.8 (t, *J* = 9.8 Hz, 4C), 133.5 (t, *J* = 7.5 Hz, 4C), 130.9 (s, 1C), 130.1 (s, 1C), 129.2 (s, 1C), 129.0 (s, 1C), 128.9 – 128.6 (m, 13C), 127.0 (s, 1C), 126.0 (s, 1C), 125.3 (s, 1C), 121.7 (s, 1C, C2), 119.9 (s, 1C), 112.2 (s, 1C, C4), 49.0 (s, 1C, C3).

Cu4 (R = 2-pyridilmethyl)

177.5 mg. Yield 69 %. HRMS (ESI-QTOF) m/z: $[M]^+$ Calculated for $C_{42}H_{42}B_9CuN_4P_2S$ 858.2733; Found 858.2707.

^{1}H NMR (400.1 MHz, acetone-d$_6$, rt, ppm) δ = 8.71 (d, *J* = 1.7 Hz, 1H, H2), 8.43 (dq, *J* = 4.8, 0.9 Hz, 1H, H5), 7.87 (d, *J* = 8.2 Hz, 1H, H4), 7.78 (td, *J* = 7.7, 1.8 Hz, 1H), 7.67 – 7.11 (m, 26H), 6.07 (s, 2H, H3), -1.84 (br s, 1H, H-BHB). ^{31}P{^{1}H} NMR (162.0 MHz, acetone-d$_6$, rt, ppm) δ = 17.0. ^{13}C{^{1}H} APT (100.6 MHz, acetone-d$_6$, rt, ppm*)* δ = 155.6 (s, 1C), 150.7 (s, 1C), 140.7 (s, 1C), 138.4 (s, 1C), 137.5 (m, 2C), 137.3 (t, *J* = 15.0 Hz, 2C), 137.1 (s, 1C), 136.4 (t, *J* = 19.0 Hz, 2C), 135.8 (t, *J* = 9.8 Hz, 4C), 133.5 (t, *J* = 7.6 Hz, 4C), 130.9 (s, 1C), 129.2 (s, 1C), 128.9 – 128.5 (m, 12C), 125.8 (s, 1C), 124.3 (s, 1C), 123.0 (s, 1C), 122.0 (s, 1C), 119.8 (s, 1C), 122.2 (s, 1C), 50.7 (s, 1C, C3).

Cu5 (R = 2-naphthylmethyl)

170.0 mg. Yield: 63 %. HRMS (ESI-QTOF) m/z: $[M+Na]^+$ Calculated for $C_{47}H_{45}B_9CuN_3NaP_2S$ 930.2835; Found 930.2865. Anal. (%) calcd. for $C_{47}H_{45}B_9Cu_4N_3P_2S{\cdot}1/4\ CH_2Cl_2$: C, 61.2; H, 4.9; N, 4.5; S, 3.5. Found: C, 61.6; H, 5.4; N, 4.6; S, 3.5

^{1}H NMR (400.1 MHz, acetone-d$_6$, rt, ppm) δ = 8.45 (d, J = 1.6 Hz, 1H, H2), 7.88 – 7.81 (m, 3H), 7.64 – 7.06 (m, 29H), 6.22 (s, 2H, H3), -1.85 (br s, 1H, H-BHB). ^{31}P{^{1}H} NMR (162.0 MHz, acetone-d$_6$, rt, ppm) δ = 16.9. ^{13}C{^{1}H} APT (100.6 MHz, acetone-d$_6$, rt, ppm) δ = 147.3 (s, 1C), 143.6 (s, 1C), 140.8 (s, 1C), 137.7 (s, 1C), 137.3 (t, J = 15.0 Hz, 2C), 136.4 (t, J = 19.0 Hz, 2C), 135.8 (t, J = 9.9 Hz, 4C) 134.2 (s, 1C), 133.8 (s, 1C), 133.7 (s, 1C), 133.4 (t, J = 7.5 Hz, 4C), 130.9 (s, 1C), 130.0 (s, 1C), 129.0 – 128.4 (m, 14C), 127.5 (s, 1C), 127.3 (s, 1C), 126.0 (s, 1C), 125.6 (s, 1C), 125.3 (s, 1C), 124.9 (s, 1C), 121.8 (s, 1C, C2), 119.9 (s, 1C), 112.3 (s, 1C), 49.2 (s, 1C, C3).

Cu6 (R = 2-quinolylmethyl)

197.0 mg. Yield: 72 %. HRMS (ESI-QTOF) m/z: $[M+Na]^+$ Calculated for $C_{46}H_{44}B_9CuN_4NaP_2S$ 931.2788; Found 931.2822. Anal. (%) calcd. for $C_{46}H_{44}B_9Cu_4N_3P_2S{\cdot}1/4\ CH_2Cl_2$: C, 59.8; H, 4.3; N, 6.0; S, 3.5. Found: C, 59.6; H, 4.8; N, 6.1; S, 3.3

^{1}H NMR (400.1 MHz, acetone-d$_6$, rt, ppm) δ = 8.77 (s, 1H, H2), 8.32 (dd, J = 8.6, 3.2 Hz, 1H, H4), 7.98 – 7.86 (m, 2H), 7.75 – 7.12 (m, 28H), 6.30 (d, J = 3.2 Hz, 2H, H3), -1.89 (br s, 1H, H-BHB). ^{31}P{^{1}H} NMR (162.0 MHz, acetone-d$_6$, rt, ppm) δ = 20.3. ^{13}C{^{1}H} APT (100.6 MHz, acetone-d$_6$, rt, ppm) δ = 155.7 (s, 1C), 148.3 (s, 1C), 147.9 (s, 1C), 144.1 (s, 1C), 140.8 (s, 1C), 138.6 (s, 1C, C4), 137.7 (s, 1C), 137.3 (t, J = 14.9 Hz, 2C), 136.5 (t, J = 19.1 Hz, 2C), 135.8 (t, J = 9.9 Hz, 4C), 133.4 (t, J = 7.5 Hz, 4C), 130.9 (s, 1C), 130.8 (s, 1C), 129.8 (s, 1C), 129.2 (s, 1C), 128.9 – 128.5 (m, 14C), 128.4 (s, 1C), 127.8 (s, 1C), 125.8 (s, 1C), 120.4 (s, 1C, C2), 119.8 (s, 1C), 112.2 (s, 1C), 49.2 (s, 1C, C3).

Cu8 (R = propargyl)

190.3 mg. Yield: 79%. HRMS (ESI-QTOF) m/z: $[M+Na]^+$ Calculated for $C_{39}H_{39}B_9CuN_3NaP_2S$ 828.2366; Found 958. 828.2371.

^{1}H NMR (300.1 MHz, acetone-d$_6$, rt, ppm) δ = 8.78 (d, J = 1.7 Hz, 1H, H2), 7.92 (d, J = 8.2 Hz, 1H), 7.64 – 7.13 (m, 22H), 5.66 (d, J = 2.6 Hz, 2H, H3), 3.14 (t, J = 2.6 Hz, 1H, H4), -1.98 (br s, 1H, H-BHB). ^{31}P{^{1}H} NMR (121.5 MHz, acetone-d$_6$, rt, ppm) δ = 16.4.

Cu9 (R = benzyl)

165.1 mg. Yield: 64%. HRMS (ESI-QTOF) m/z: $[M+Na]^+$ Calculated for $C_{43}H_{43}B_9CuN_3NaOP_2$ 864.2912; Found 864.2963.

^1H NMR (300.1 MHz, acetone-d_6, rt, ppm) δ = 8.96 (s, 1H, H2), 7.78 – 6.96 (m, 30H), 5.89 (s, 2H, H3), -1.75 (br s, 1H, H-BHB). $^{31}P\{^1H\}$ NMR (121.50 MHz, acetone-*d₆*, *rt,* ppm) δ = 15.2. $^{13}C\{^1H\}$ APT (75.48 MHz, acetone-d_6, rt, ppm) δ = 153.8 (s, 1C), 141.2 (s, 1C), 137.4 (t, *J* = 15.1 Hz, 2C), 137.3 (s, 1C), 136.5 (t, *J* = 19.0 Hz, 2C), 136.0 (s,1C), 135.9 (t, *J* = 9.8 Hz, 4C), 133.7 (t, *J* = 7.5 Hz, 4C), 131.0 (s, 1C), 130.1 (s, 1C), 129.5 (s, 1C), 129.1 (s, 1C), 129.0 – 128.6 (m, 14C), 127.5 (s, 1C), 125.8 (s, 1C), 124.9 (s, 1C), 120.1 (s, 1C), 112.2 (s, 1C), 49.2 (s, 1C, C3).

Synthesis of complex Cu7

To a solution of $[Cu(CH_3CN)_4](PF_6)$ (0.1 mmol, 37.3 mg) in dichloromethane was added $[NBu_4][7,8\text{-}(PPh_2)_2\text{-}7,8\text{-}C_2B_9H_{10}]$ (0.1 mmol, 76.1 mg). The mixture was stirred for 30 min, and **L7** (0.1 mmol, 39.4 mg) was added. The mixture was stirred for 1 h. Then, it was evaporated to a minimum volume. Addition of ethanol (10 mL) led to the precipitation of **Cu7** as a yellow solid, which was filtered, washed with more ethanol (20 mL) and dried under vacuum. Yield: 76.0 mg, 78 %.

HRMS (ESI-QTOF) m/z: $[M+Na]^+$ Calculated for $C_{51}H_{47}B_9CuN_3NaP_2S$ 980.2997; Found 980.3028.

^1H NMR (400.1 MHz, DMSO-d_6, rt, ppm) δ = 9.24 (s, 1H, H2), 8.54 (s, 1H, H1), 8.43 (d, *J* = 7.5 Hz, 1H), 8.18 – 8.09 (m, 6H), 7.96 – 7.85 (m, 9H), 7.79 – 7.55 (m, 19H), -1.41 (br s, 1H, H-BHB). $^{31}P\{^1H\}$ NMR (162.0 MHz, DMSO-d_6, rt*,* ppm) δ = 18.8.

Synthesis of complexes Cu10 and Cu11.

To a solution of $[Cu(CH_3CN)_4](PF_6)$ (0.3 mmol, 111.8 mg) in dichloromethane was added $[1,2\text{-}(PPh_2)_2\text{-}1,2\text{-}C_2B_{10}H_{10}]$ (0.3 mmol, 153.8 mg). The mixture was stirred for 30 min, and the corresponding substituted derivative of 2-(4-thiazolyl)benzimidazole (0.3 mmol; **L3**: 87.4 mg, **L8**: 71.7 mg) was added. The mixture was stirred for 1 h. Then, it was evaporated to a minimum volume. Addition of n-hexane (10 mL) led to the precipitation of a yellow solid, which was filtered and dried under vacuum.

Cu10 (R = benzyl)

235.3 mg. Yield: 77%. HRMS (ESI-QTOF) m/z: $[M]^+$ Calculated for $C_{43}H_{43}B_{10}CuN_3P_2S$ 868.2874; Found 868.2905.

^1H NMR (300.1 MHz, acetone-d_6, rt, ppm) δ = 9.56 (d, *J* = 1.1 Hz, 1H, H1/H2), 8.65 (d, *J* = 1.1 Hz, 1H, H1/H2), 8.00 – 7.21 (m, 29H), 6.18 (s, 2H, H3). $^{31}P\{^1H\}$ NMR (121.5 MHz, acetone-*d₆*, rt*,* ppm) δ = 13.3. $^{13}C\{^1H\}$ APT (75.5 MHz, acetone-d_6, rt, ppm) δ = 159.0 (*, 1C, C1/C2),148.8 (s, 1C), 143.8 (s, 1C), 140.4 (s, 1C), 138.0 (s, 1C), 135.7 (t, *J* = 10.5 Hz, 8C), 133.3 (s, 1C), 130.3 – 130.0 (m, 14C), 129.5 (t, *J* = 15.8 Hz, 4C), 129.0 (s, 1C), 127.0 (s, 1C), 126.4 (s, 1C), 125.8 (s, 1C), 123.5 (s, 1C, C1/C2),

119.3 (s, 1C), 113.0 (s, 1C), 87.4 (s, 1C), 49.2 (s, 1C, C3). ^{19}F NMR (282.4 MHz, acetone-d$_6$, rt, ppm): δ = -72.2 (d, J = 707.6 Hz).

* = Signal determined by HSQC (^{1}H-^{13}C{^{1}H}) experiment.

Cu11 (R = propargyl)

201.5 mg. Yield: 67%. HRMS (ESI-QTOF) m/z: [M]$^+$ Calculated for $C_{39}H_{39}B_{10}CuN_3P_2S$ 816.2561; Found 816.2600.

^{1}H NMR (300.1 MHz, acetone-d$_6$, rt, ppm) δ = 9.47 (d, J = 1.6 Hz, 1H, H1/H2), 8.90 (d, J = 1.6 Hz, 1H, H1/H2), 7.94 (dt, J = 8.3, 0.9 Hz, 1H), 7.77 – 7.67 (m, 8H), 7.61 – 7.38 (m, 8H), 7.37 – 7.28 (m, 8H), 5.64 (d, J = 2.6 Hz, 2H, H3), 3.12 (t, J = 2.6 Hz, 1H, H4). ^{31}P{^{1}H} NMR (121.5 MHz, acetone-d$_6$, rt, ppm) δ = 14.0. ^{13}C{^{1}H} APT (75.5 MHz, acetone-d$_6$, rt, ppm) δ = 159.5 (*, 1C, C1/C2),147.5 (s, 1C), 143.7 (s, 1C), 140.1 (s, 1C), 136.9 (s, 1C), 135.7 (t, J = 10.4 Hz, 8C), 133.3 (s, 1C), 130.3 – 130.1 (m, 12C), 129.5 (t, J = 15.8 Hz, 4C), 126.4 (s, 1C), 125.9 (s, 1C), 124.0 (s, 1C, C1/C2),119.3 (s, 1C), 112.7 (s, 1C), 87.3 (s, 1C), 77.2 (s, 1C), 35.8 (s, 1C, C3). ^{19}F NMR (282.4 MHz, acetone-d$_6$, rt, ppm): δ = -73.7 (d, J = 707.7 Hz).

* = Signal determined by HSQC (^{1}H-^{13}C{^{1}H}) experiment.

S2.- NMR spectra

L1

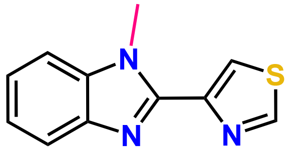

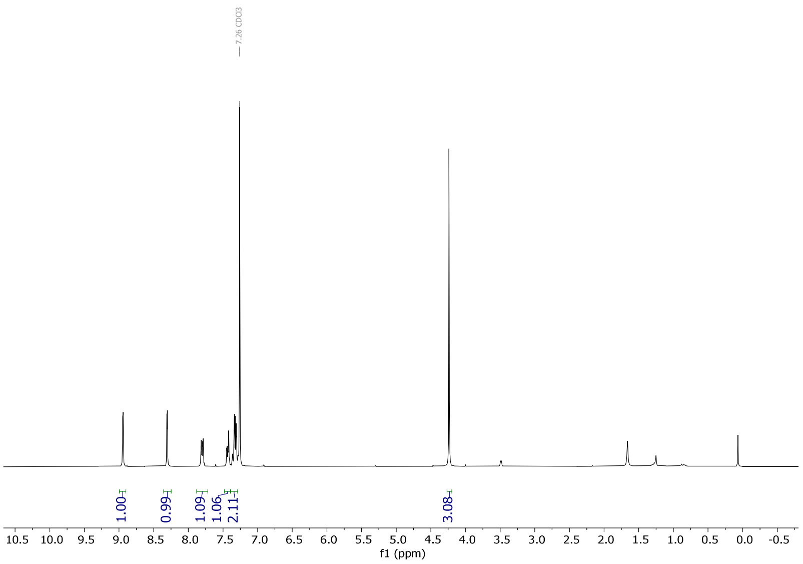

Figure S3. ^{1}H NMR spectrum of **L1**.

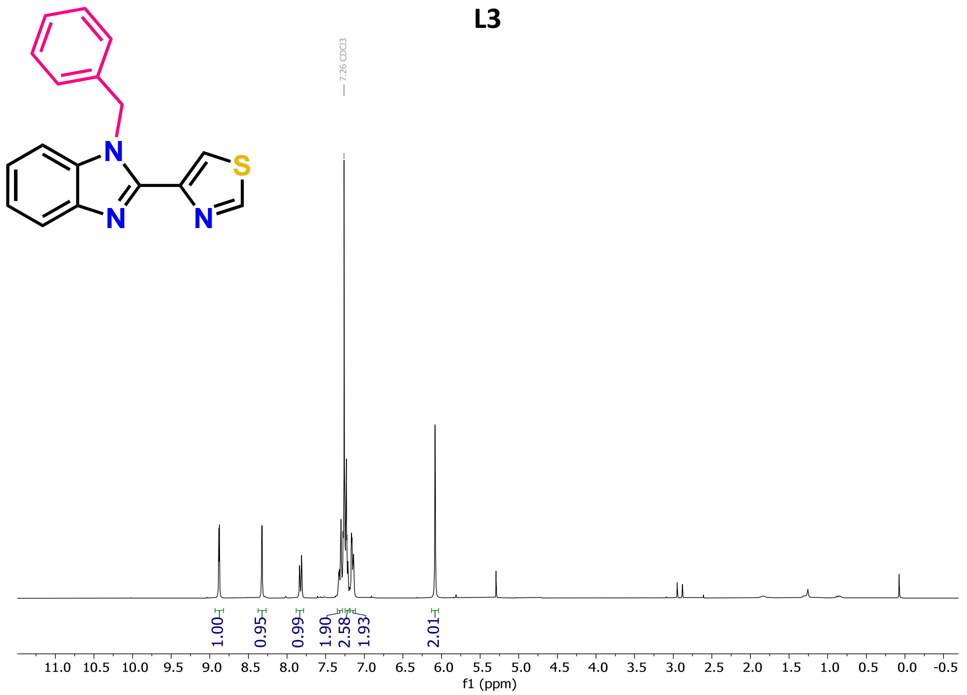

Figure S4. ^{1}H NMR spectrum of **L3**.

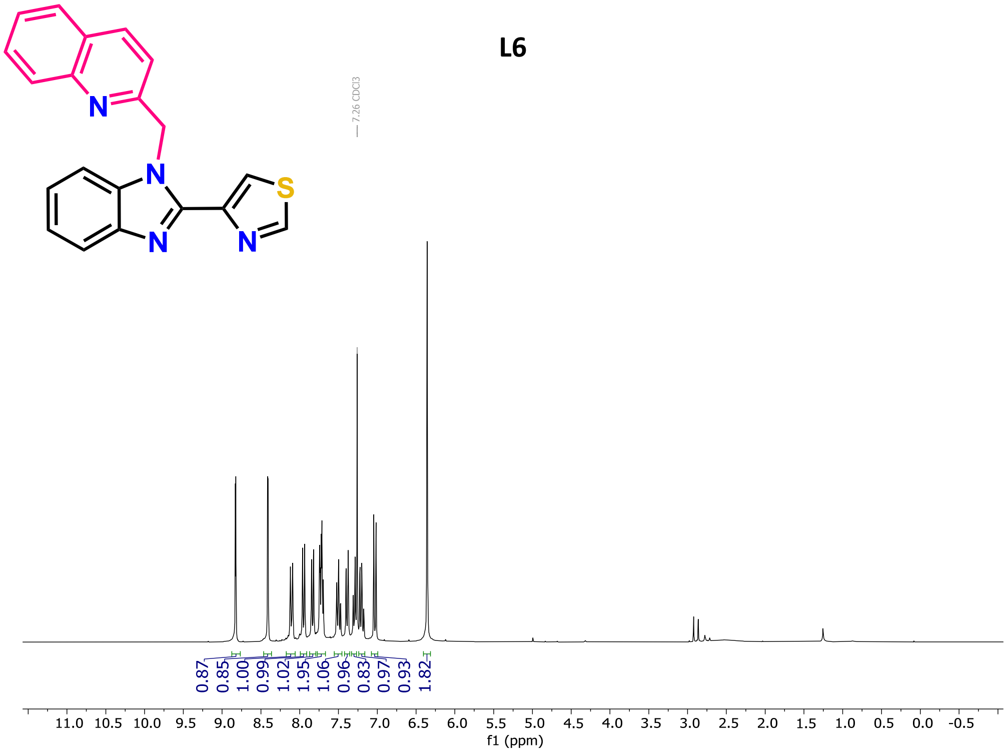

Figure S5. ^{1}H NMR spectrum of **L6**.

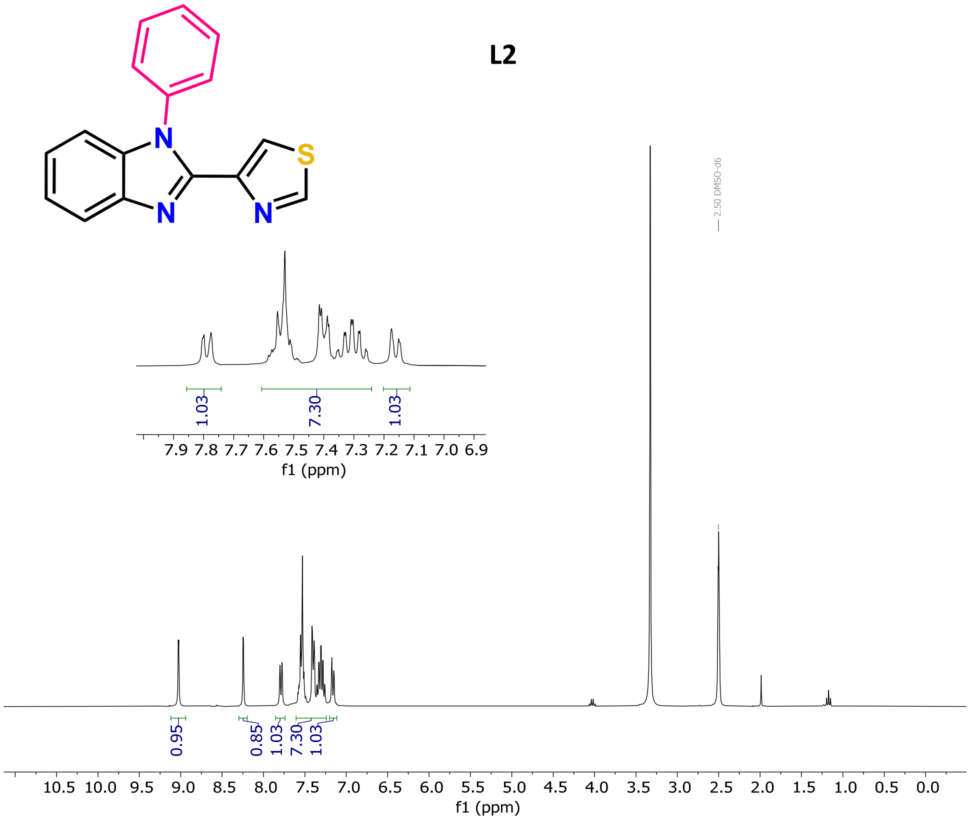

Figure S6. ^{1}H NMR spectrum of **L2**.

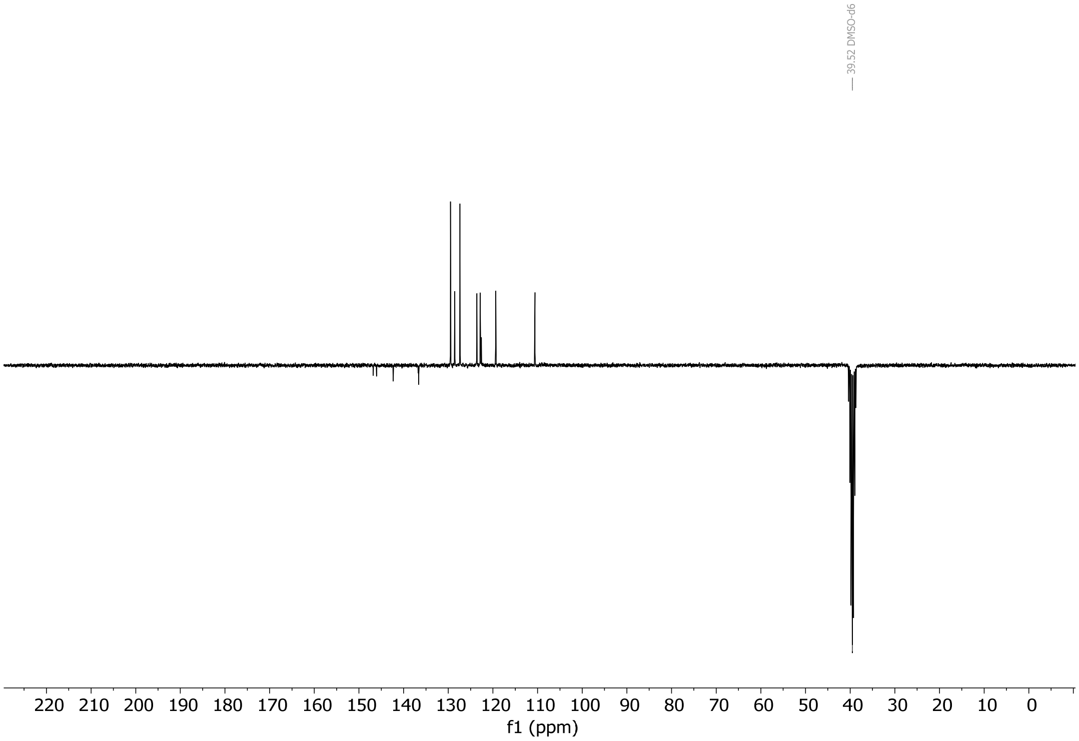

Figure S7. $^{13}C\{^{1}H\}$-APT NMR spectrum of **L2**.

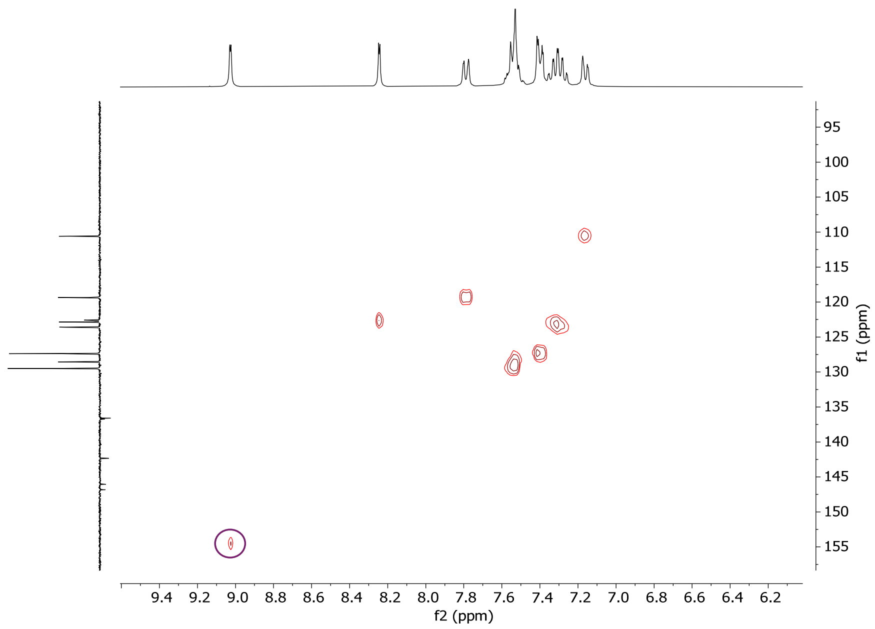

Figure S8. HSQC ^{1}H-$^{13}C\{^{1}H\}$ NMR spectrum of **L2**.

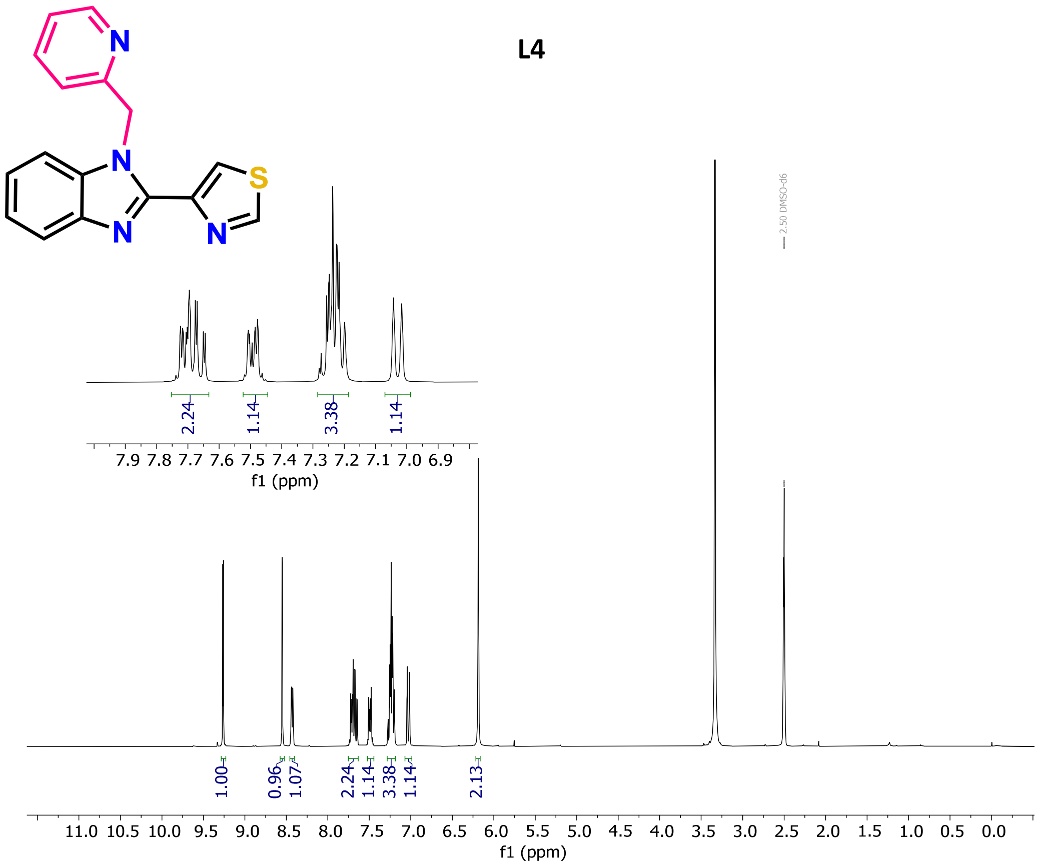

Figure S9. ^{1}H NMR spectrum of **L4**.

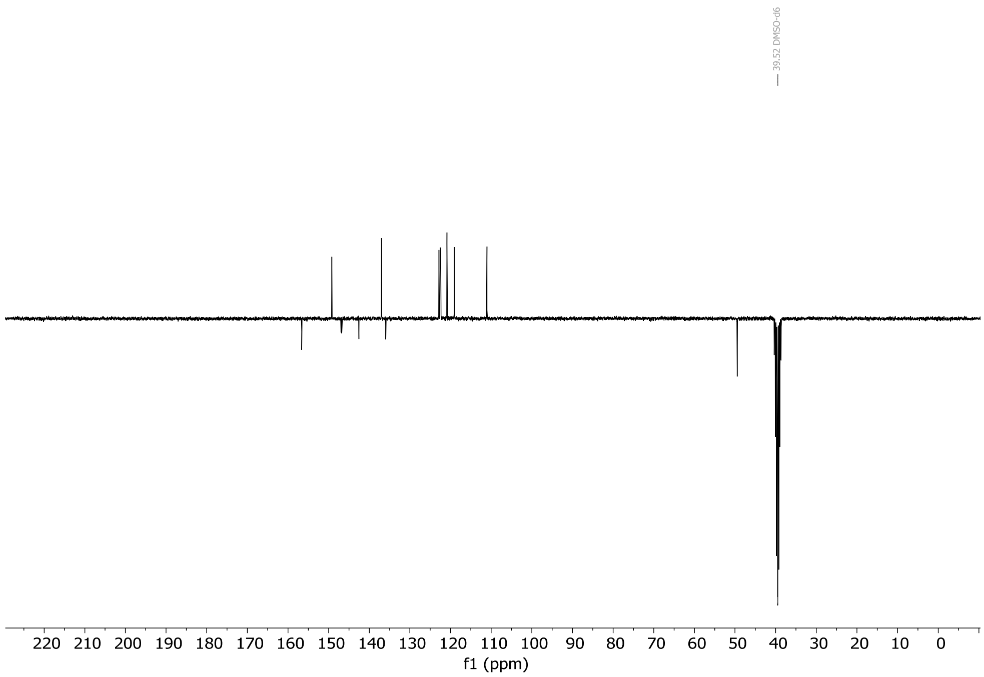

Figure S10. $^{13}C\{^{1}H\}$-APT NMR spectrum of **L4**.

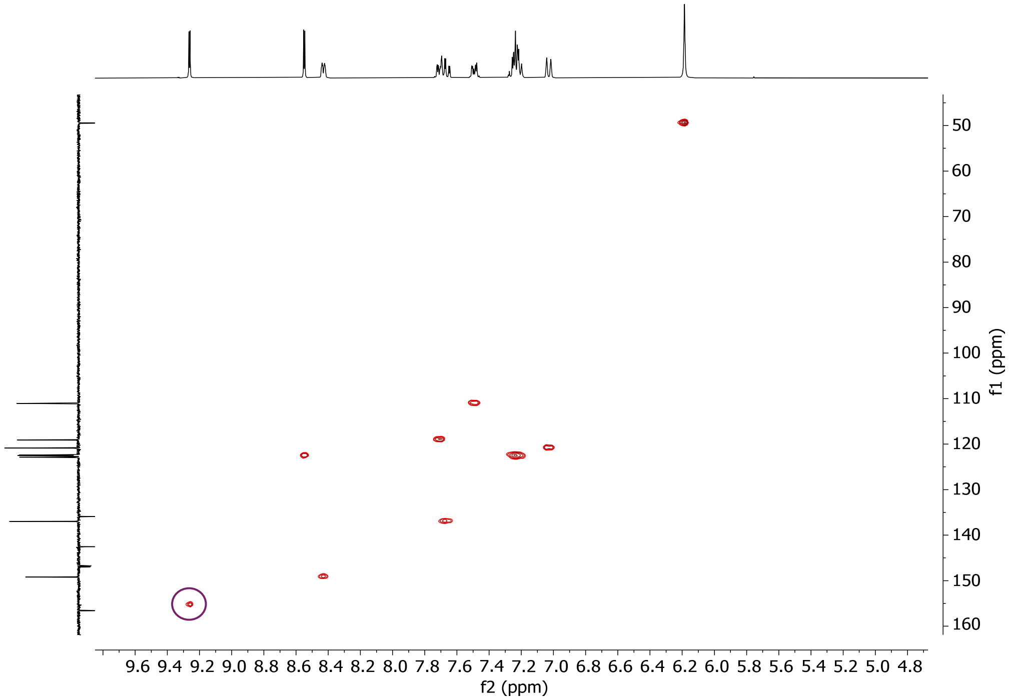

Figure S11. HSQC ^{1}H-$^{13}C\{^{1}H\}$ NMR spectrum of **L4**.

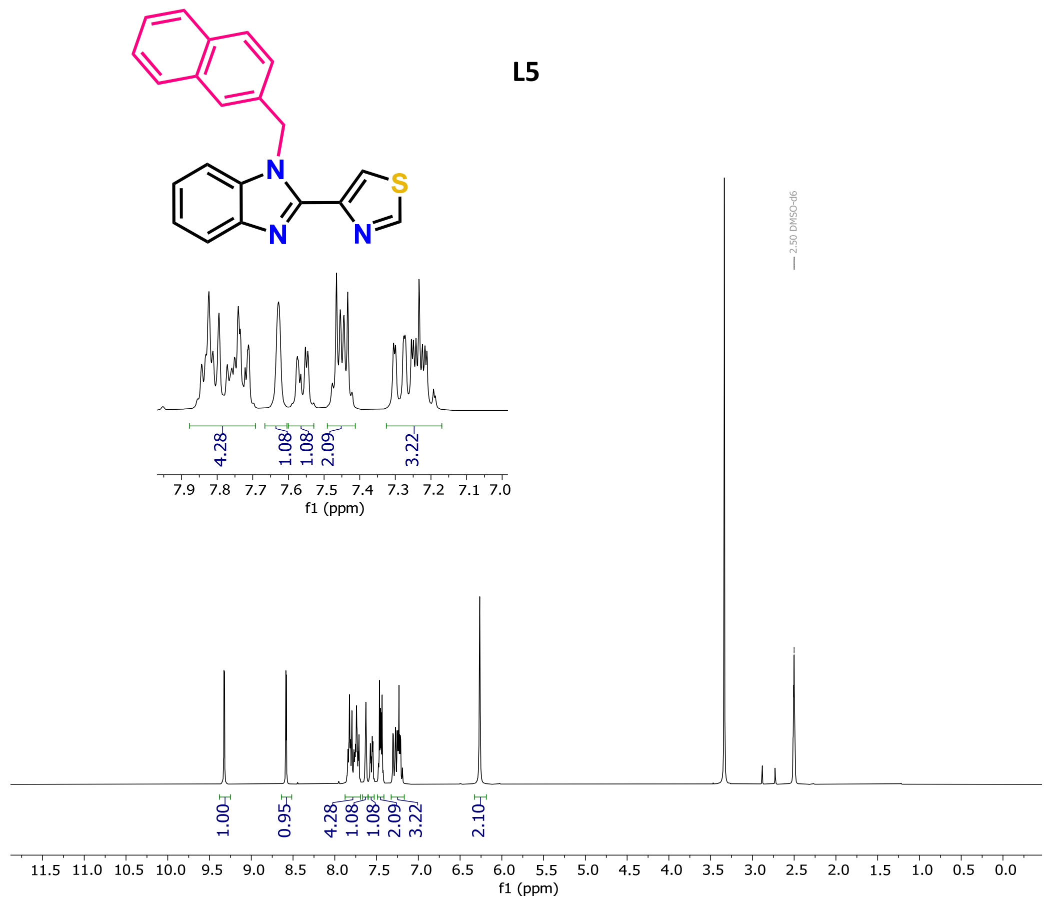

Figure S12. ^{1}H NMR spectrum of **L5**.

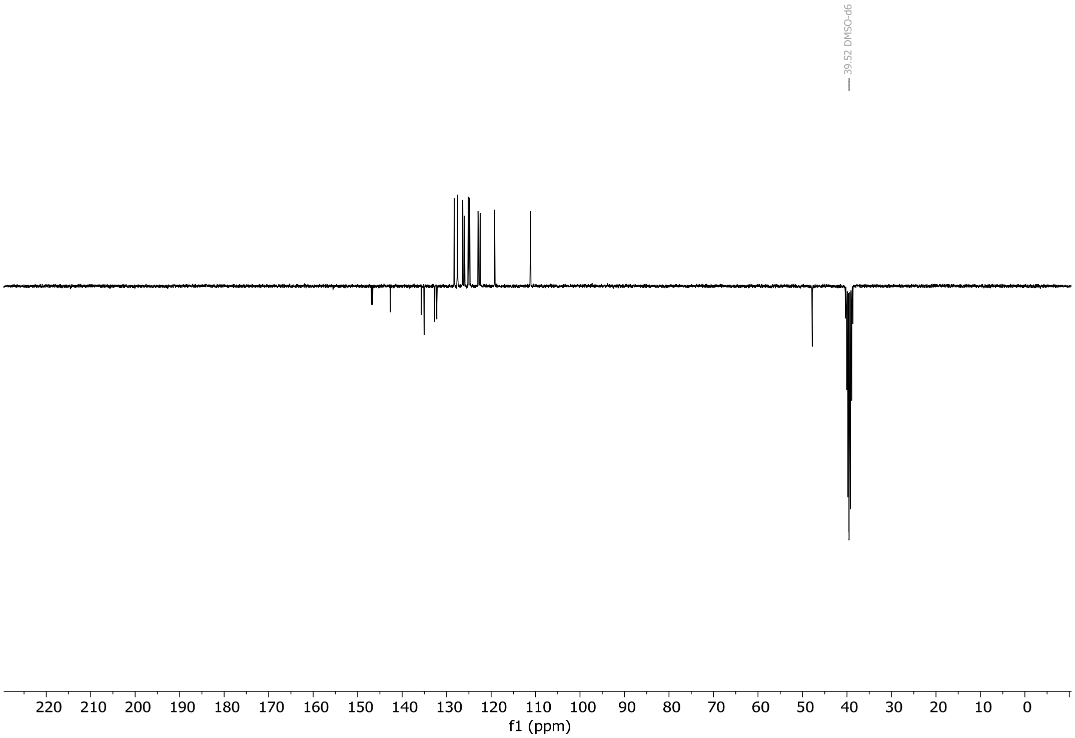

Figure S13. ^{13}C{^{1}H}-APT NMR spectrum of **L5**.

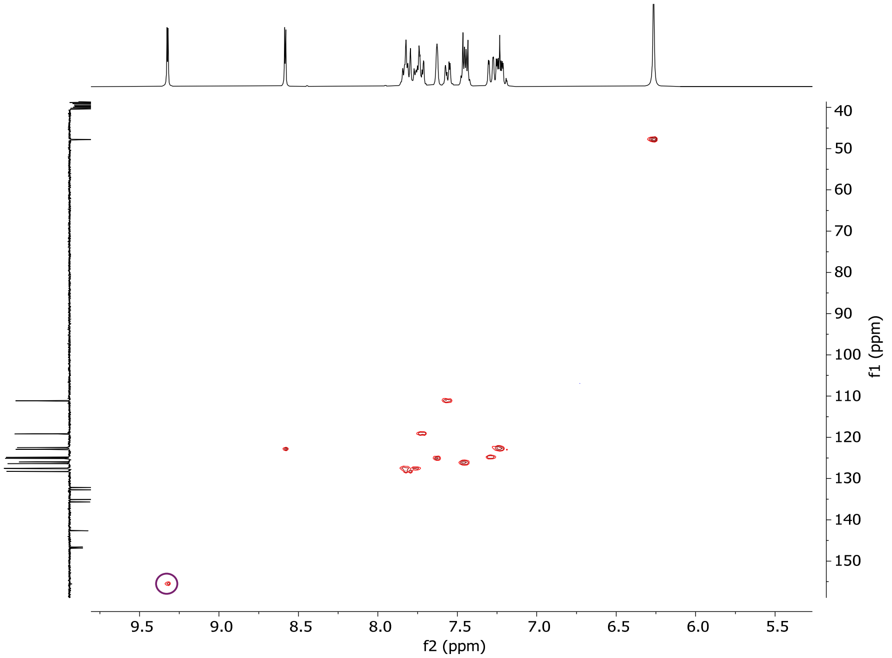

Figure S14. HSQC ^{1}H-$^{13}C\{^{1}H\}$ NMR spectrum of **L5**.

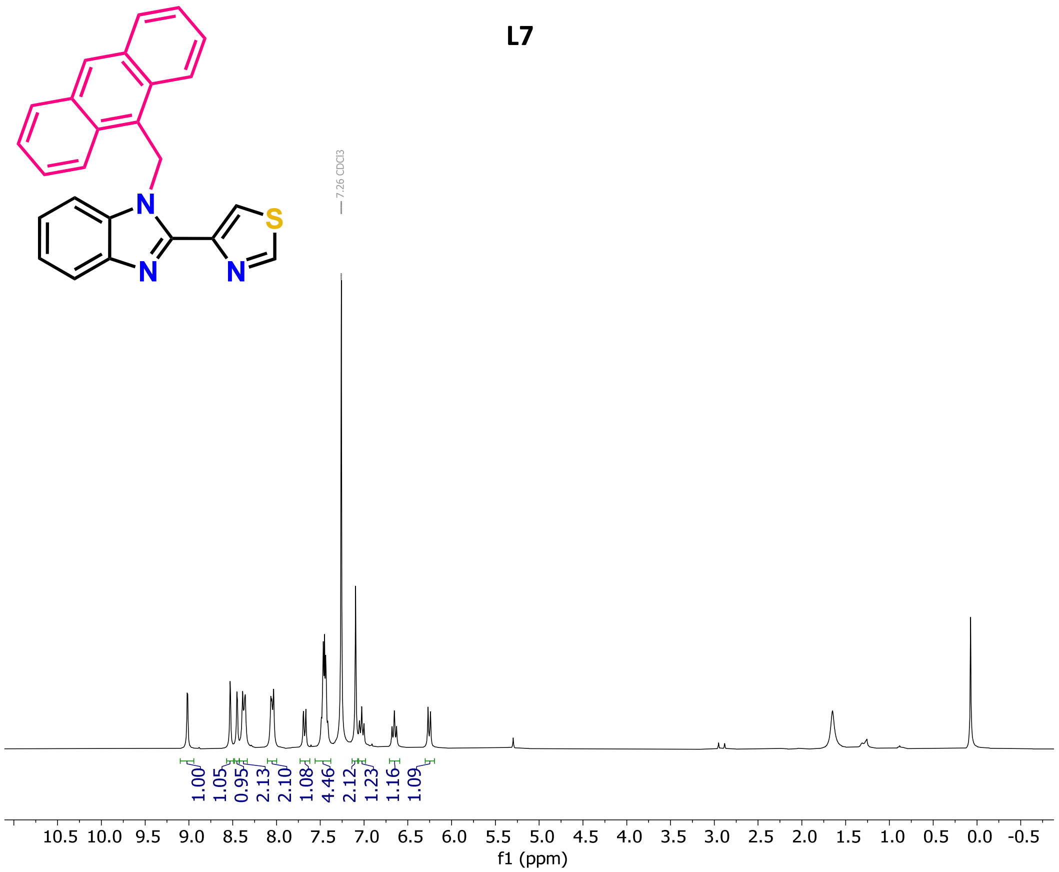

Figure S15. ^{1}H NMR spectrum of **L7**.

L9

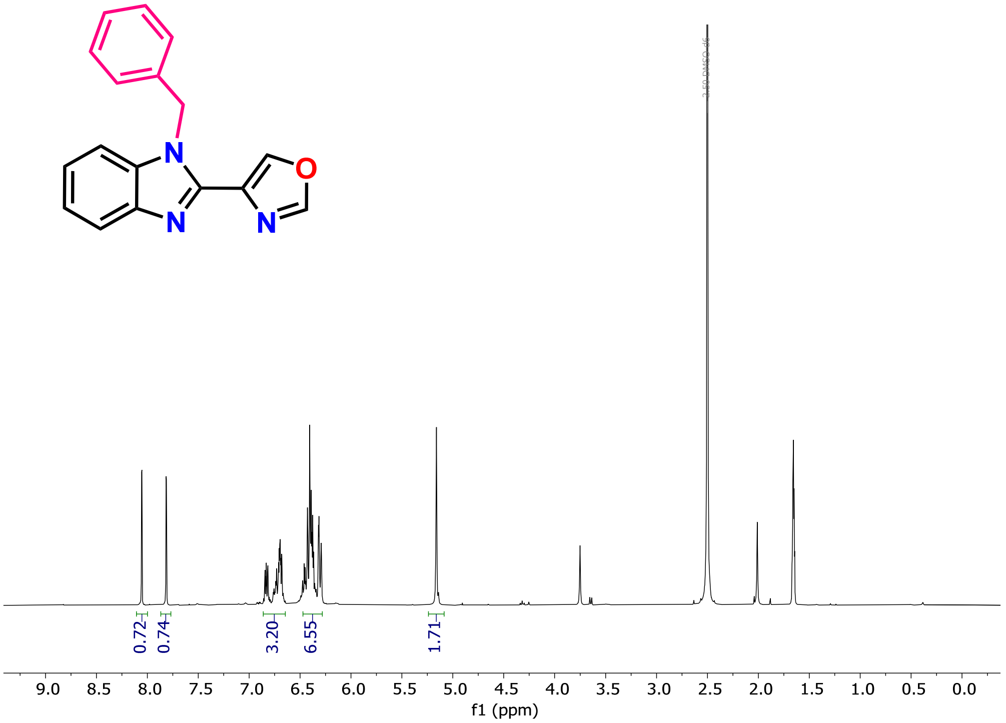

Figure S16. ^{1}H NMR spectrum of **L9**.

Cu1

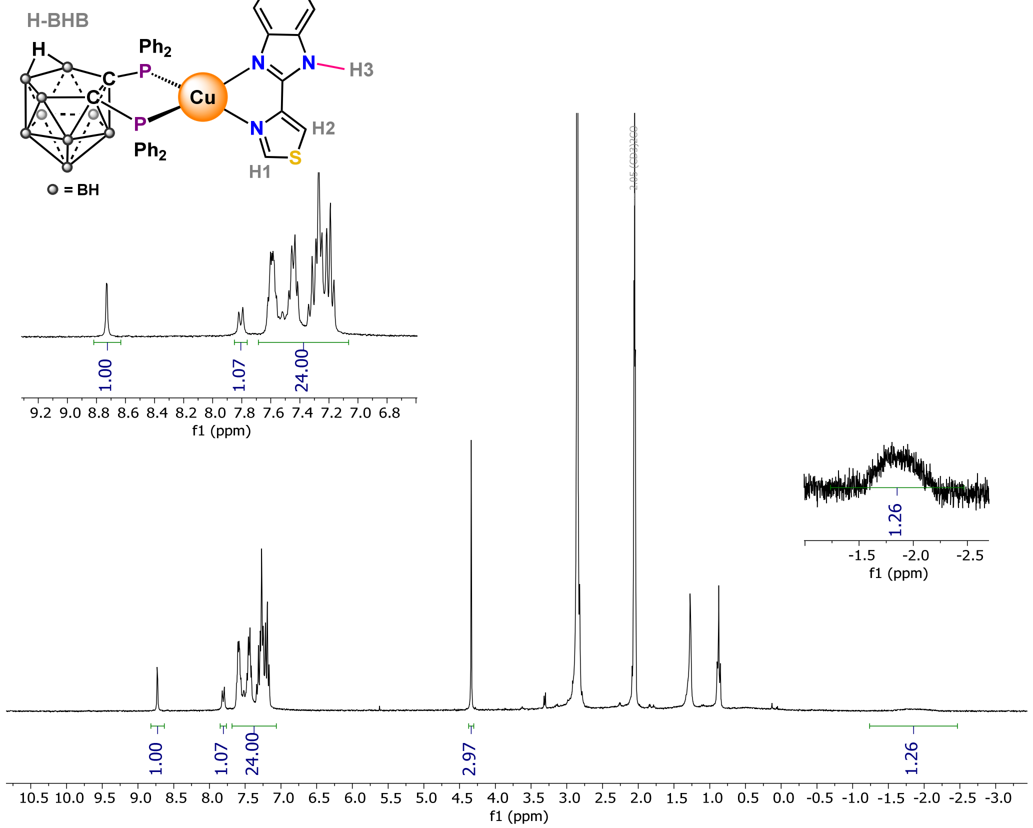

Figure S17. ^{1}H NMR spectrum of **Cu1**.

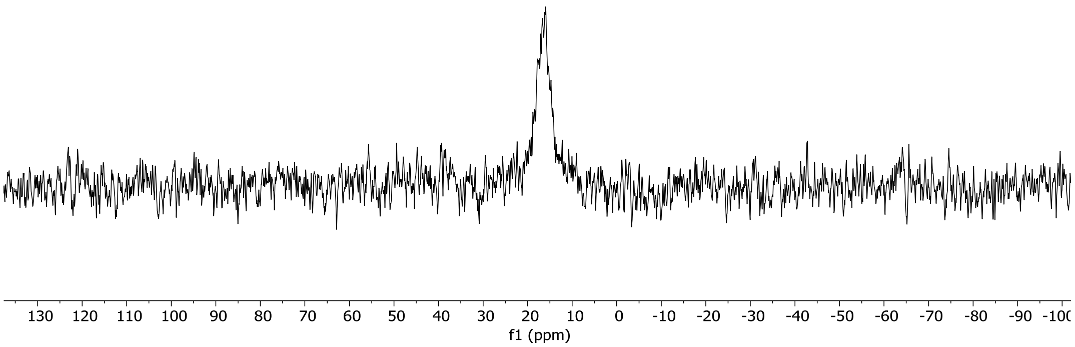

Figure S18. $^{31}P\{^{1}H\}$ NMR spectrum of **Cu1**

Cu2

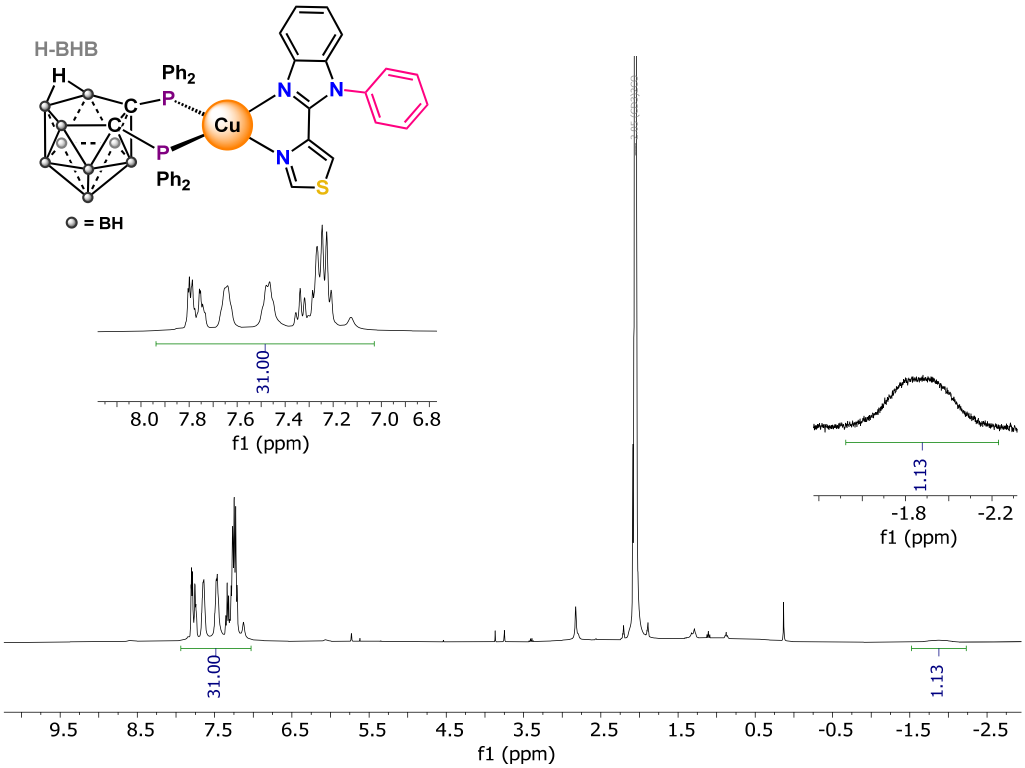

Figure S19. ^{1}H NMR spectrum of **Cu2**.

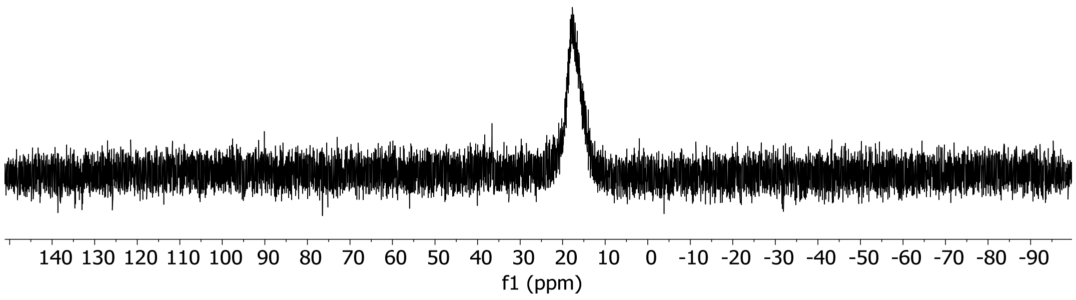

Figure S20. $^{31}P\{^{1}H\}$ NMR spectrum of **Cu2**.

Cu3

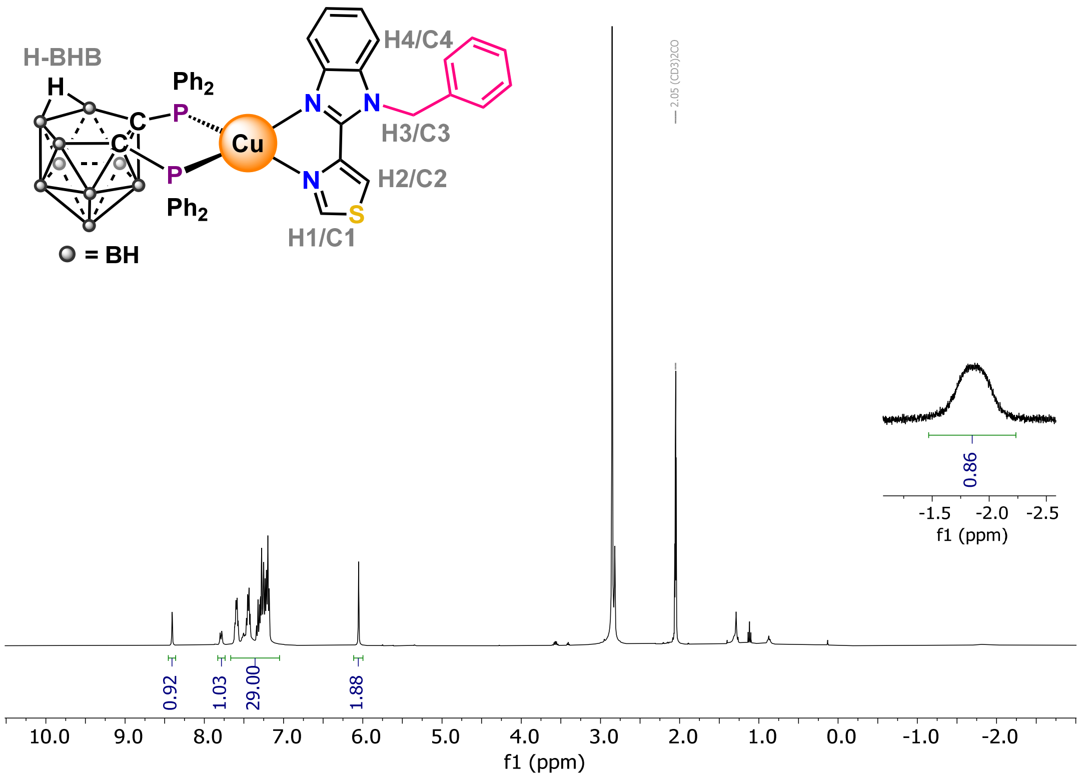

Figure S21. ^{1}H NMR spectrum of **Cu3**.

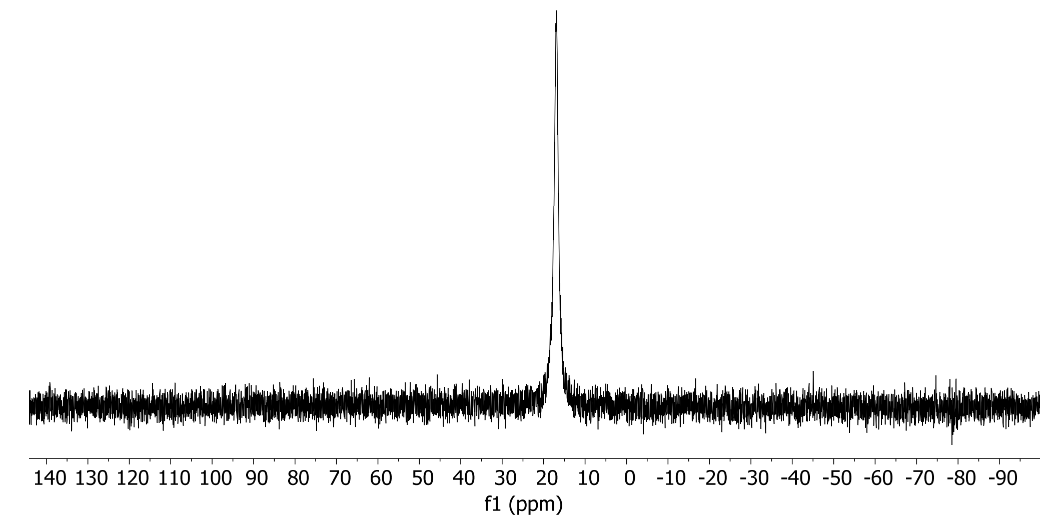

Figure S22. $^{31}P\{^{1}H\}$ NMR spectrum of **Cu3**.

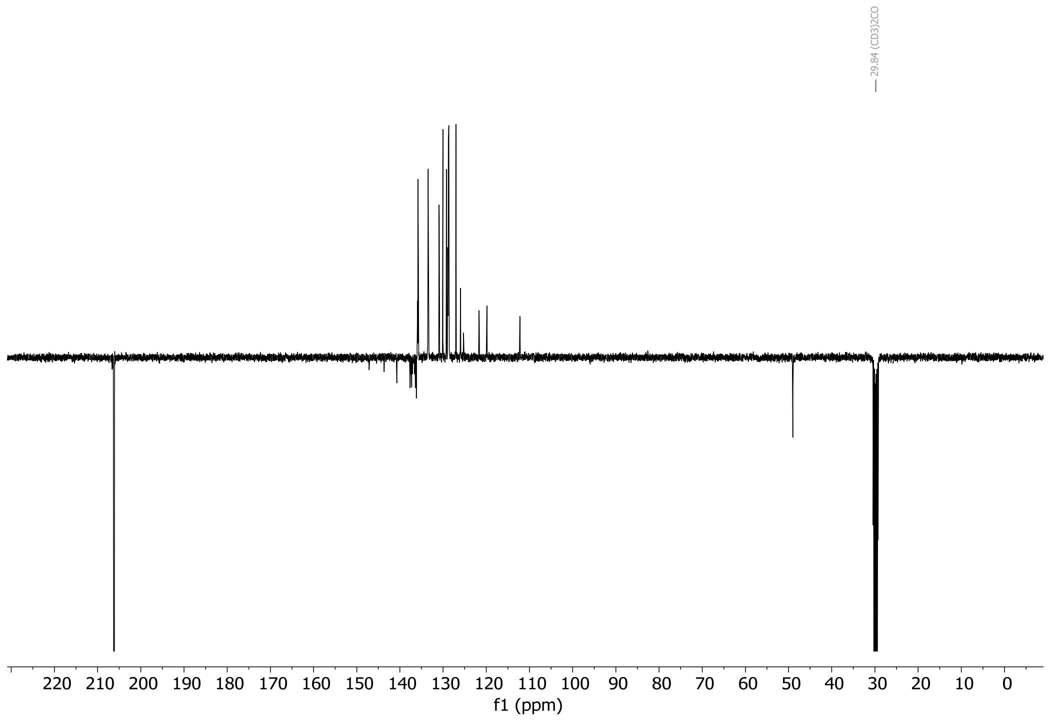

Figure S23. $^{13}C\{^{1}H\}$-APT NMR spectrum of **Cu3**.

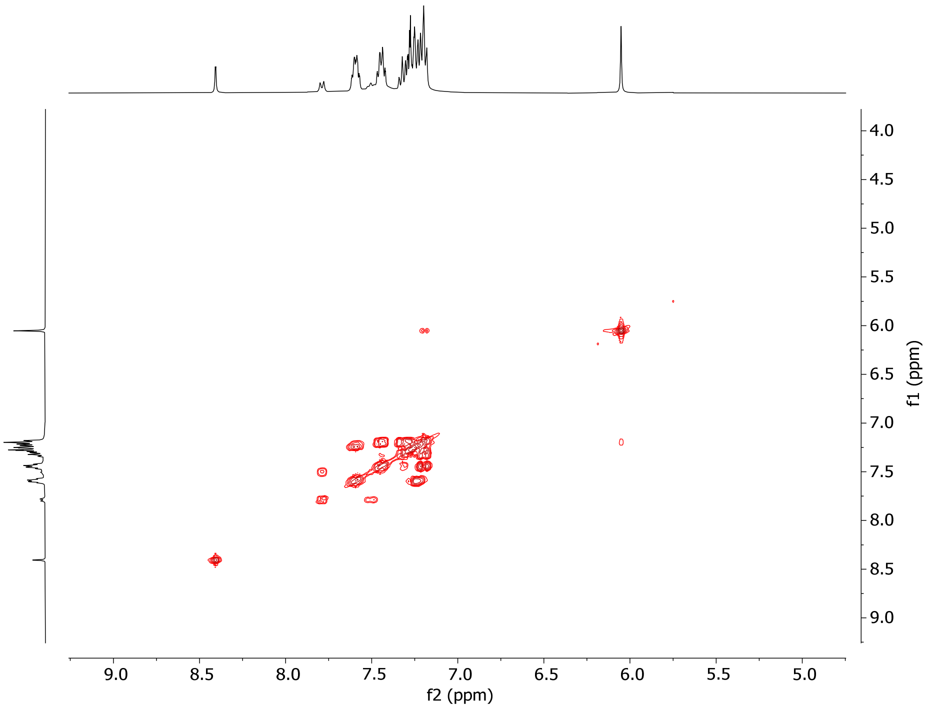

Figure S24. COSY ^{1}H-^{1}H NMR spectrum of **Cu3**.

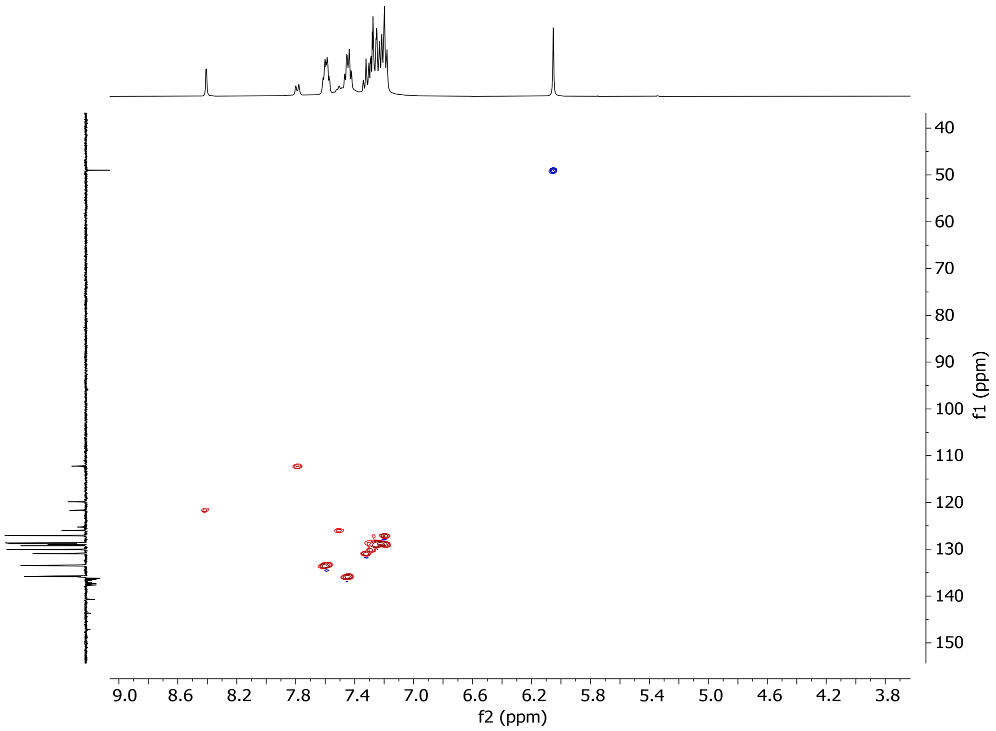

Figure S25. HSQC ^{1}H-$^{13}C\{^{1}H\}$ NMR spectrum of **Cu3**.

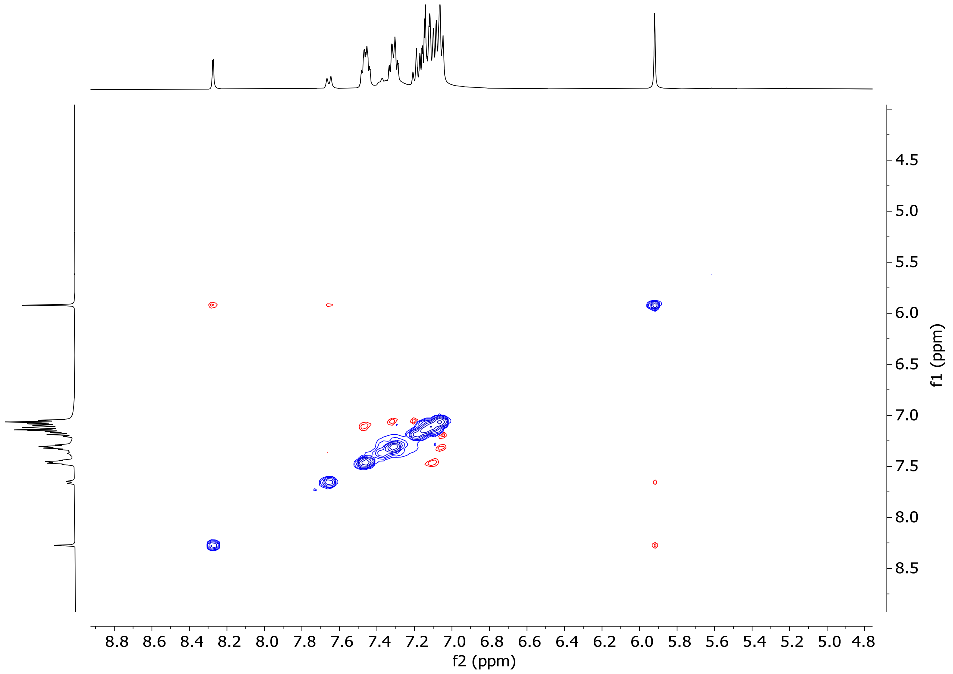

Figure S26. NOESY ^{1}H-^{1}H NMR spectrum of **Cu3**.

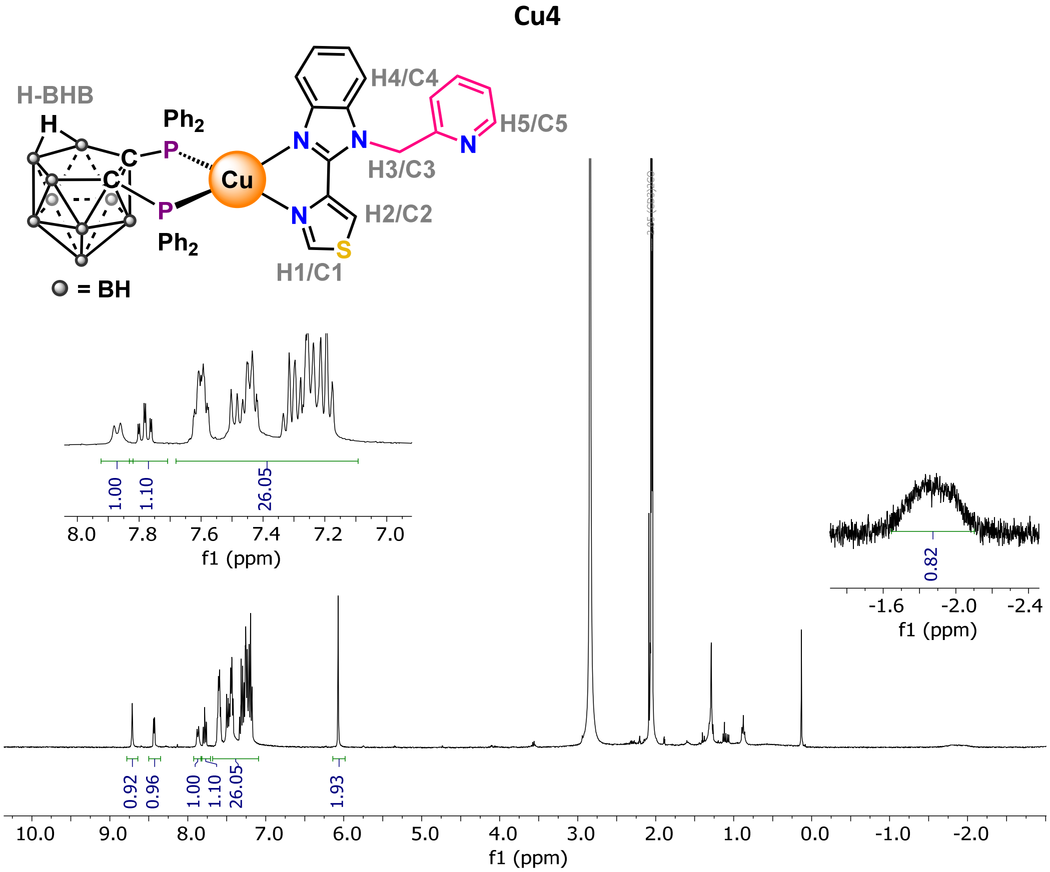

Figure S27. ^{1}H NMR spectrum of **Cu4**.

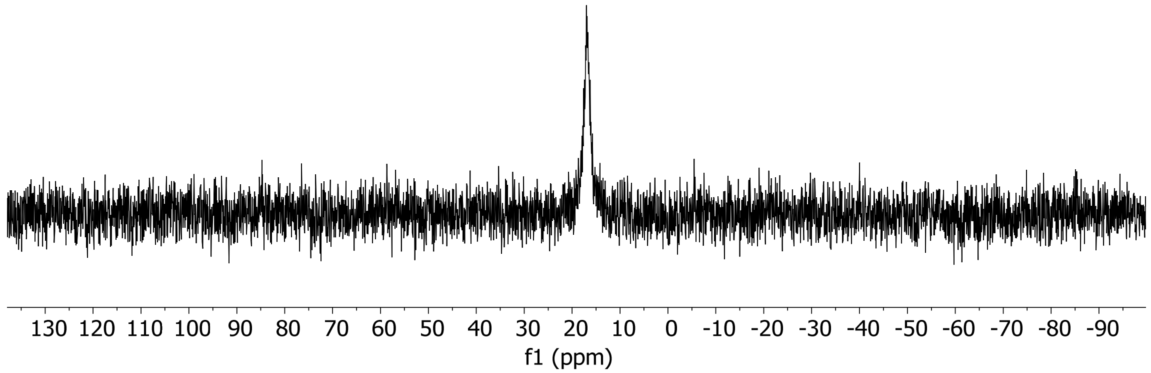

Figure S28. $^{31}P\{^{1}H\}$ NMR spectrum of **Cu4**.

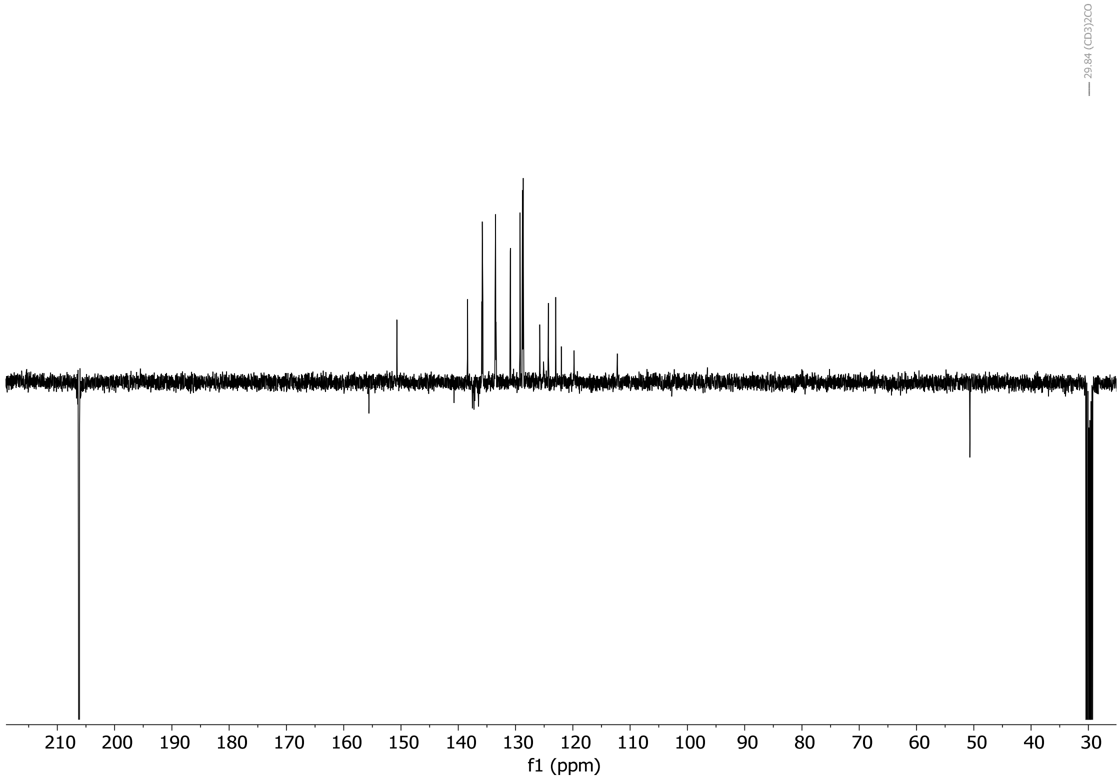

Figure S29. $^{13}C\{^{1}H\}$-APT NMR spectrum of **Cu4**.

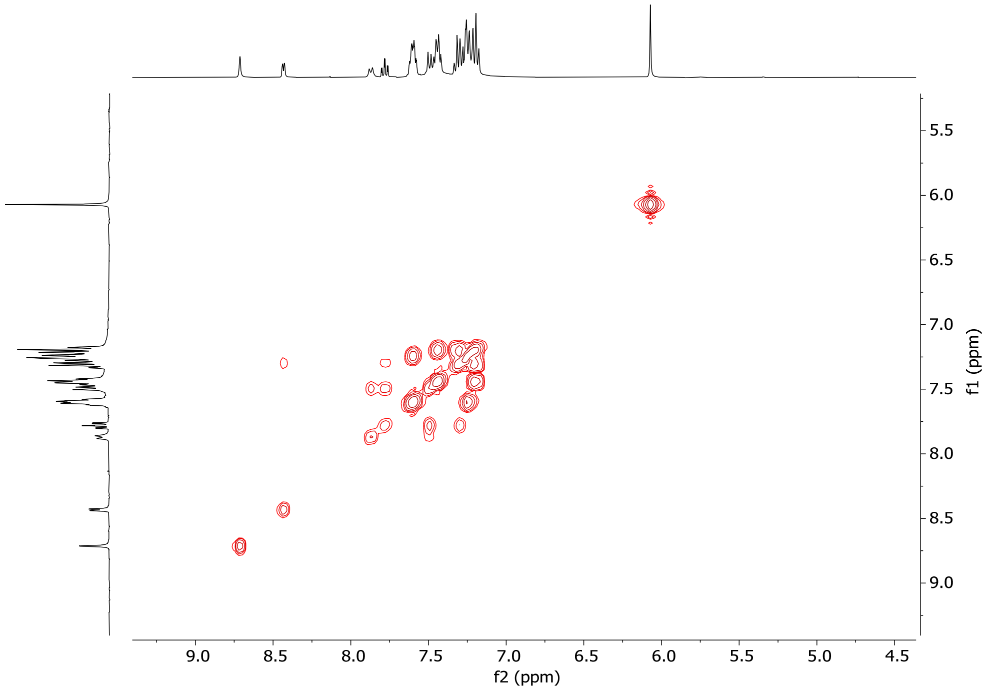

Figure S30. COSY ^{1}H-^{1}H NMR spectrum of **Cu4**.

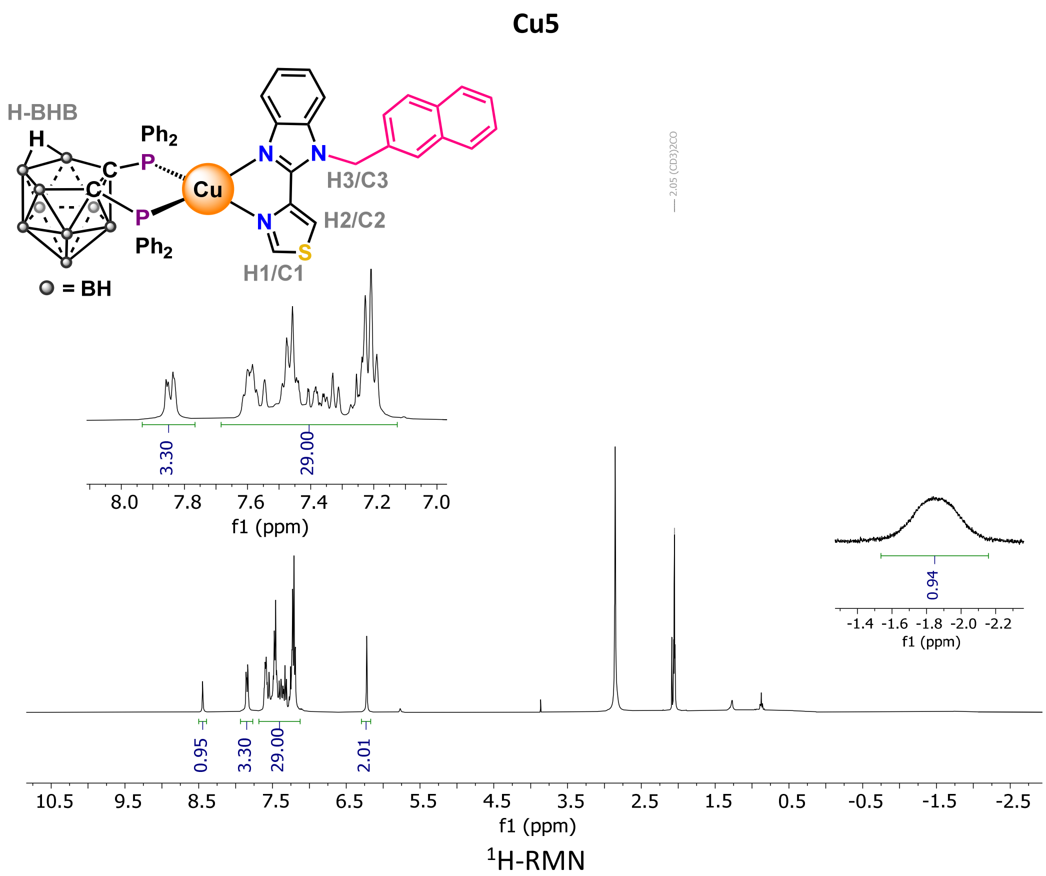

Figure S31. ^{1}H NMR spectrum of **Cu5**.

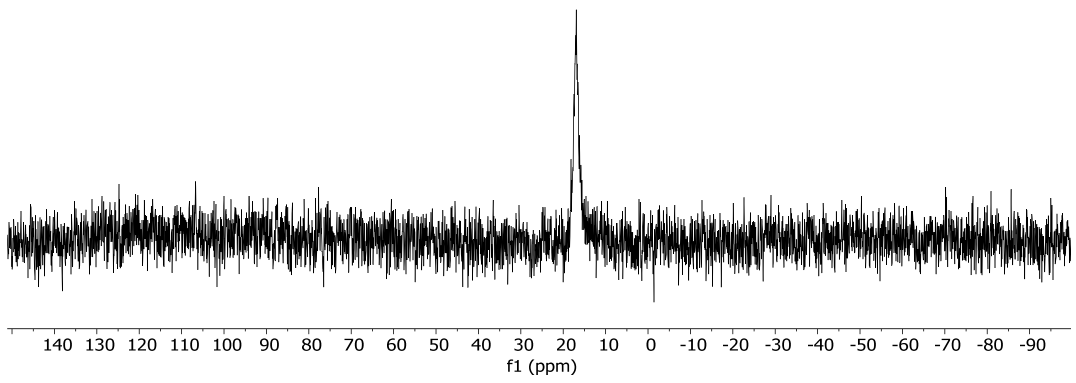

Figure S32. $^{31}P\{^{1}H\}$ NMR spectrum of **Cu5**.

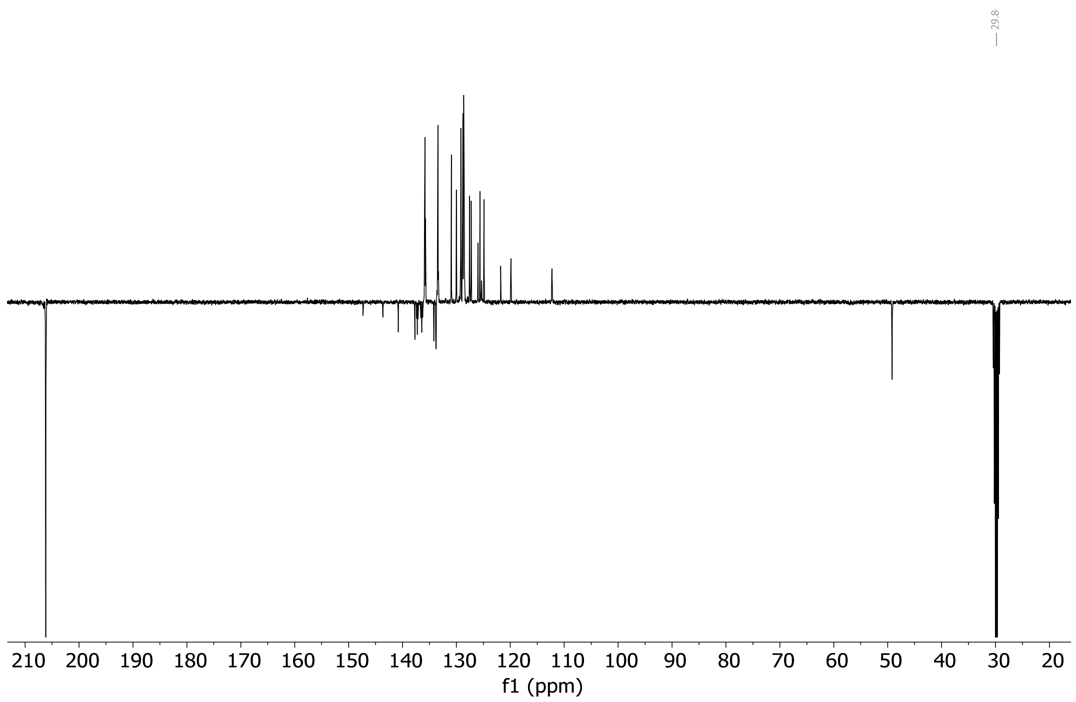

Figure S33. $^{13}C\{^{1}H\}$-APT NMR spectrum of **Cu5**.

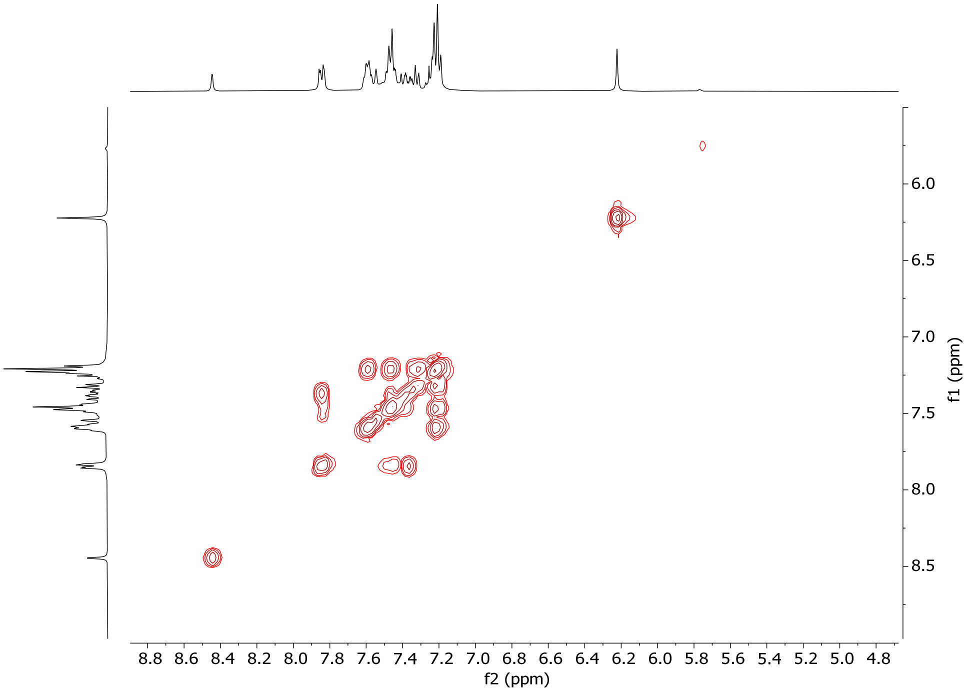

Figure S34. COSY ^{1}H-^{1}H NMR spectrum of **Cu5**.

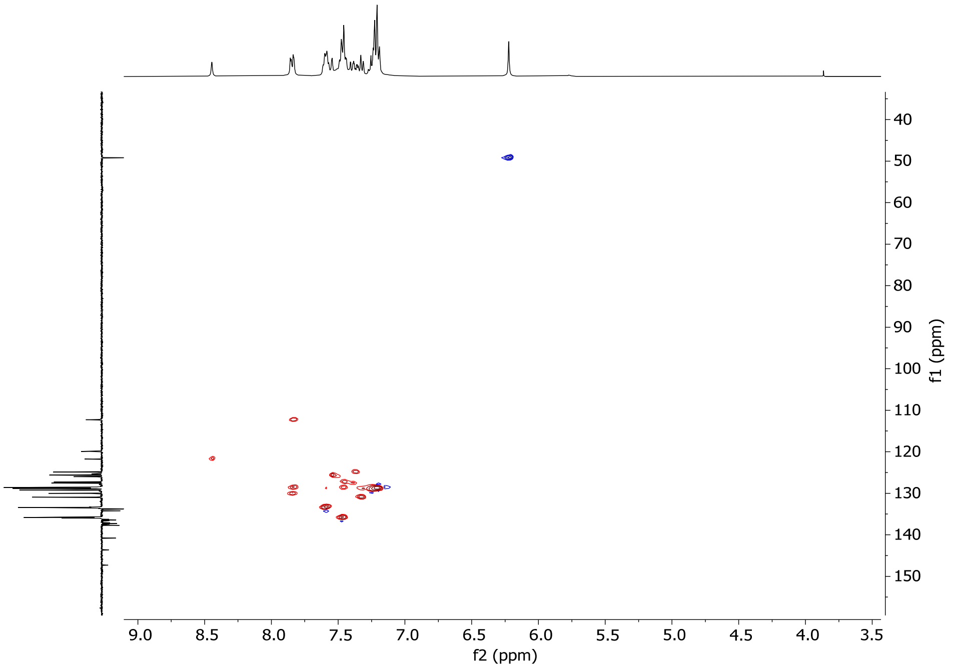

Figure S35. HSQC ^{1}H-$^{13}C\{^{1}H\}$ NMR spectrum of **Cu5**.

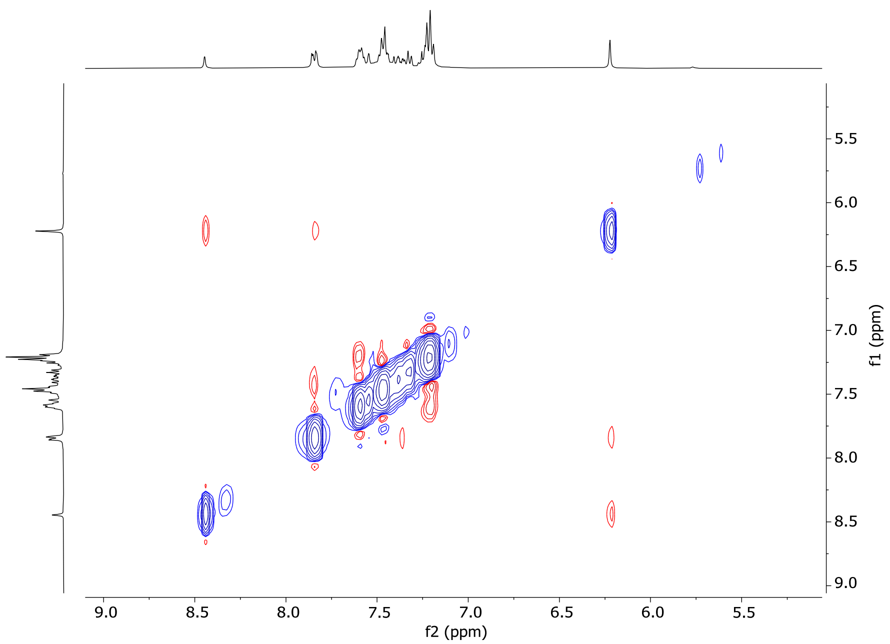

Figure S36. NOESY ^1H-^1H NMR spectrum of **Cu5**.

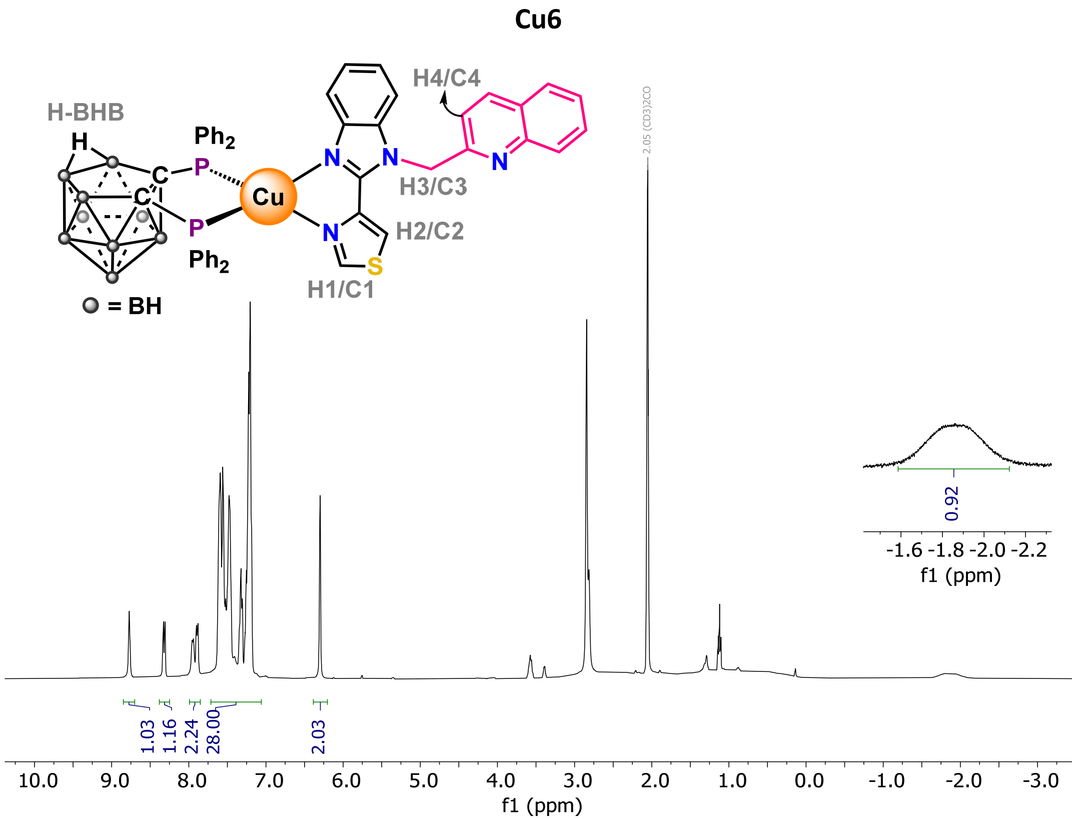

Figure S37. ^1H NMR spectrum of **Cu6**.

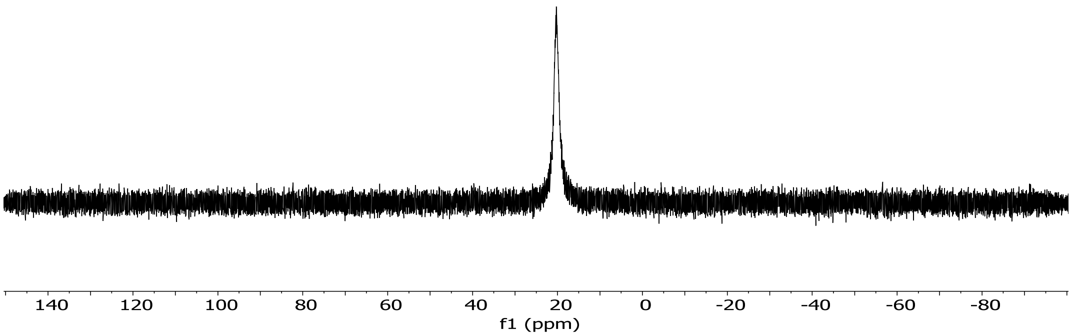

Figure S38. $^{31}P\{^{1}H\}$ NMR spectrum of **Cu6**.

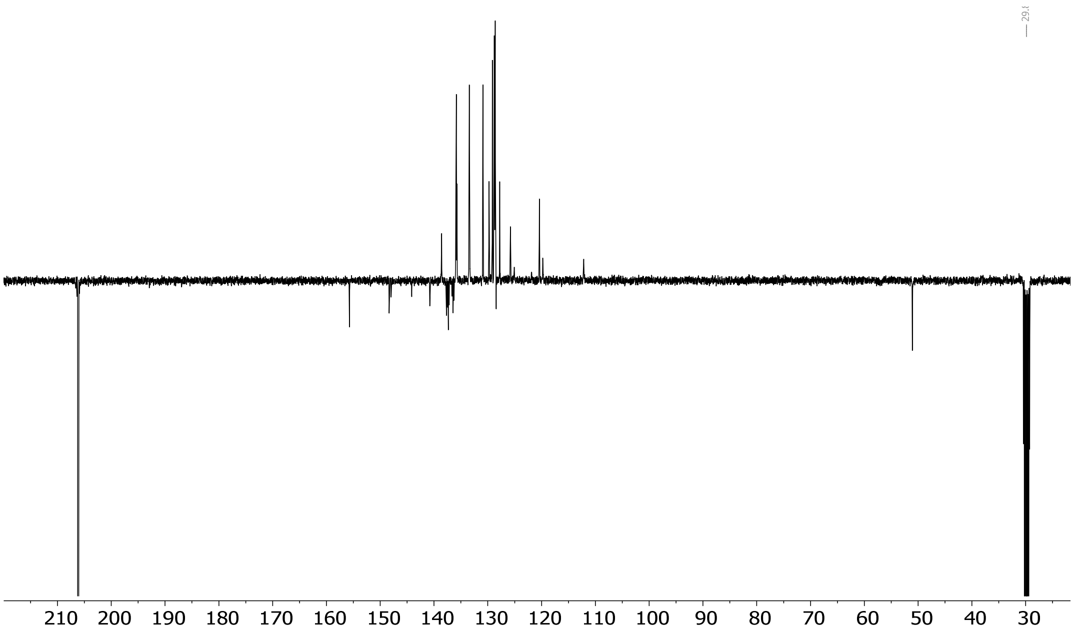

Figure S39. $^{13}C\{^{1}H\}$-APT NMR spectrum of **Cu6**.

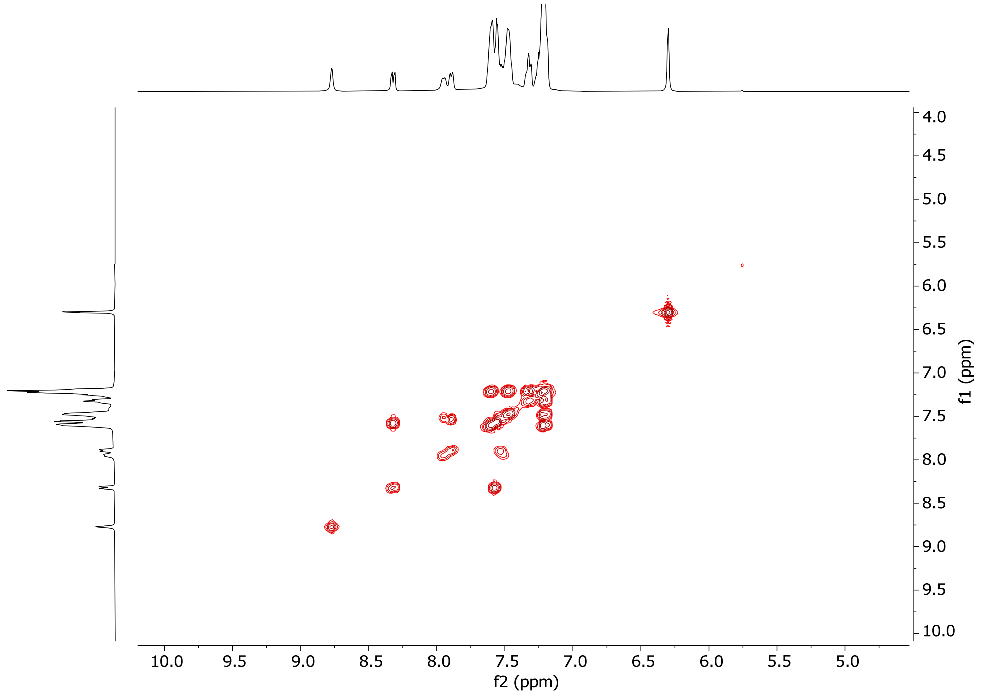

Figure S40. COSY ^{1}H-^{1}H NMR spectrum of **Cu6**.

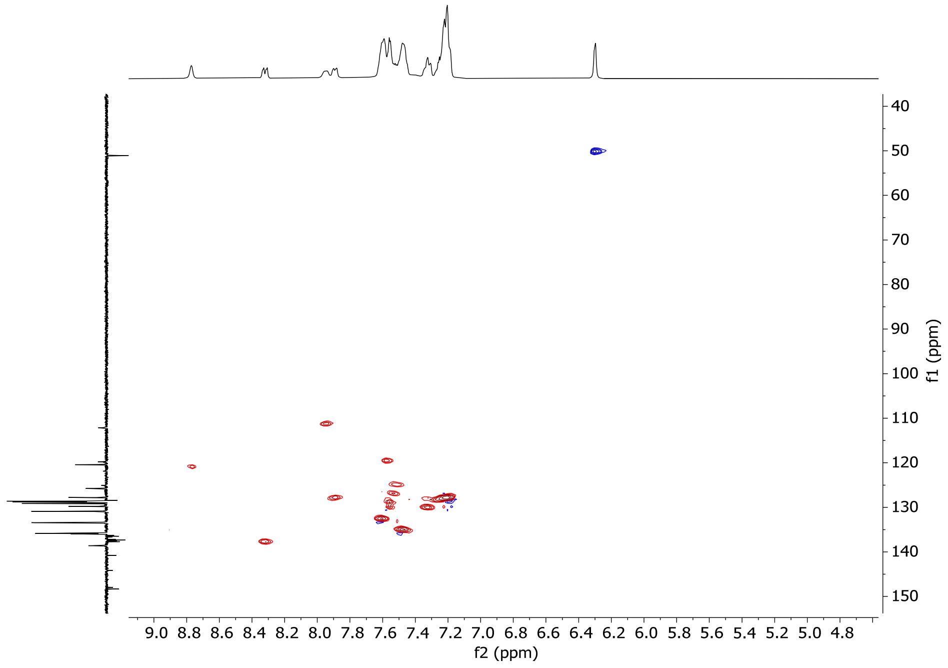

Figure S41. HSQC ^{1}H-$^{13}C\{^{1}H\}$ NMR spectrum of **Cu6**.

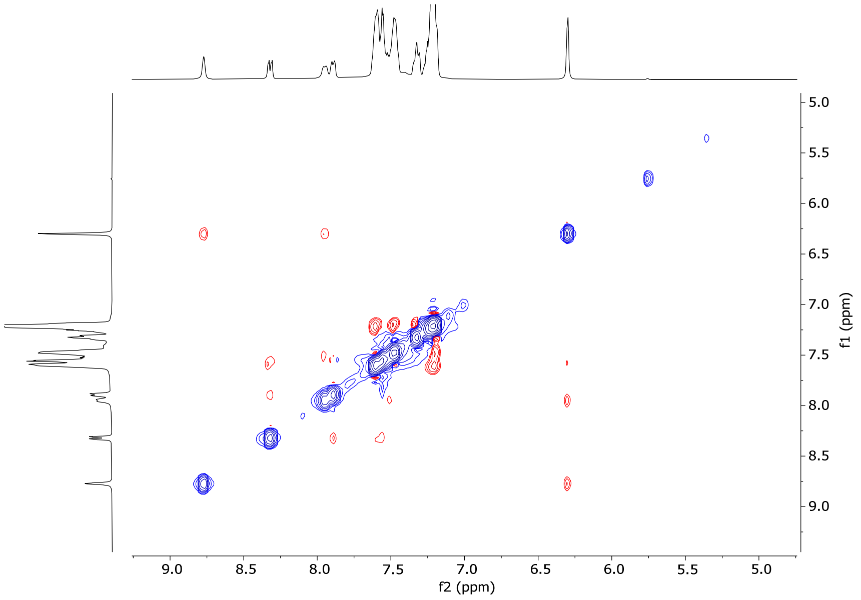

Figure S42. NOESY ^{1}H-^{1}H NMR spectrum of **Cu6**.

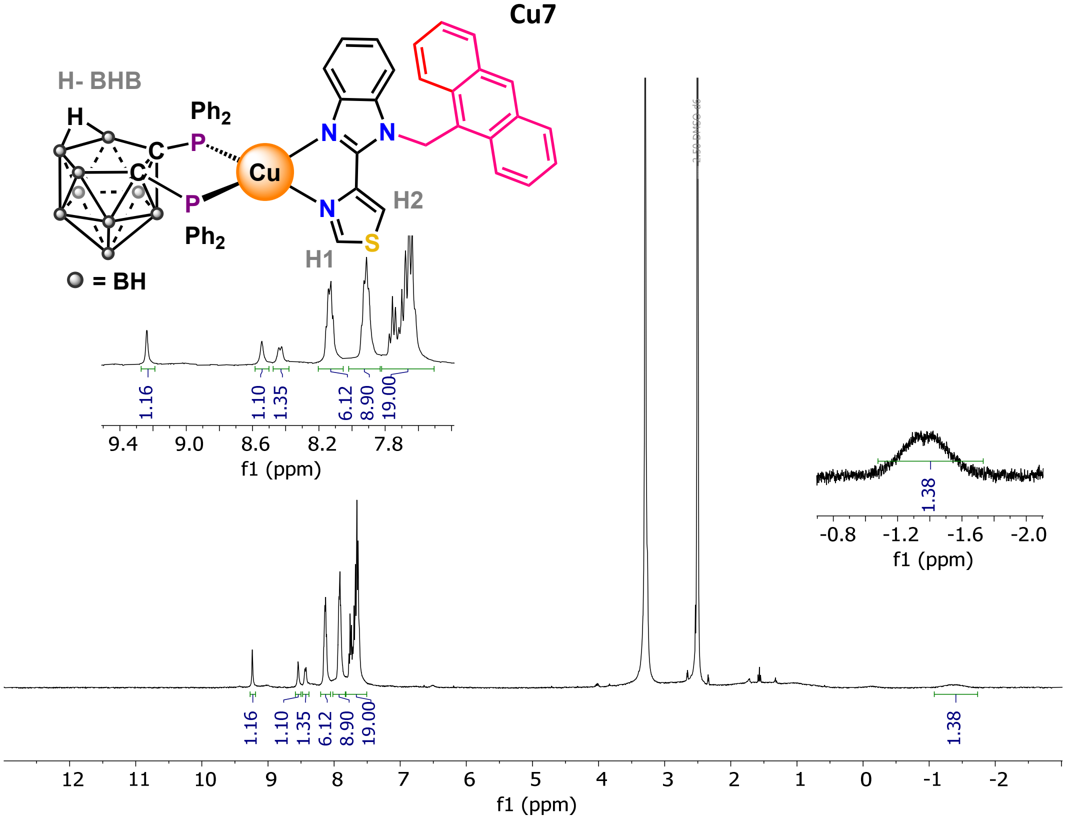

Figure S43. ^{1}H NMR spectrum of **Cu7**.

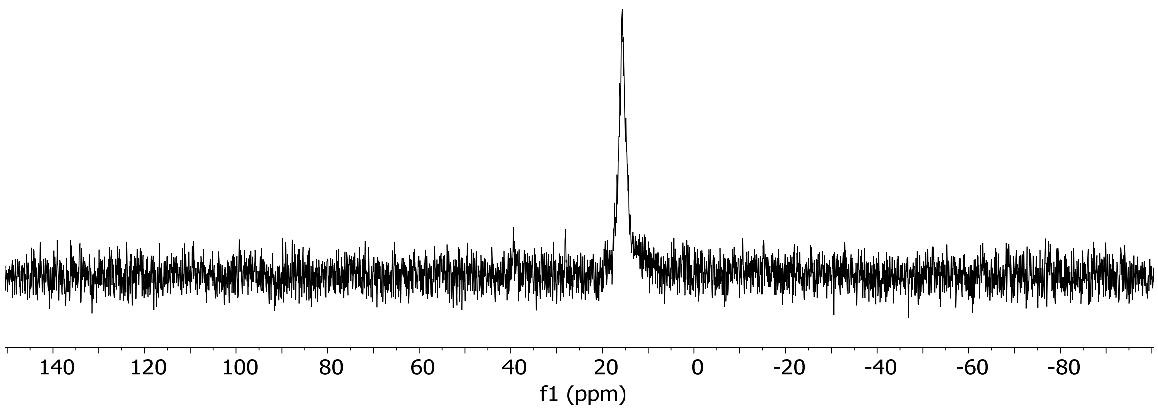

Figure S44. $^{31}P\{^{1}H\}$ NMR spectrum of **Cu7**.

Cu8

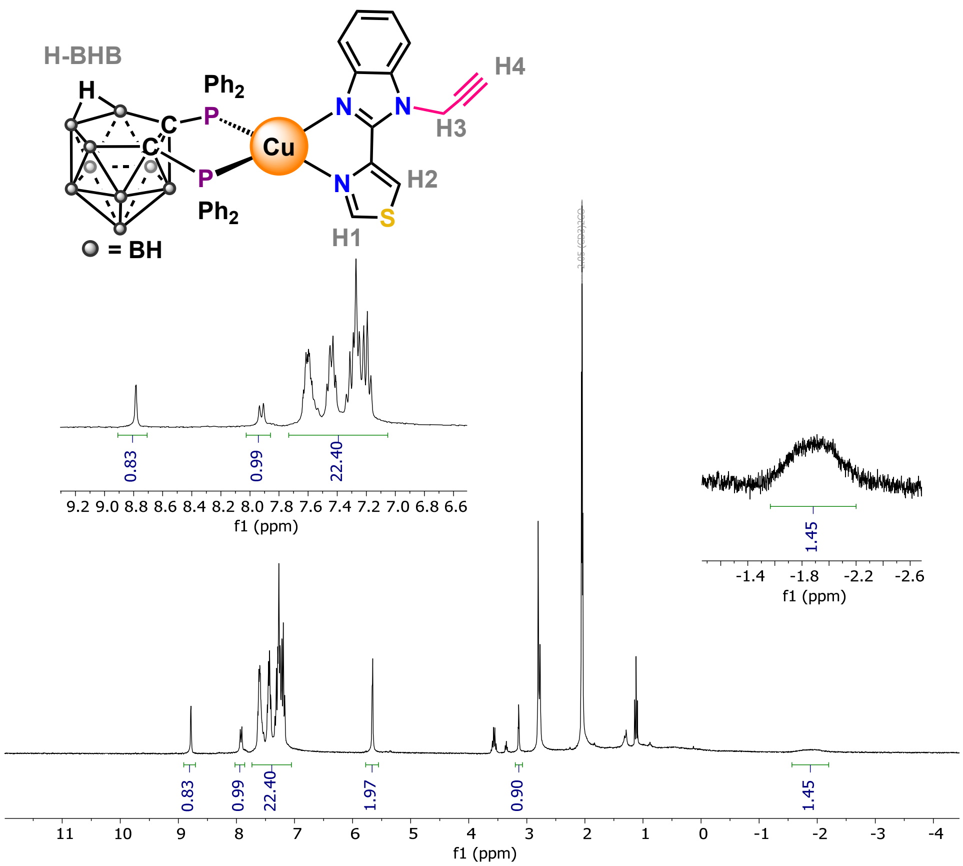

Figure S45. ^{1}H NMR spectrum of **Cu8**.

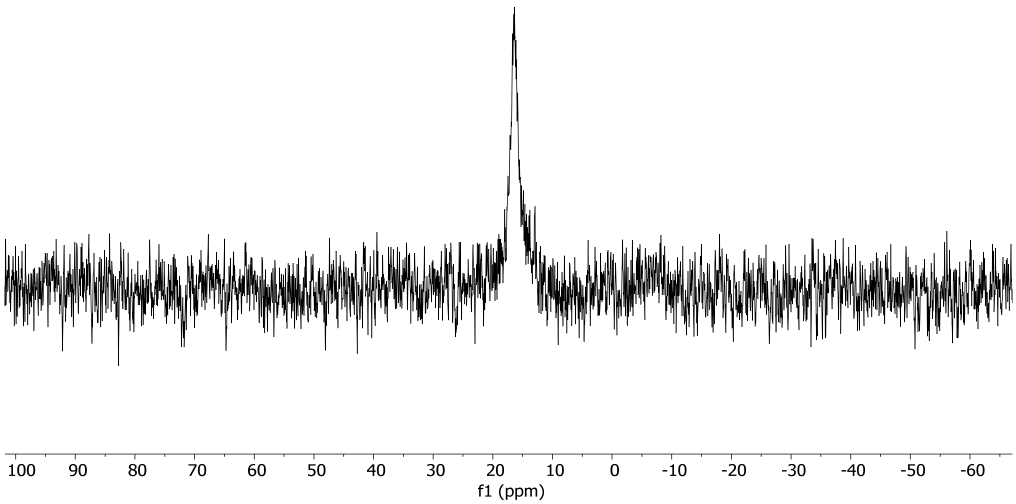

Figure S46. $^{31}P\{^{1}H\}$ NMR spectrum of **Cu8**.

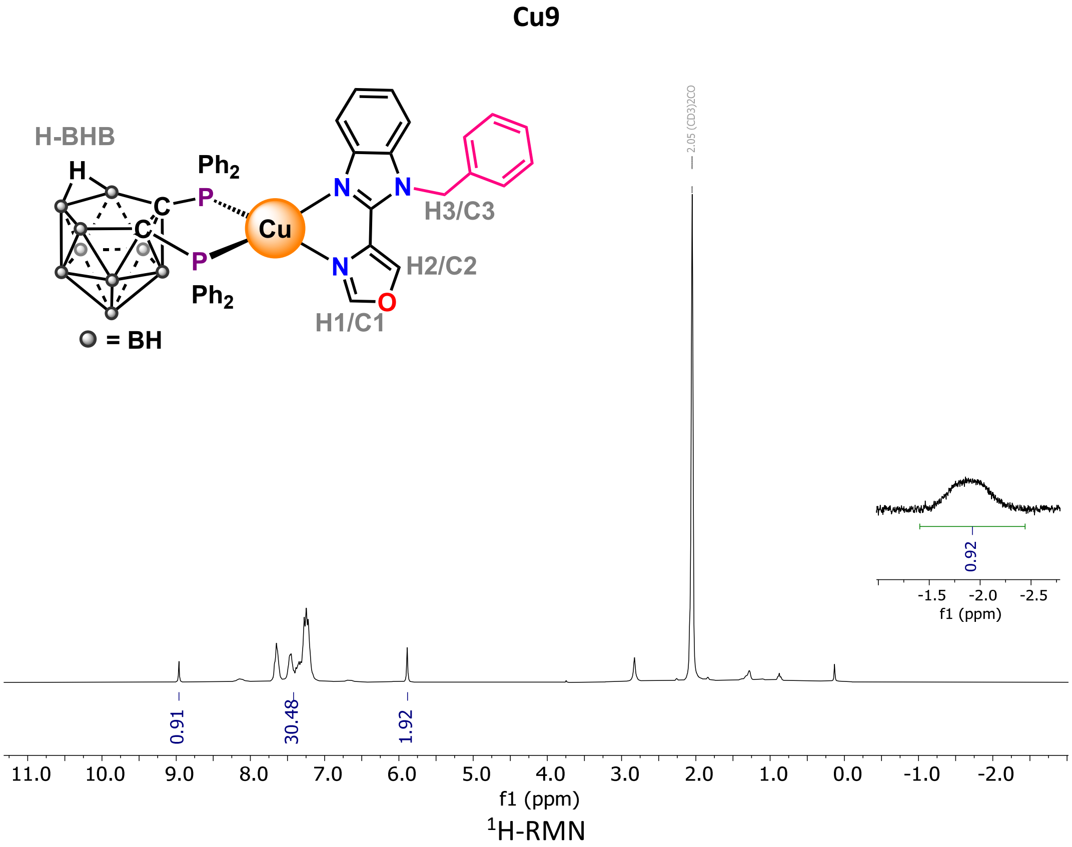

Figure S47. ^{1}H NMR spectrum of **Cu9**.

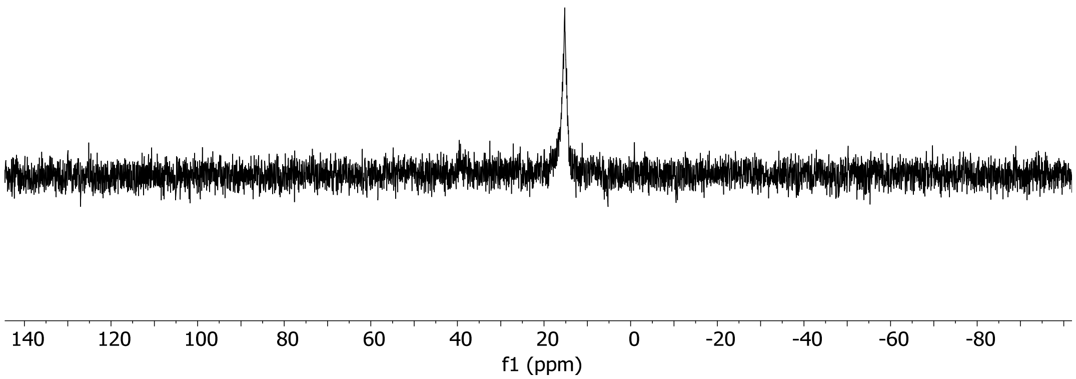

Figure S48. $^{31}P\{^{1}H\}$ NMR spectrum of **Cu9**.

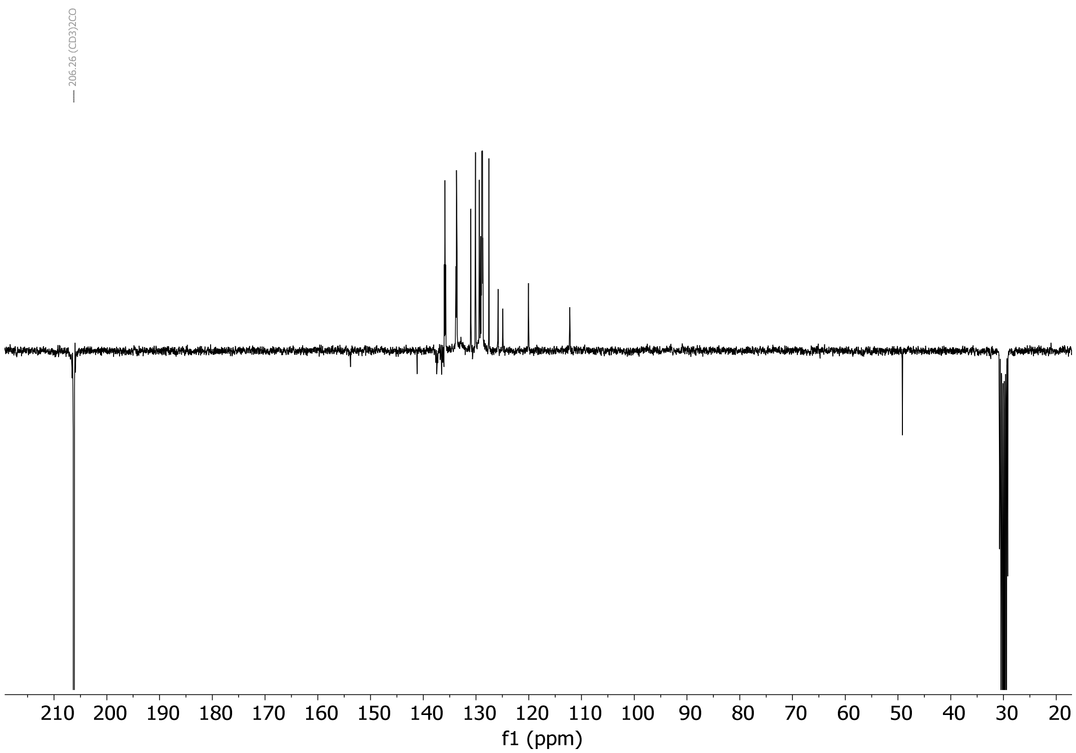

Figure S49. $^{13}C\{^{1}H\}$-APT NMR spectrum of **Cu9**.

Cu10

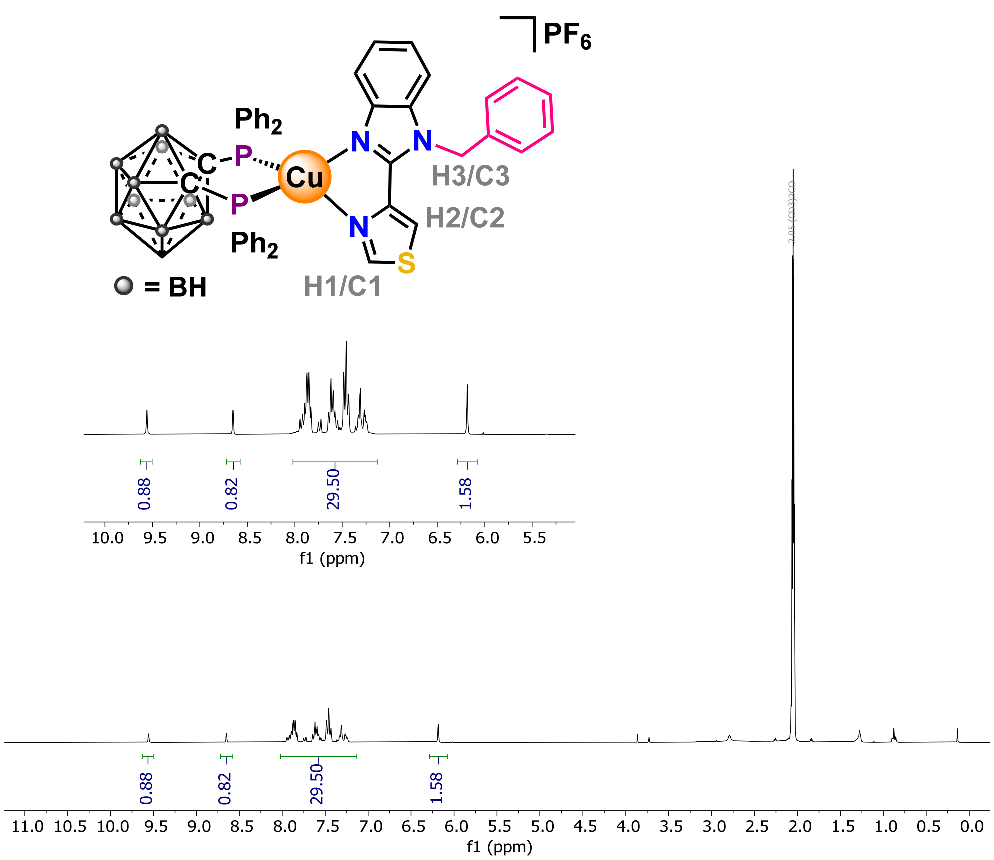

Figure S50. ^{1}H NMR spectrum of **Cu10**.

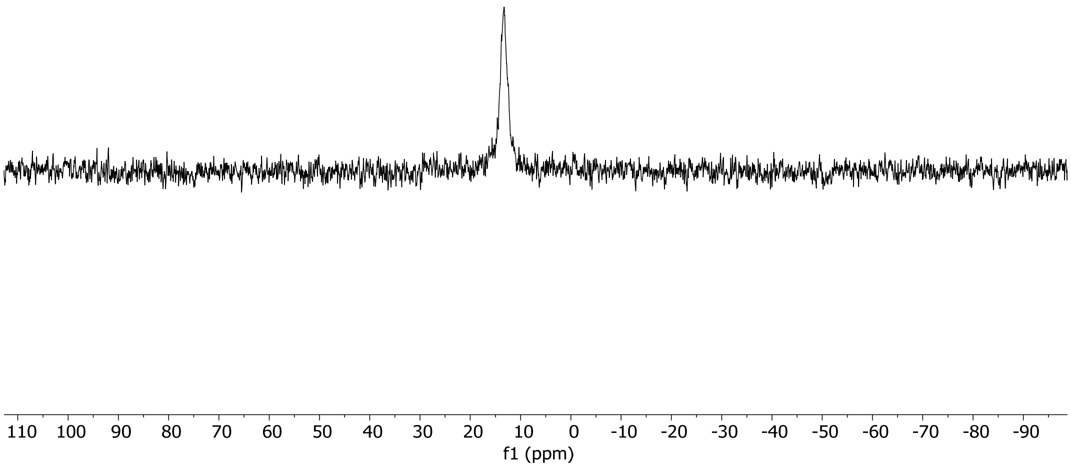

Figure S51. $^{31}P\{^{1}H\}$ NMR spectrum of **Cu10**.

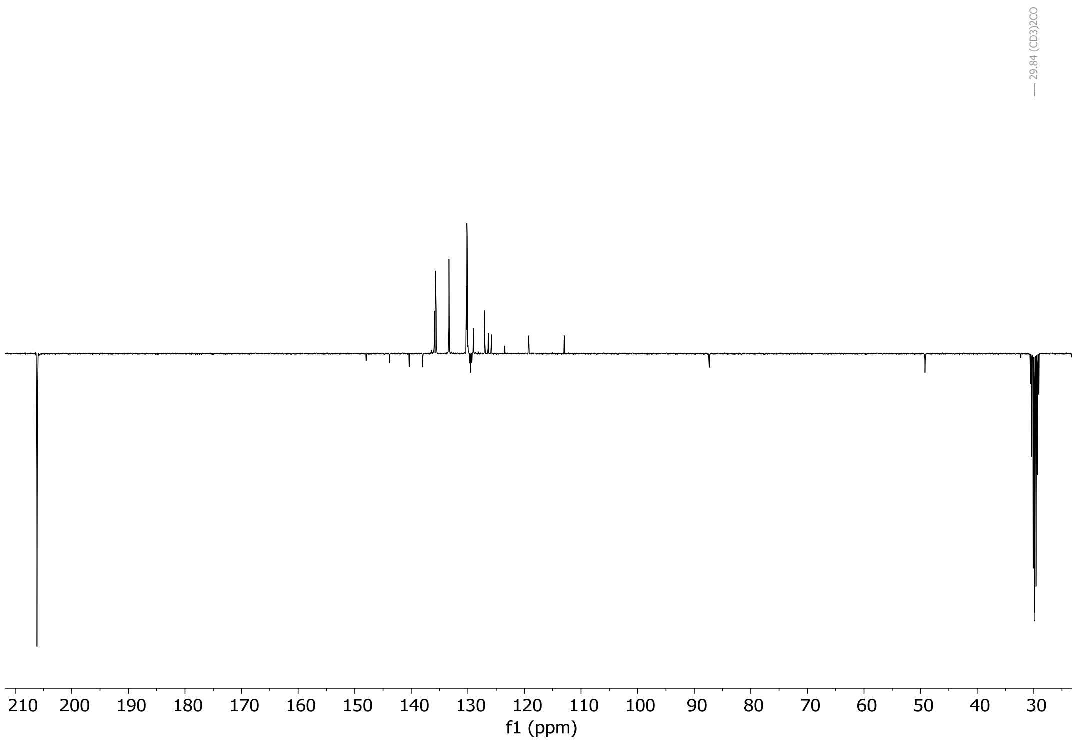

Figure S52. $^{13}C\{^{1}H\}$-APT NMR spectrum of **Cu10**.

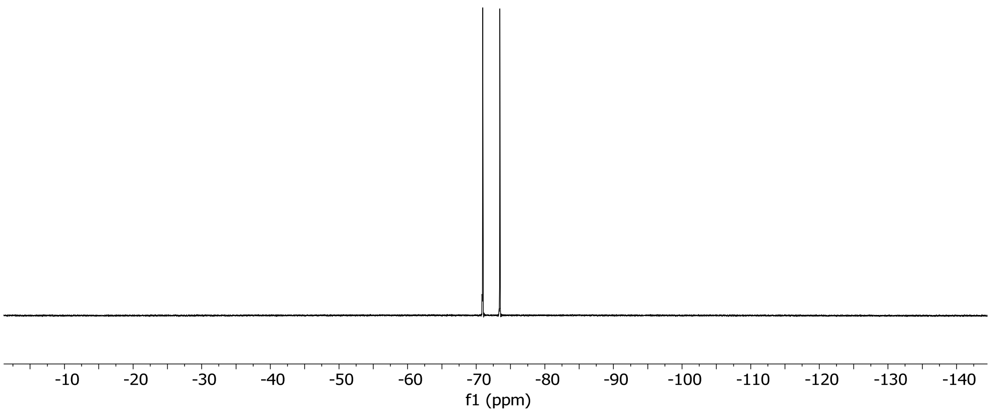

Figure S53. ^{19}F NMR spectrum of **Cu10**.

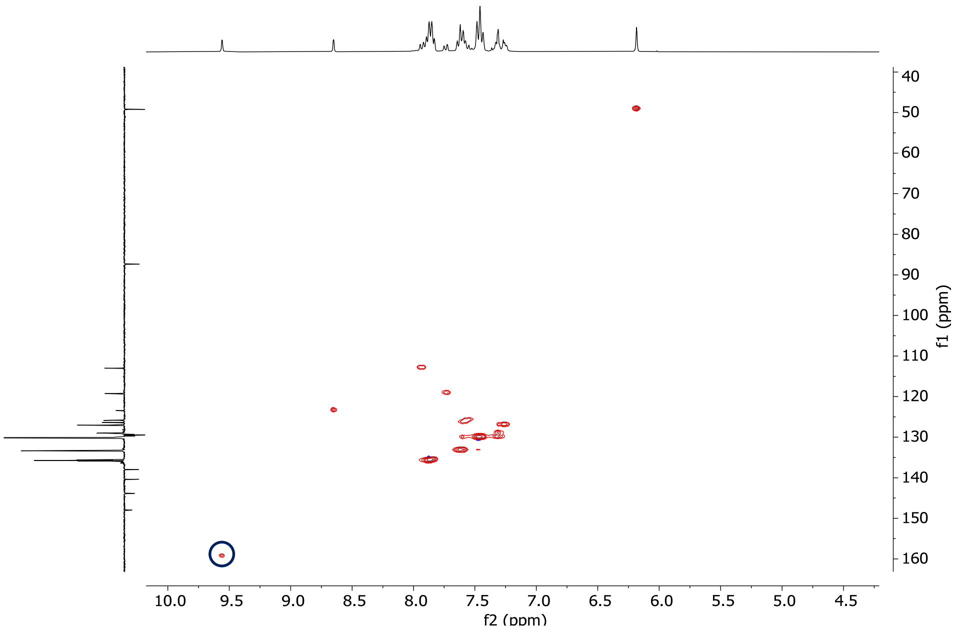

Figure S54. HSQC ^{1}H-$^{13}C\{^{1}H\}$ NMR spectrum of **Cu10**.

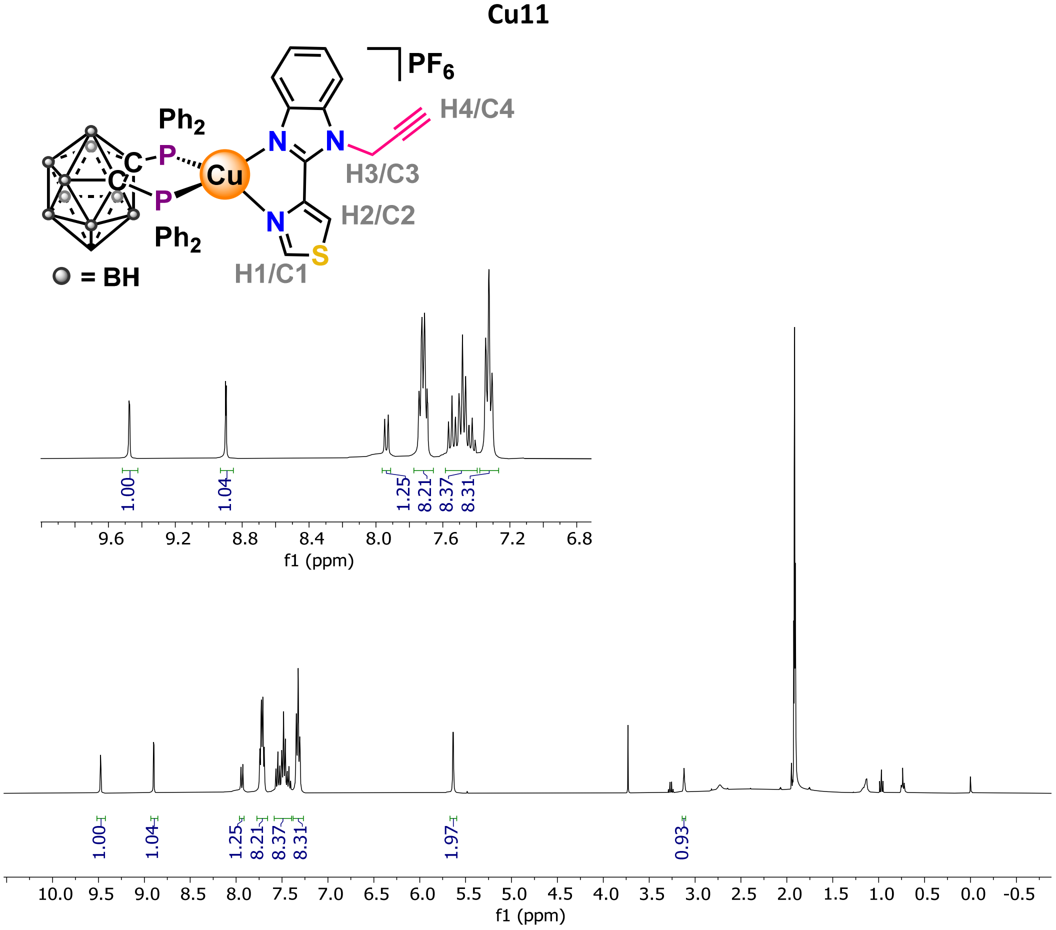

Figure S55. ^{1}H NMR spectrum of **Cu11**.

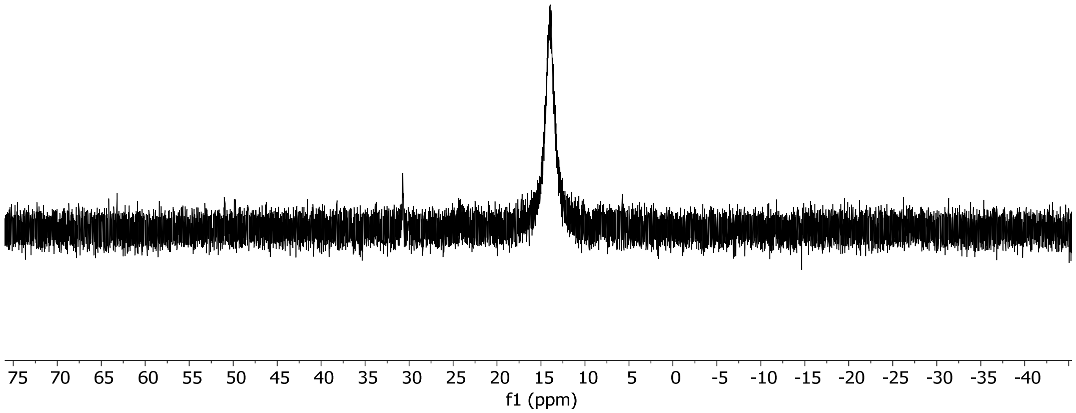

Figure S56. $^{31}P\{^1H\}$ NMR spectrum of **Cu11**.

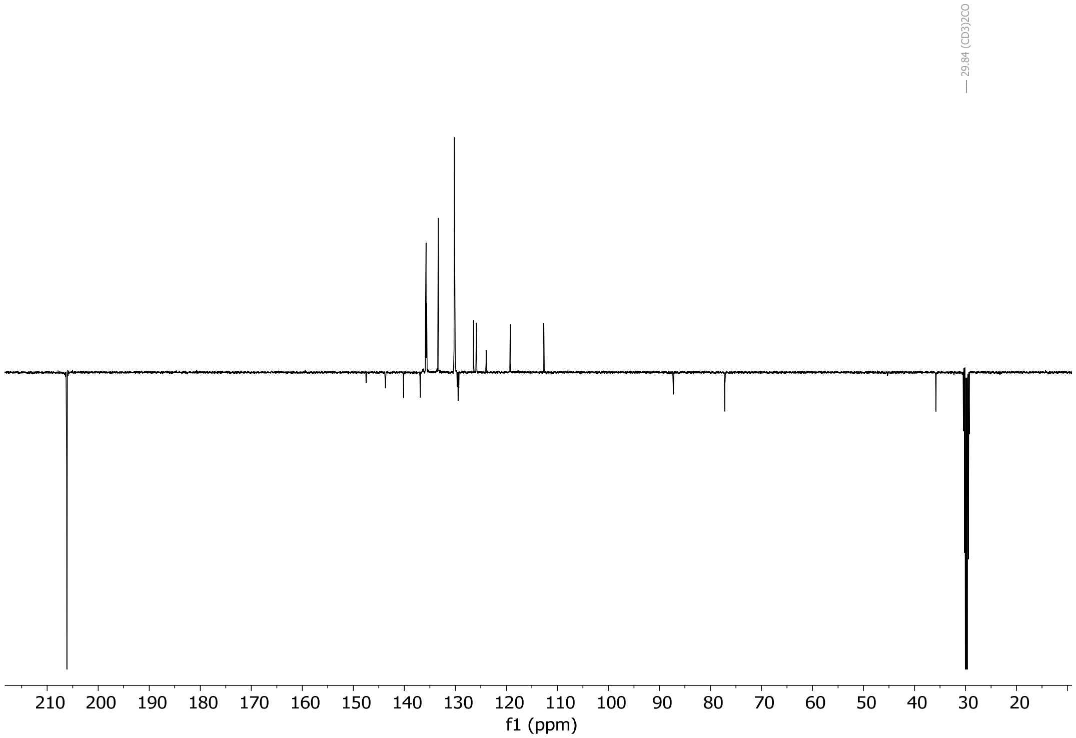

Figure S57. $^{13}C\{^1H\}$-APT NMR spectrum of **Cu11**.

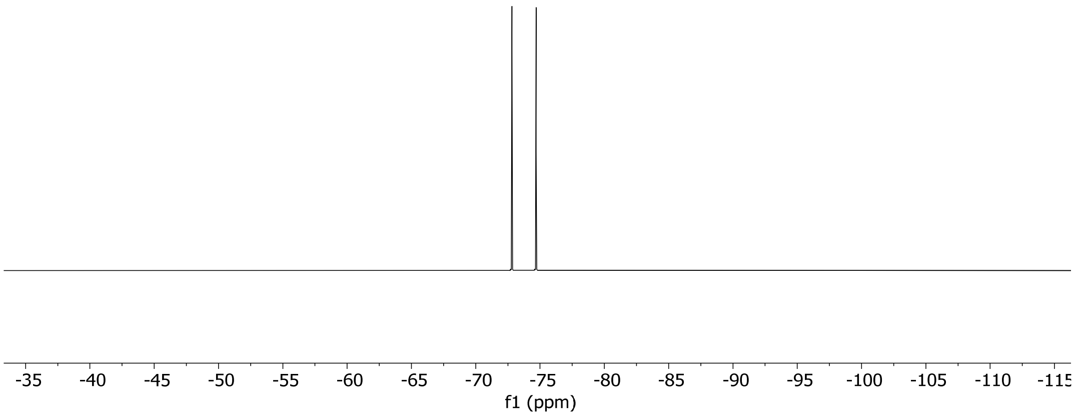

Figure S58. ^{19}F NMR spectrum of **Cu11**.

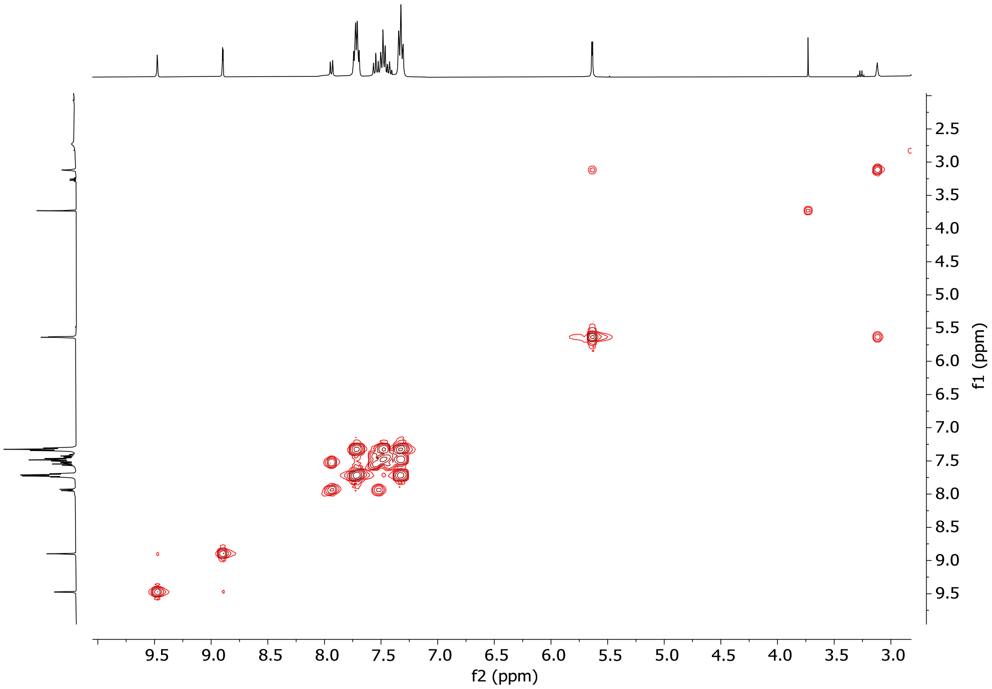

Figure S59. COSY ^{1}H-^{1}H NMR spectrum of **Cu11**.

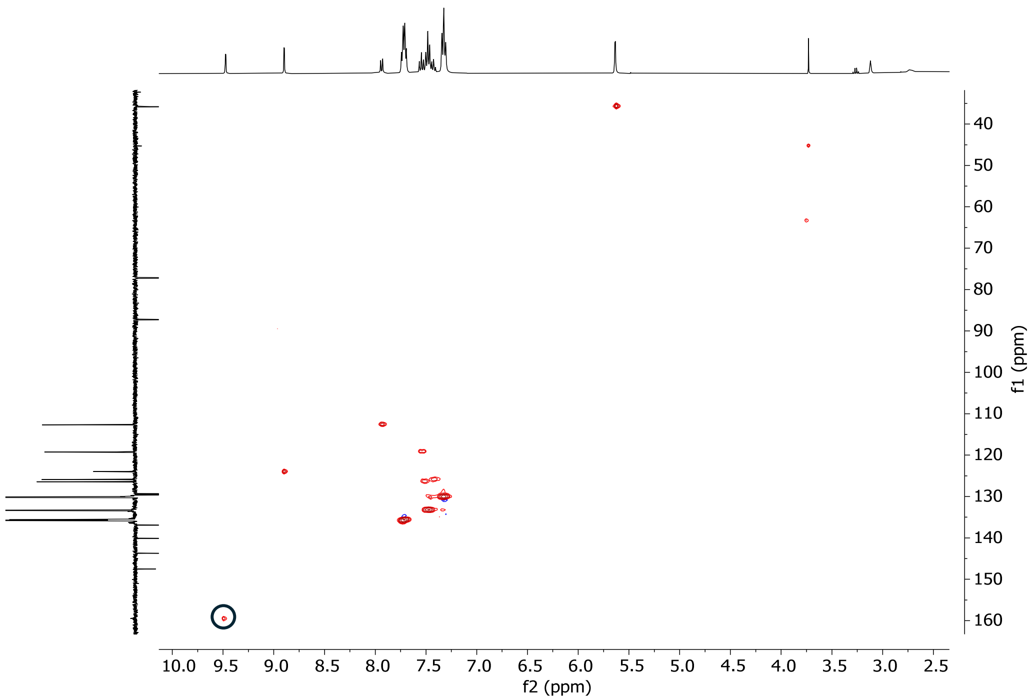

Figure S60. HSQC ^{1}H-^{13}C NMR spectrum of **Cu11**.

S3.- Mass Spectra

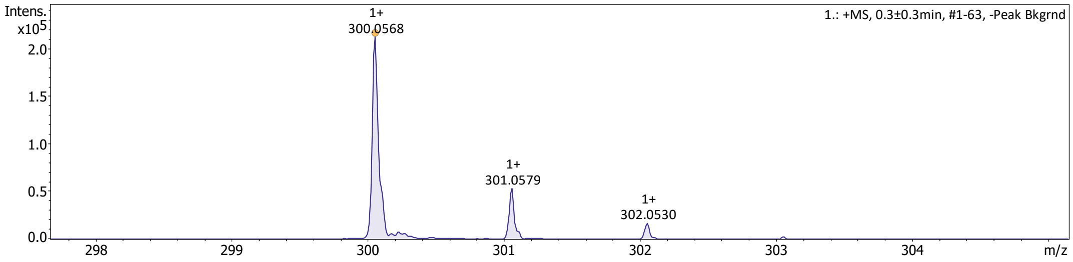

Figure S61. HRMS-ESI(+) of **L2**.

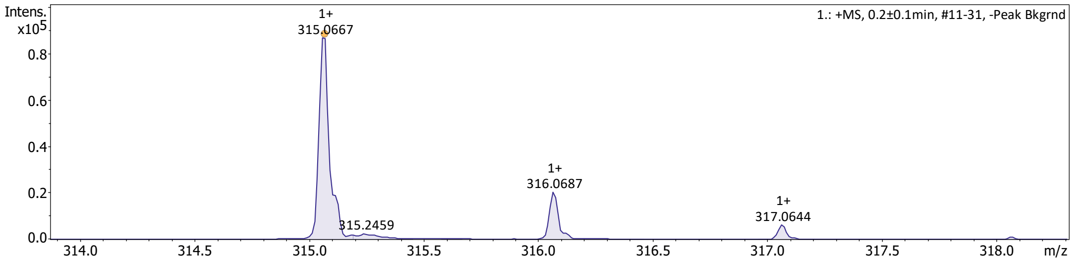

Figure S62. HRMS-ESI(+) of **L4**.

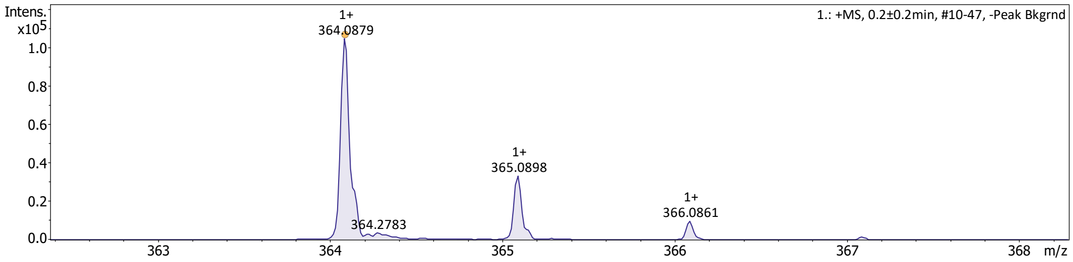

Figure S63. HRMS-ESI(+) of **L5**.

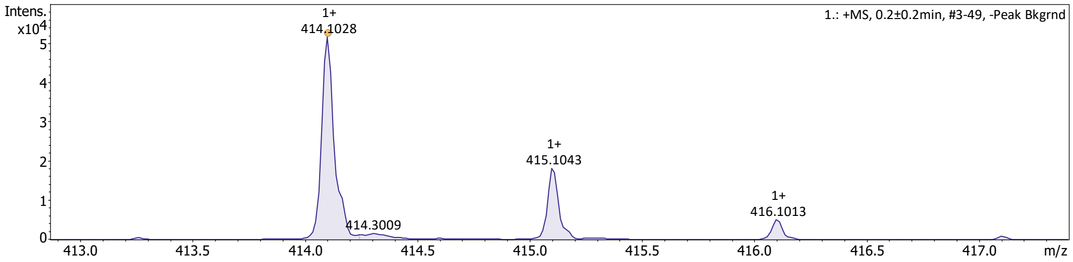

Figure S64. HRMS-ESI(+) of **L7**.

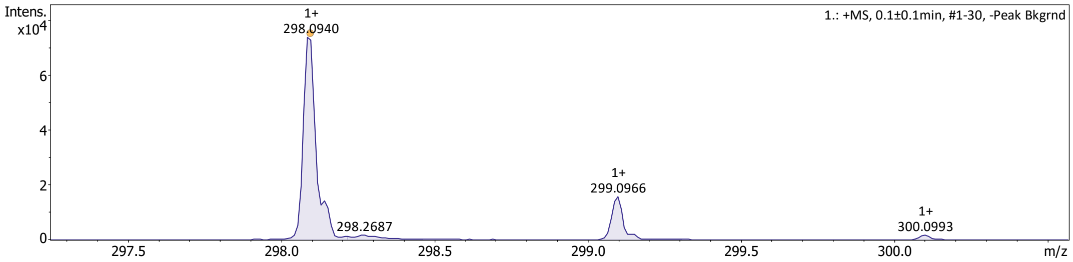

Figure S65. HRMS-ESI(+) of **L9**.

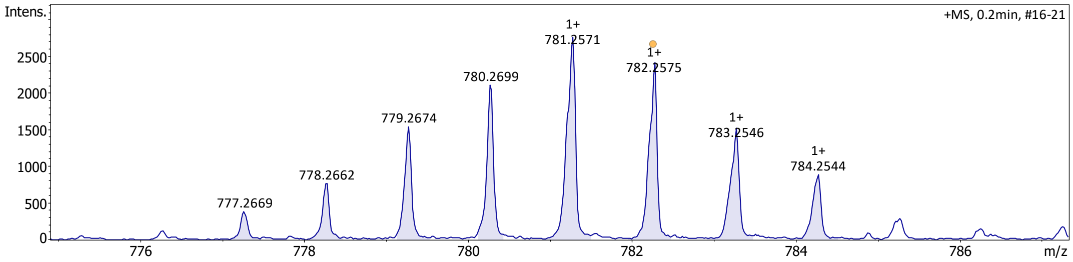

Figure S66. HRMS-ESI(+) of **Cu1**.

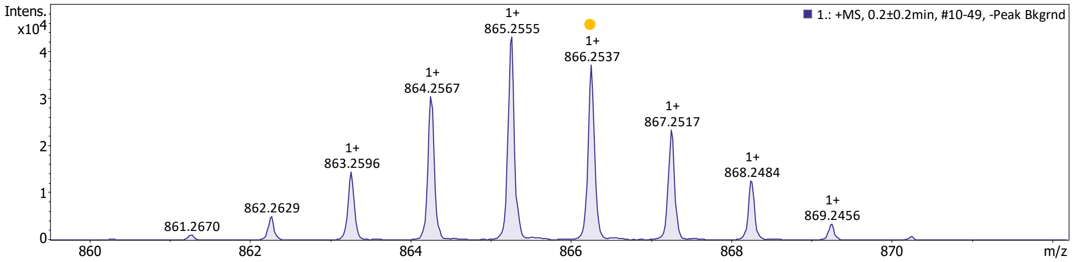

Figure S67. HRMS-ESI(+) of **Cu2**.

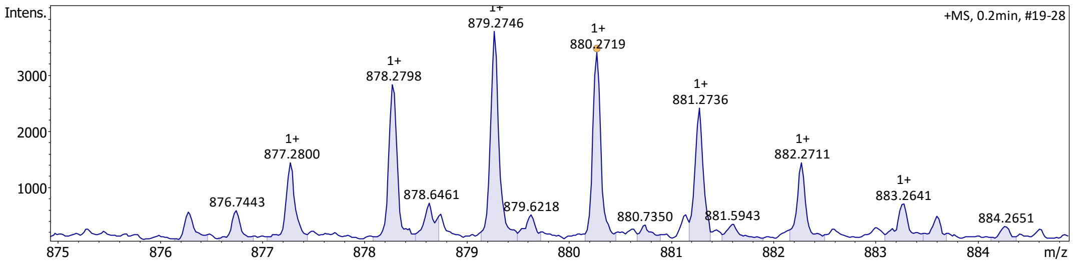

Figure S68. HRMS-ESI(+) of **Cu3**.

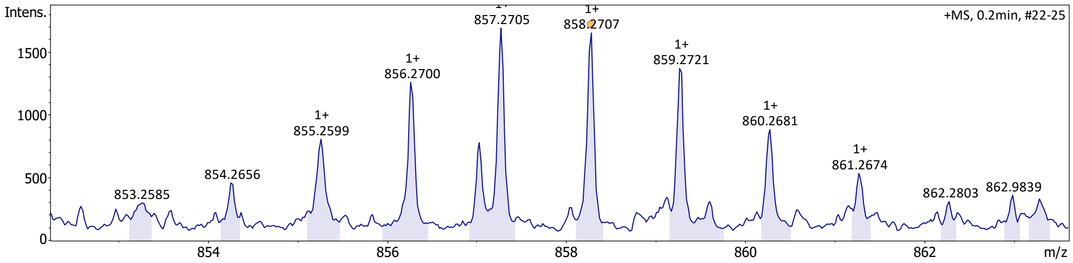

Figure S69. HRMS-ESI(+) of **Cu4**.

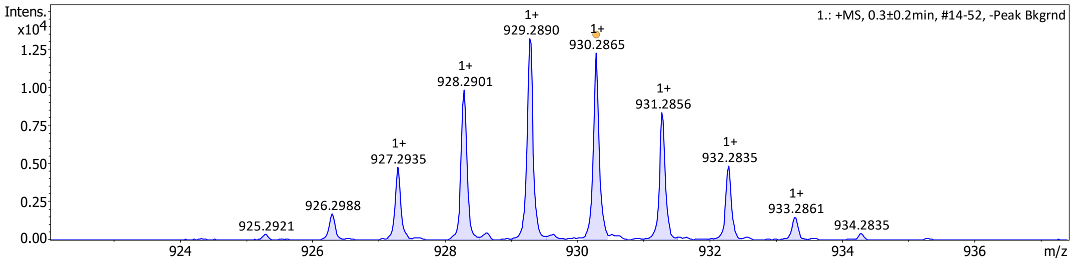

Figure S70. HRMS-ESI(+) of **Cu5**.

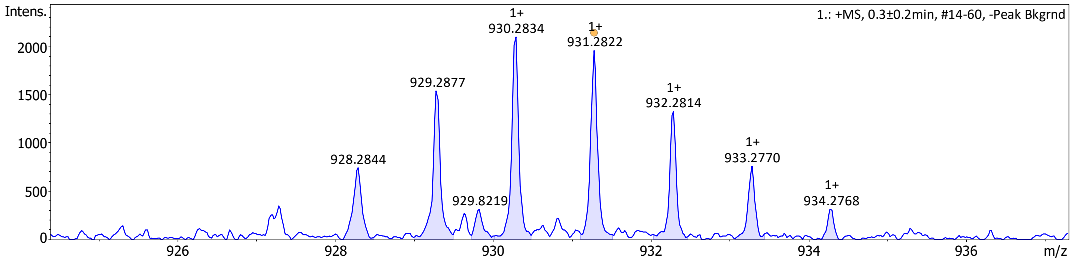

Figure S71. HRMS-ESI(+) of **Cu6**.

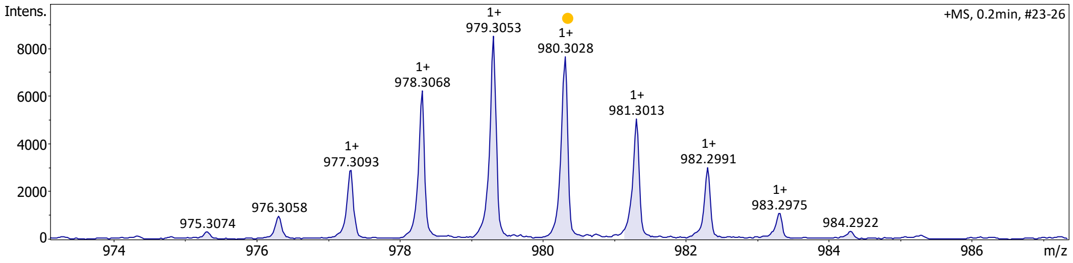

Figure S72. HRMS-ESI(+) of **Cu7**.

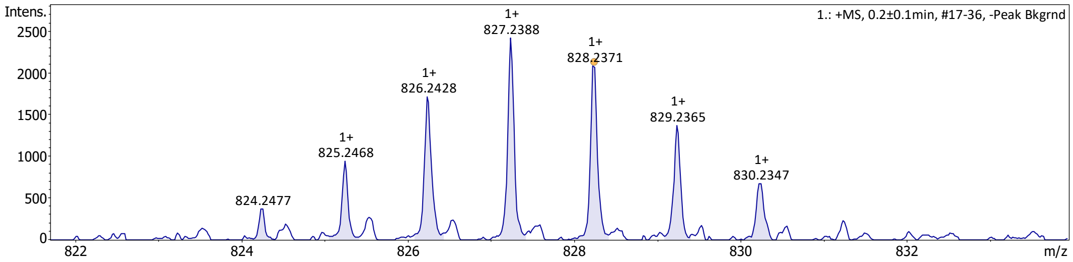

Figure S73. HRMS-ESI(+) of **Cu8**.

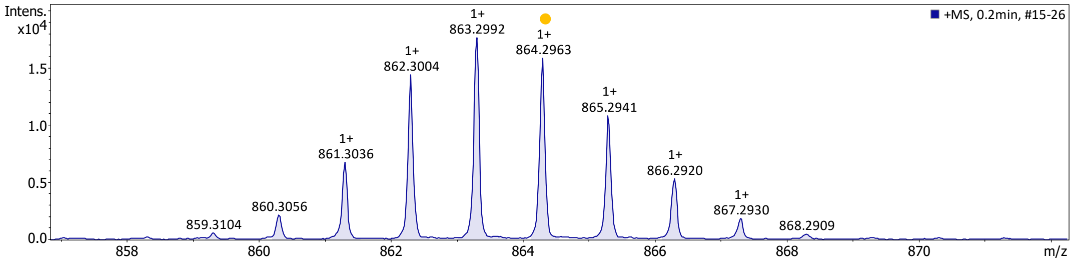

Figure S74. HRMS-ESI(+) of **Cu9**.

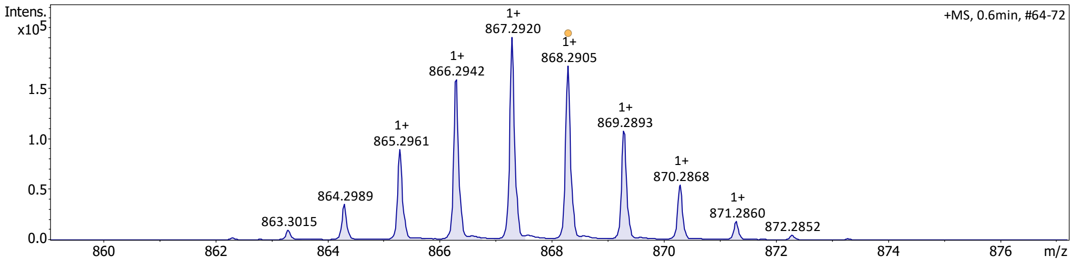

Figure S75. HRMS-ESI(+) of **Cu10**.

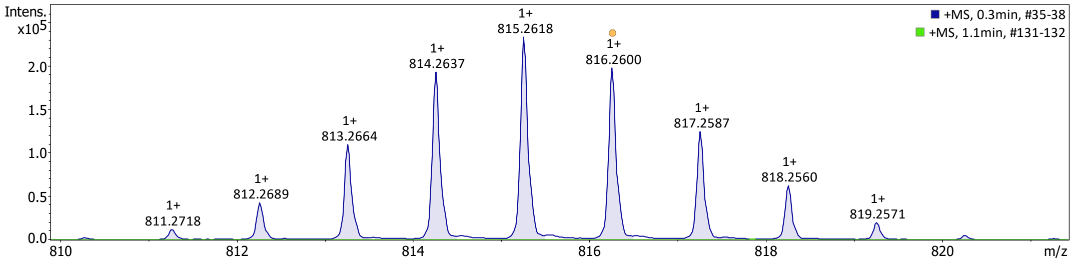

Figure S76. HRMS-ESI(+) of **Cu11**.

S4.- Emission and excitation spectra

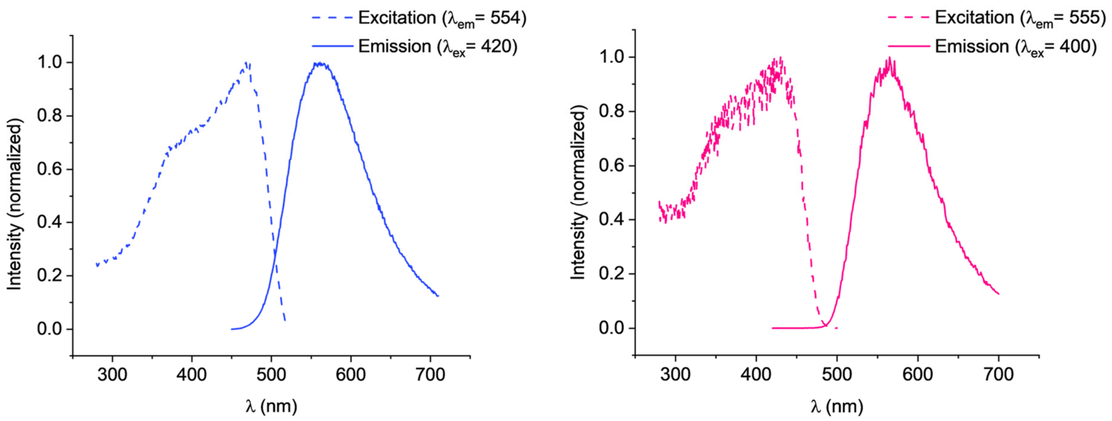

Figure S77. Excitation and emission spectra at rt (left) and 77 K (right) of **Cu1** in the solid state.

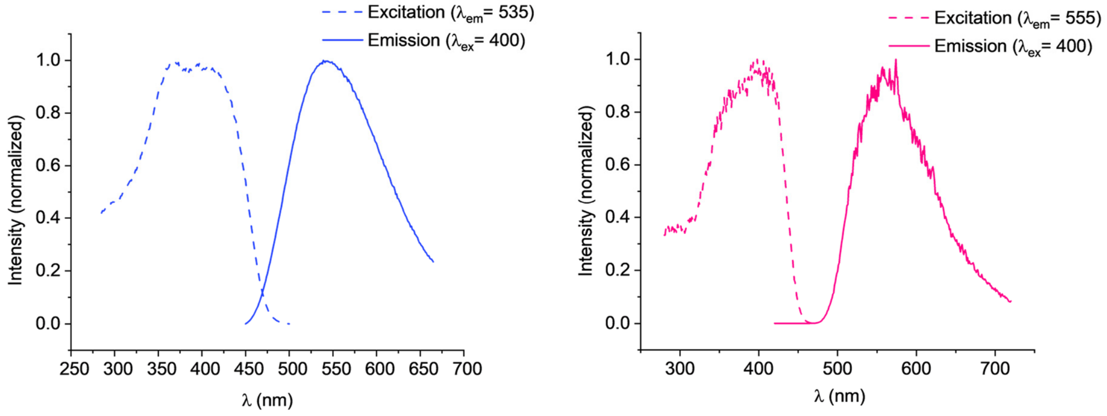

Figure S78. Excitation and emission spectra at rt (left) and 77 K (right) of **Cu2** in the solid state.

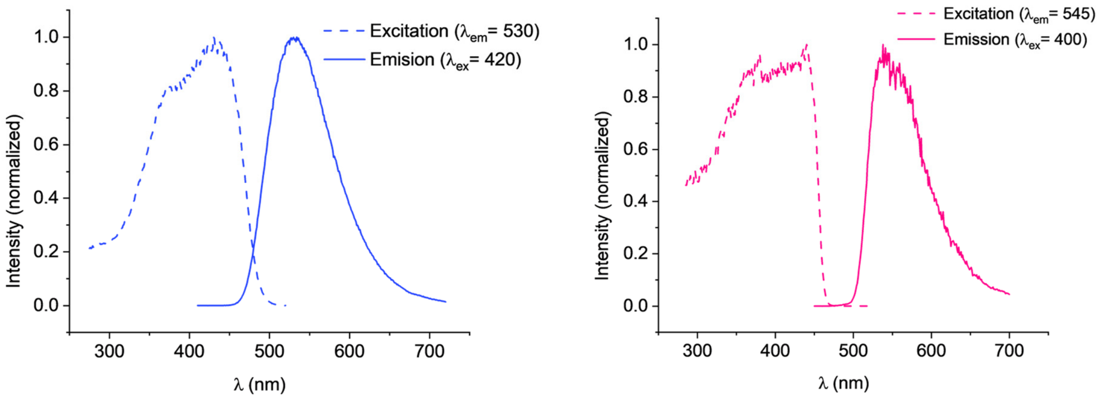

Figure S79. Excitation and emission spectra at rt (left) and 77 K (right) of **Cu3** in the solid state.

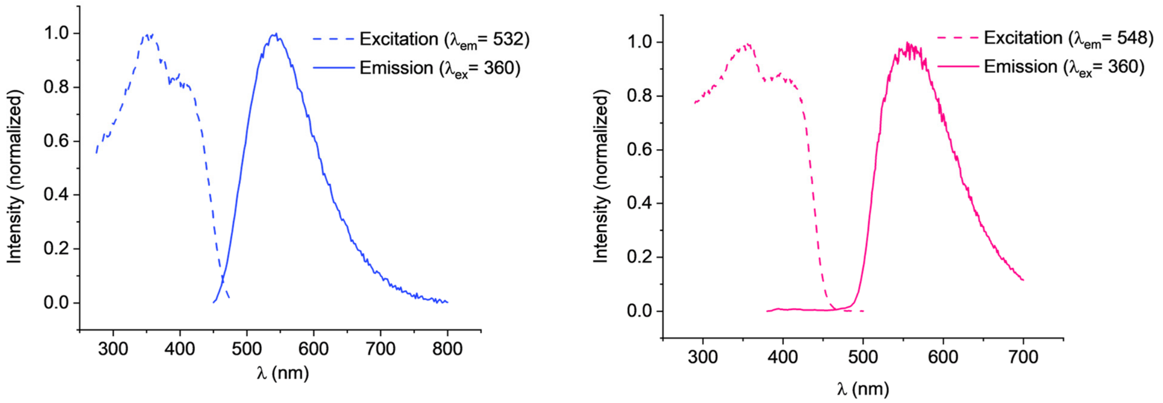

Figure S80. Excitation and emission spectra at rt (left) and 77 K (right) of **Cu4** in the solid state.

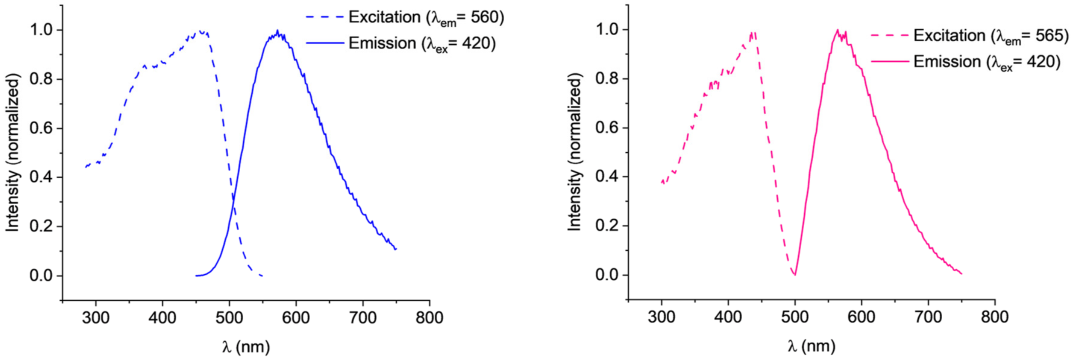

Figure S81. Excitation and emission spectra at rt (left) and 77 K (right) of **Cu5** in the solid state.

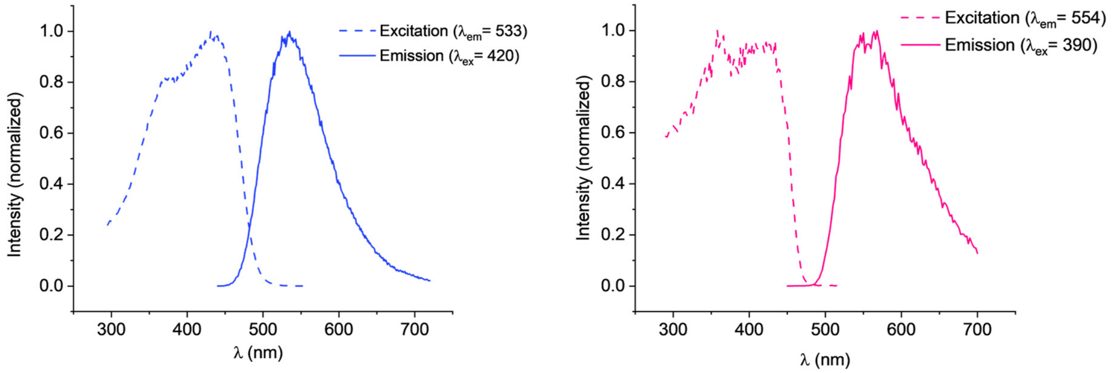

Figure S82. Excitation and emission spectra at rt (left) and 77 K (right) of **Cu6** in the solid state.

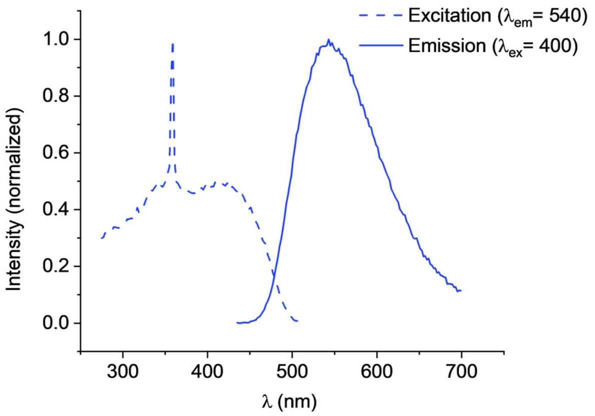

Figure S83. Excitation and emission spectra at rt of **Cu7** in the solid state**.**

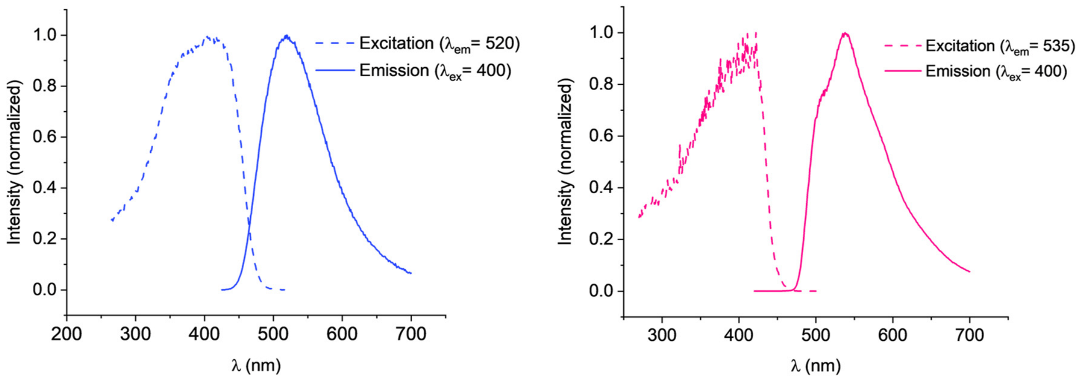

Figure S84. Excitation and emission spectra at rt (left) and 77 K (right) of **Cu8** in the solid state.

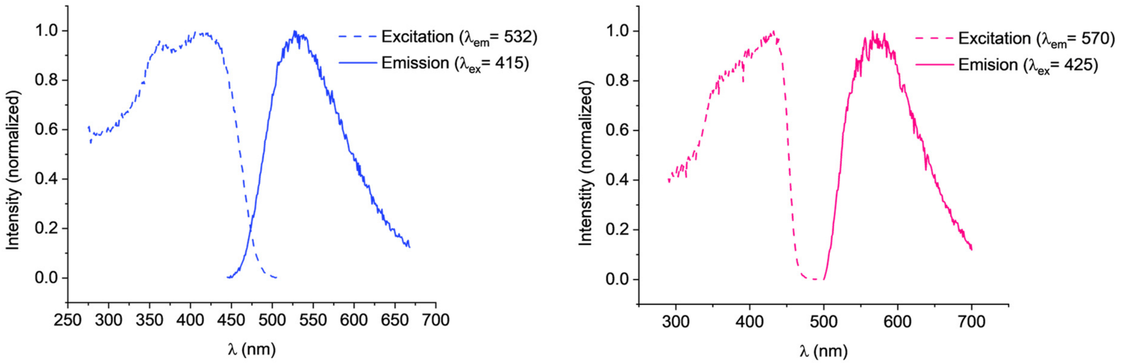

Figure S85. Excitation and emission spectra at rt (left) and 77 K (right) of **Cu9** in the solid state.

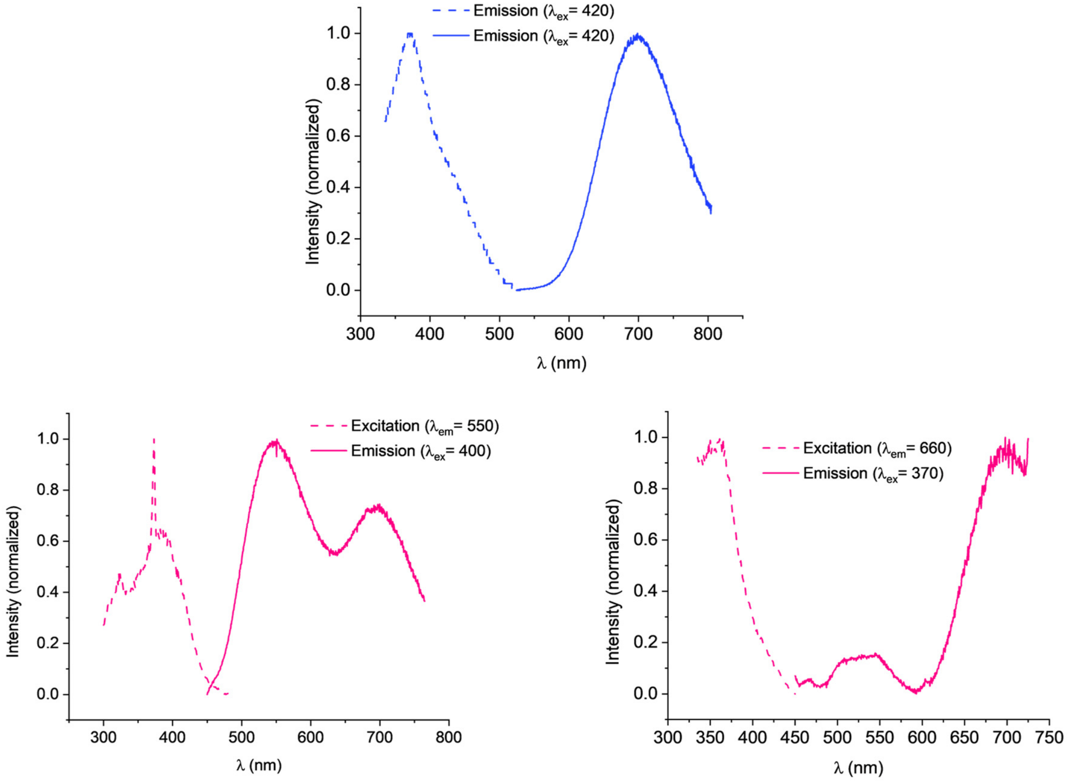

Figure S86. Excitation and emission spectra at rt (up) and 77 K (bottom) of **Cu10** in the solid state.

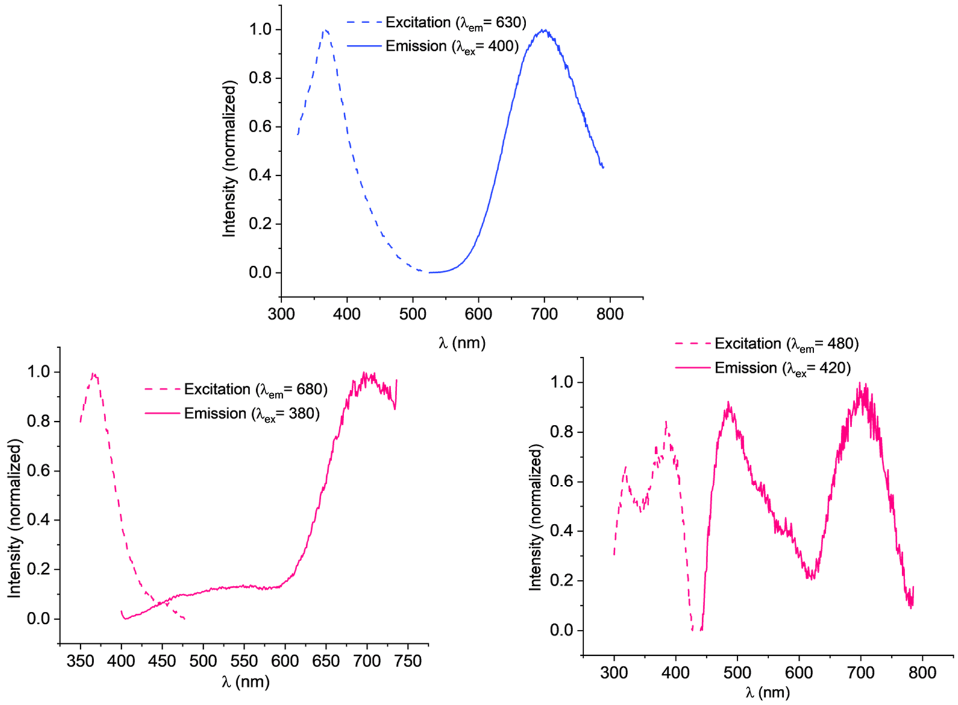

Figure S87. Excitation and emission spectra at rt (up) and 77 K (bottom) of **Cu11** in the solid state.

S5.- Lifetime fitting curves

Cu1

Rt

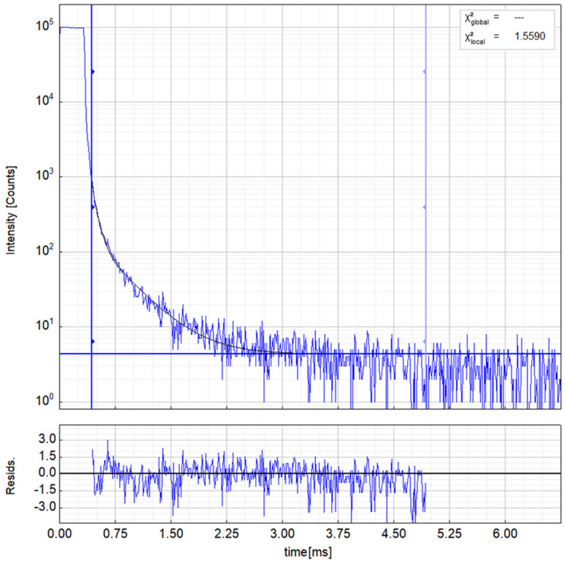

Measurement Context: Decay

Excitation: U pol 405±15nm with PLS-400

Detection: U pol 552±8nm 100000 peak counts

grating 1200/500+

detector UV-red [PMT]

Parameter	Value	Δ	δ
A_1[kCnts/Chnl]	0.139	±0.019	13%
τ_1[ns]	396 000	±33 000	8.1%
I_1[kCnts]	5.34	±0.32	6.0%
	-		
A_2[kCnts/Chnl]	0.619	±0.030	4.7%
τ_2[ns]	64 700	±7 400	11%
I_2[kCnts]	3.91	±0.37	9.5%
	-		
$Bkgr_{Dec}$[kCnts]	0.0044	±0.0002	4.5%
	-		
τ_{AvInt}[ns]	256 000	±12 000	4.3%
	-		

Figure S88. Fitting curve for **Cu1** at room temperature (λ exc = 405 nm) in the solid state.

77 K

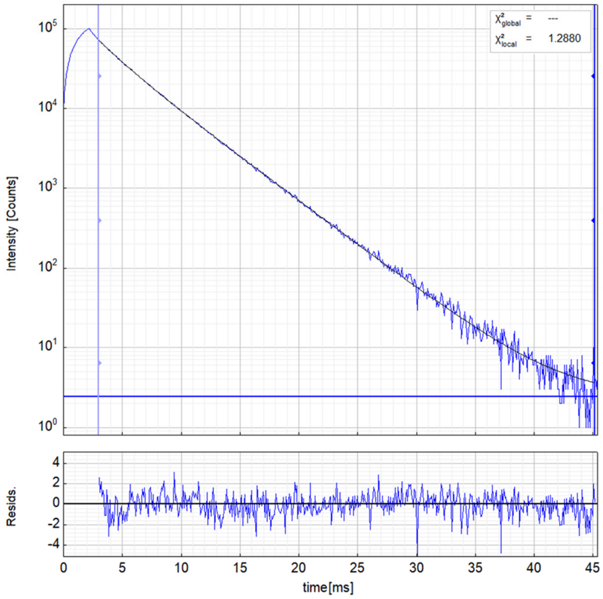

Measurement Context: Decay

Excitation: U pol 405±15nm with PLS-400

Detection: U pol 555±7nm 100000 peak counts

grating 1200/500+

detector UV-red [PMT]

Parameter	Value	Δ	δ
A_1[kCnts/Chnl]	51.48	±0.91	1.8%
τ_1[ns]	3 945 000	±17 000	0.4%
I_1[kCnts]	2 479	±33	1.3%
	-		
A_2[kCnts/Chnl]	19.11	±0.78	4.1%
τ_2[ns]	1 913 000	±66 000	3.4%
I_2[kCnts]	447	±32	7.1%
	-		
$Bkgr_{Dec}$[kCnts]	0.0025	±0.0003	9.4%
	-		
τ_{AvInt}[ns]	3 634 800	±3 200	0.1%
	-		

Figure S89. Fitting curve for **Cu1** at 77 K (λ exc = 405 nm) in the solid state.

Cu2

300 K

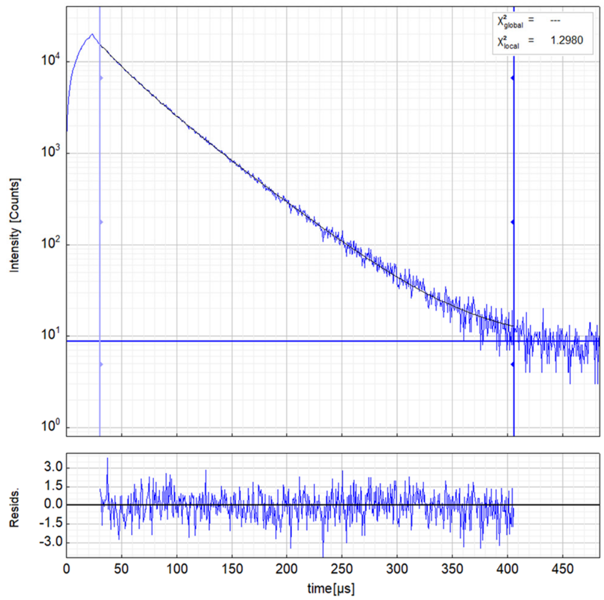

Measurement Context: Decay

Excitation: U pol 405±15nm with PLS-400

Detection: U pol 560±14nm 20000 peak counts

grating 1200/500+

detector UV-red [PMT]

Parameter	Value	Δ	δ
A_1[kCnts/Chnl]	9.09	±0.79	8.6%
τ_1[ns]	48 700	±1 200	2.4%
I_1[kCnts]	691	±42	6.0%
	-		
A_2[kCnts/Chnl]	6.25	±0.72	11%
τ_2[ns]	24 100	±1 800	7.3%
I_2[kCnts]	235	±43	18%
	-		
$Bkgr_{Dec}$[kCnts]	0.0087	±0.0007	7.9%
	-		
τ_{AvInt}[ns]	42 390	±140	0.3%

Figure S90. Fitting curve for **Cu2** at 300 K (λ exc = 405 nm) in the solid state.

80 K

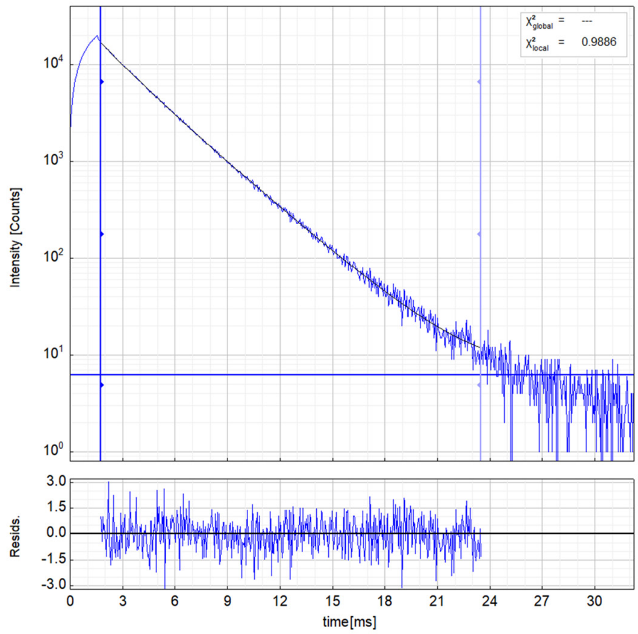

Measurement Context: Decay

Excitation: U pol 405±15nm with PLS-400

Detection: U pol 560±14nm 20000 peak counts

grating 1200/500+

detector UV-red [PMT]

Parameter	Value	Δ	δ
A_1[kCnts/Chnl]	12.38	±0.76	6.1%
τ_1[ns]	2 821 000	±44 000	1.5%
I_1[kCnts]	852	±38	4.4%
	-		
A_2[kCnts/Chnl]	4.53	±0.71	16%
τ_2[ns]	1 530 000	±130 000	8.4%
I_2[kCnts]	169	±39	23%
	-		
$Bkgr_{Dec}$[kCnts]	0.0062	±0.0008	13%
	-		
τ_{AvInt}[ns]	2 606 700	±4 200	0.2%
	-		

Figure S91. Fitting curve for **Cu2** at 80 K (λ exc = 405 nm) in the solid state.

Cu3

300 K

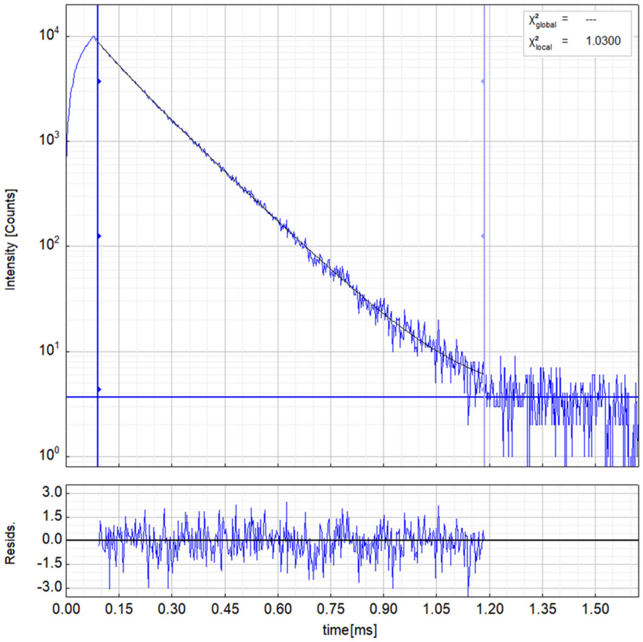

Measurement Context: Decay

Excitation: U pol 405±15nm with PLS-400

Detection: U pol 550±14nm 10000 peak counts

grating 1200/500+

detector UV-red [PMT]

Parameter	Value	Δ	δ
A_1[kCnts/Chnl]	1.94	±0.22	11%
τ_1[ns]	68 500	±3 700	5.3%
I_1[kCnts]	51.9	±8.8	17%
		-	
A_2[kCnts/Chnl]	6.76	±0.22	3.2%
τ_2[ns]	137 800	±1 600	1.1%
I_2[kCnts]	363.6	±8.8	2.4%
		-	
$Bkgr_{Dec}$[kCnts]	0.0037	±0.0005	12%
		-	
τ_{AvInt}[ns]	129 140	±210	0.2%

Figure S92. Fitting curve for **Cu3** at 300 K (λ exc = 405 nm) in the solid state.

80 K

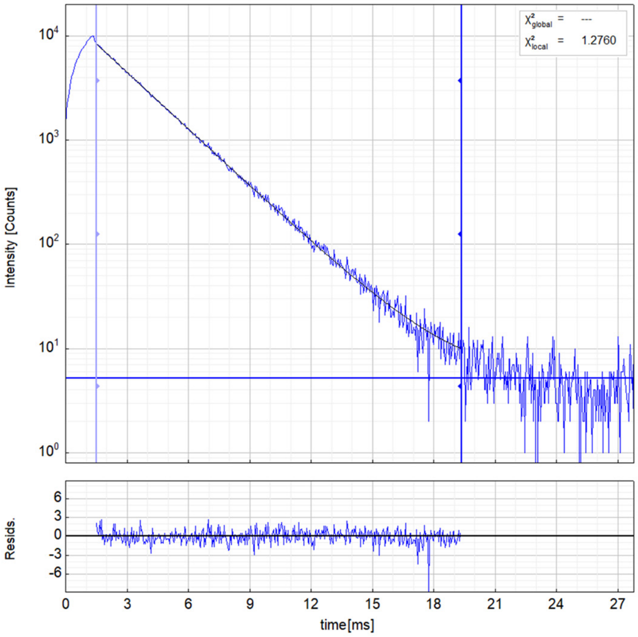

Measurement Context: Decay

Excitation: U pol 405±15nm with PLS-400

Detection: U pol 550±14nm 10000 peak counts

grating 1200/500+

detector UV-red [PMT]

Parameter	Value	Δ	δ
A_1[kCnts/Chnl]	8.316	±0.025	0.3%
τ_1[ns]	2 387 600	±3 700	0.2%
I_1[kCnts]	484.70	±0.45	0.1%
		-	
$Bkgr_{Dec}$[kCnts]	0.0052	±0.0004	7.7%
		-	
τ_{AvInt}[ns]	2 387 600	±3 700	0.2%

Figure S93. Fitting curve for **Cu3** at 80 K (λ exc = 405 nm) in the solid state.

Cu4

Rt

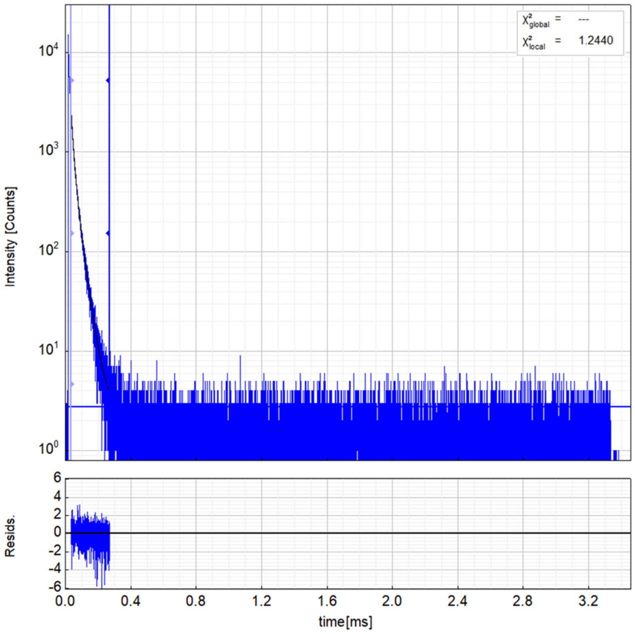

Measurement Context: Decay

Excitation: U pol 370±5nm with Xe-Lamp

Detection: U pol 540±5nm 15000 peak counts

grating 1200/500+

detector UV-red [PMT]

Parameter	Value	Δ	δ
A_1[kCnts/Chnl]	0.853	±0.028	3.2%
τ_1[ns]	35 970	±360	1.0%
I_1[kCnts]	191.8	±4.0	2.1%
		-	
A_2[kCnts/Chnl]	1.939	±0.022	1.1%
τ_2[ns]	12 700	±190	1.5%
I_2[kCnts]	153.9	±3.6	2.3%
		-	
$Bkgr_{Dec}$[kCnts]	0.0028	±0.0002	4.3%
		-	
τ_{AvInt}[ns]	25 610	±120	0.5%
		-	

Figure S94. Fitting curve for **Cu4** at room temperature (λ exc = 370 nm) in the solid state.

77 K

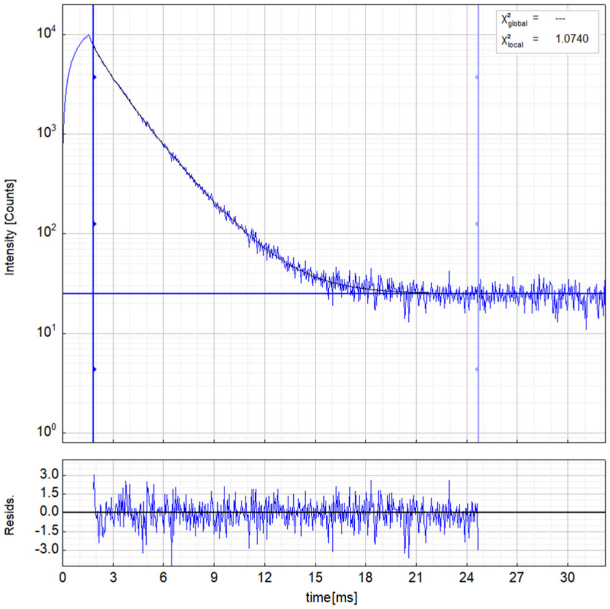

Measurement Context: Decay

Excitation: U pol 378±10nm with PLS-370

Detection: U pol 548±14nm 10000 peak counts

grating 1200/500+

detector UV-red [PMT]

Parameter	Value	Δ	δ
A_1[kCnts/Chnl]	4.82	±0.21	4.2%
τ_1[ns]	2 193 000	±33 000	1.5%
I_1[kCnts]	257.9	±6.9	2.7%
		-	
A_2[kCnts/Chnl]	2.82	±0.26	9.0%
τ_2[ns]	927 000	±49 000	5.2%
I_2[kCnts]	63.7	±7.0	11%
		-	
$Bkgr_{Dec}$[kCnts]	0.0250	±0.0006	2.1%
		-	
τ_{AvInt}[ns]	1 941 800	±8 500	0.4%

Figure S95. Fitting curve for **Cu4** at 77 K (λ exc = 370 nm) in the solid state.

Cu5

Rt

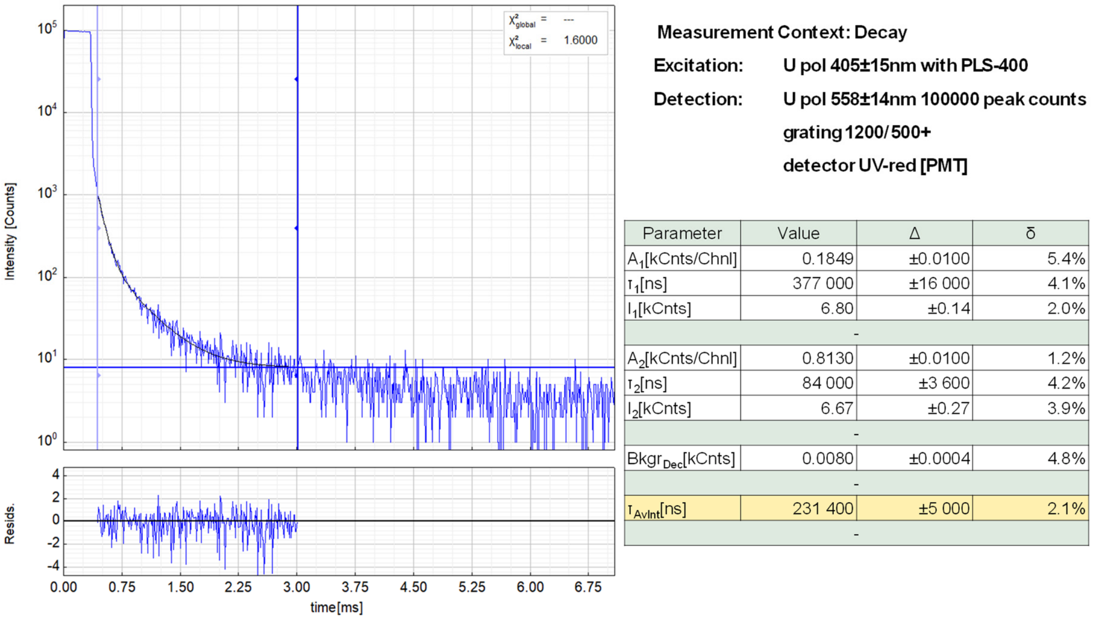

Measurement Context: Decay

Excitation: U pol 405±15nm with PLS-400

Detection: U pol 558±14nm 100000 peak counts

grating 1200/500+

detector UV-red [PMT]

Parameter	Value	Δ	δ
A_1[kCnts/Chnl]	0.1849	±0.0100	5.4%
τ_1[ns]	377 000	±16 000	4.1%
I_1[kCnts]	6.80	±0.14	2.0%
		-	
A_2[kCnts/Chnl]	0.8130	±0.0100	1.2%
τ_2[ns]	84 000	±3 600	4.2%
I_2[kCnts]	6.67	±0.27	3.9%
		-	
$Bkgr_{Dec}$[kCnts]	0.0080	±0.0004	4.8%
		-	
τ_{AvInt}[ns]	231 400	±5 000	2.1%
		-	

Figure S96. Fitting curve for **Cu5** at room temperature (λ exc = 405 nm) in the solid state.

77 K

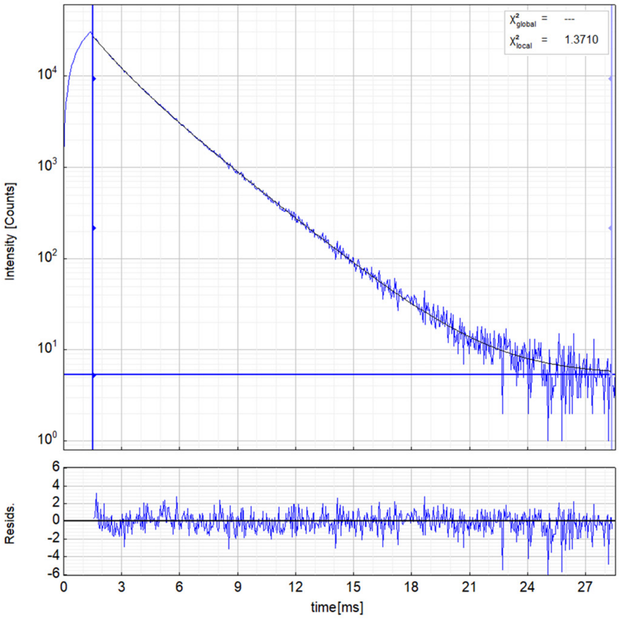

Measurement Context: Decay

Excitation: U pol 378±10nm with PLS-370

Detection: U pol 579±14nm 30000 peak counts

grating 1200/500+

detector UV-red [PMT]

Parameter	Value	Δ	δ
A_1[kCnts/Chnl]	15.11	±0.47	3.1%
τ_1[ns]	2 599 000	±20 000	0.8%
I_1[kCnts]	959	±21	2.2%
		-	
A_2[kCnts/Chnl]	11.33	±0.43	3.7%
τ_2[ns]	1 243 000	±30 000	2.3%
I_2[kCnts]	344	±20	5.6%
		-	
$Bkgr_{Dec}$[kCnts]	0.0054	±0.0003	4.6%
		-	
τ_{AvInt}[ns]	2 240 200	±2 600	0.1%
		-	

Figure S97. Fitting curve for **Cu5** at 77 K (λ exc = 370 nm) in the solid state.

Cu6

Rt

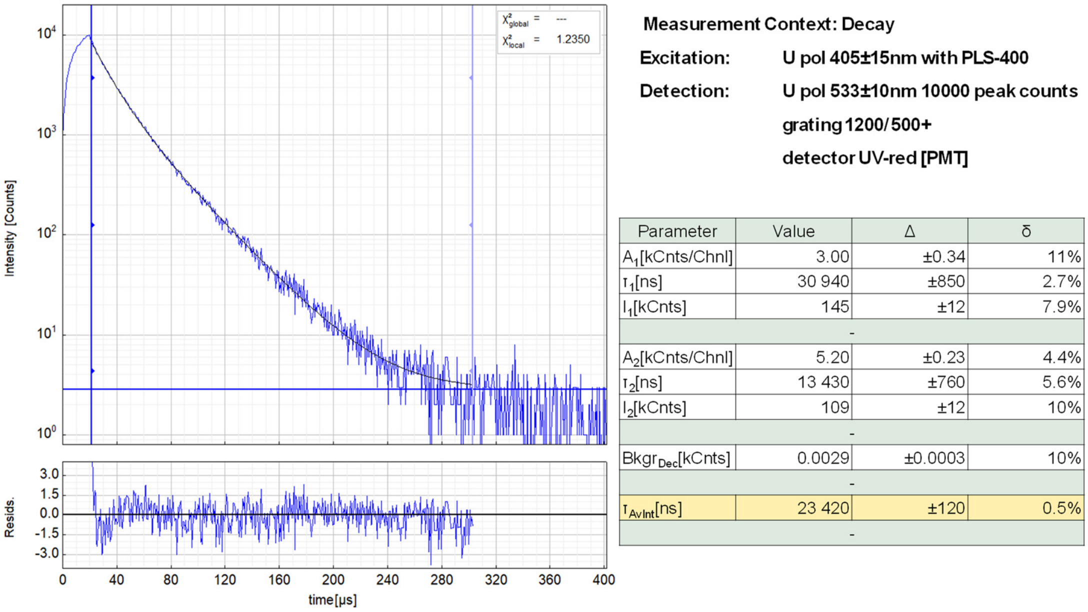

Measurement Context: Decay

Excitation: U pol 405±15nm with PLS-400

Detection: U pol 533±10nm 10000 peak counts
grating 1200/500+
detector UV-red [PMT]

Parameter	Value	Δ	δ
A_1[kCnts/Chnl]	3.00	±0.34	11%
τ_1[ns]	30 940	±850	2.7%
I_1[kCnts]	145	±12	7.9%
	-		
A_2[kCnts/Chnl]	5.20	±0.23	4.4%
τ_2[ns]	13 430	±760	5.6%
I_2[kCnts]	109	±12	10%
	-		
$Bkgr_{Dec}$[kCnts]	0.0029	±0.0003	10%
	-		
τ_{AvInt}[ns]	23 420	±120	0.5%
	-		

Figure S98. Fitting curve for **Cu6** at room temperature (λ exc = 405 nm) in the solid state.

77 K

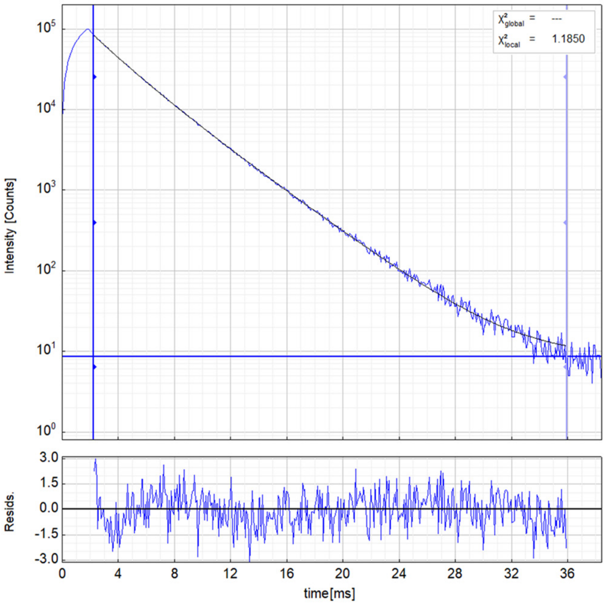

Measurement Context: Decay

Excitation: U pol 405±15nm with PLS-400

Detection: U pol 550±7nm 100000 peak counts
grating 1200/500+
detector UV-red [PMT]

Parameter	Value	Δ	δ
A_1[kCnts/Chnl]	48.0	±1.9	3.8%
τ_1[ns]	3 497 000	±30 000	0.8%
I_1[kCnts]	2 046	±59	2.9%
	-		
A_2[kCnts/Chnl]	36.7	±1.8	4.8%
τ_2[ns]	2 039 000	±48 000	2.3%
I_2[kCnts]	913	±60	6.5%
	-		
$Bkgr_{Dec}$[kCnts]	0.0086	±0.0003	2.7%
	-		
τ_{AvInt}[ns]	3 046 900	±3 000	0.1%
	-		

Figure S99. Fitting curve for **Cu6** at 77 K (λ exc = 405 nm) in the solid state.

Cu8

300 K

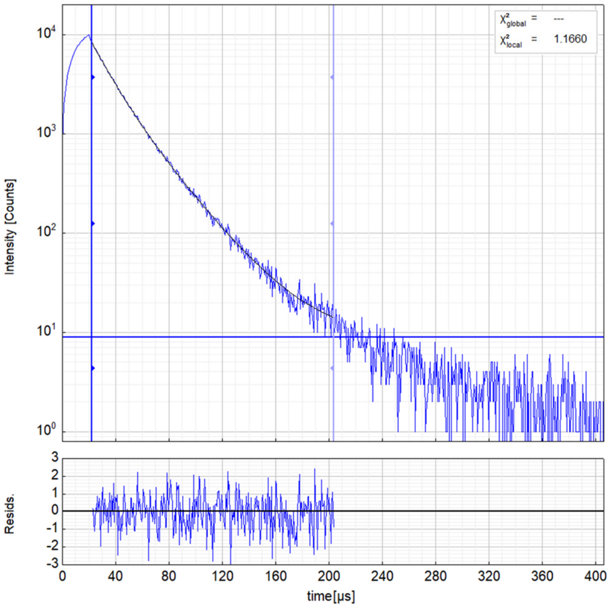

Measurement Context: Decay

Excitation: U pol 405±15nm with PLS-400

Detection: U pol 540±14nm 10000 peak counts

grating 1200/ 500+

detector UV-red [PMT]

Parameter	Value	Δ	δ
A_1[kCnts/Chnl]	3.23	±0.62	19%
τ_1[ns]	28 100	±1 800	6.3%
I_1[kCnts]	142	±20	14%
		-	
A_2[kCnts/Chnl]	4.94	±0.57	12%
τ_2[ns]	14 180	±870	6.1%
I_2[kCnts]	110	±21	18%
		-	
$Bkgr_{Dec}$[kCnts]	0.0090	±0.0015	16%
		-	
τ_{AvInt}[ns]	22 030	±180	0.8%
		-	

Figure S100. Fitting curve for **Cu8** at 300 K (λ exc = 405 nm) in the solid state.

80 K

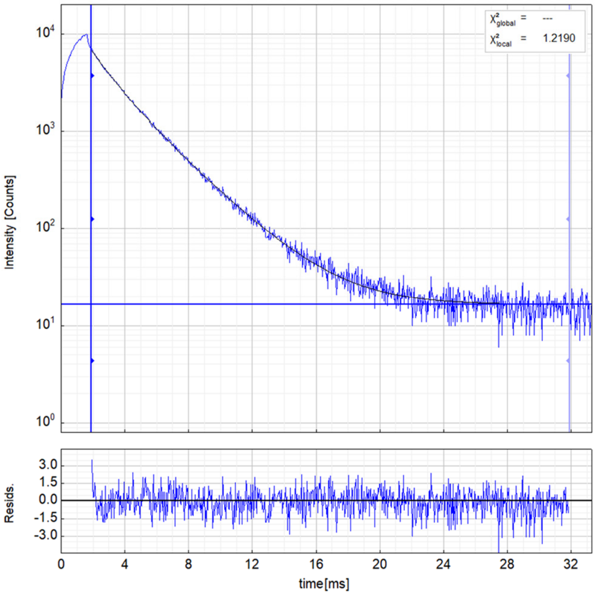

Measurement Context: Decay

Excitation: U pol 405±15nm with PLS-400

Detection: U pol 540±14nm 10000 peak counts

grating 1200/ 500+

detector UV-red [PMT]

Parameter	Value	Δ	δ
A_1[kCnts/Chnl]	3.91	±0.40	10%
τ_1[ns]	2 815 000	±80 000	2.8%
I_1[kCnts]	269	±20	7.3%
		-	
A_2[kCnts/Chnl]	2.80	±0.34	12%
τ_2[ns]	1 310 000	±120 000	8.8%
I_2[kCnts]	90	±20	22%
		-	
$Bkgr_{Dec}$[kCnts]	0.0165	±0.0004	2.0%
		-	
τ_{AvInt}[ns]	2 439 000	±13 000	0.5%
		-	

Figure S101. Fitting curve for **Cu8** at 80 K (λ exc = 405 nm) in the solid state.

Cu9

Rt

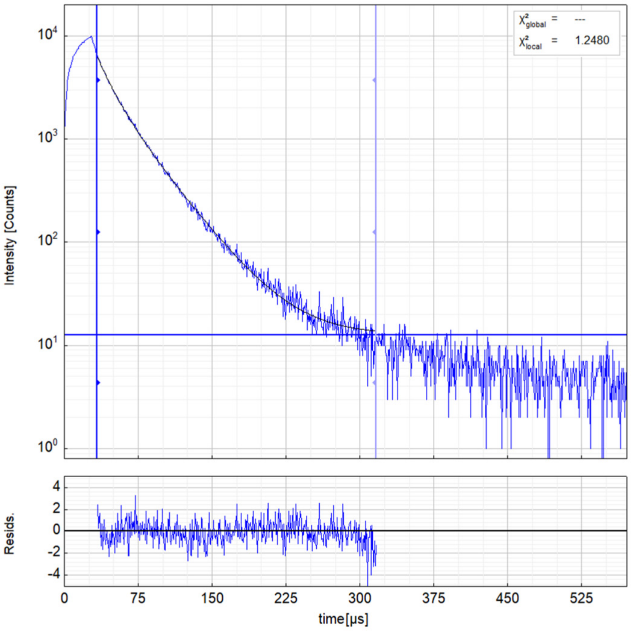

Measurement Context: Decay

Excitation: U pol 405±15nm with PLS-400

Detection: U pol 538±14nm 10000 peak counts

grating 1200/500+

detector UV-red [PMT]

Parameter	Value	Δ	δ
A_1[kCnts/Chnl]	3.22	±0.25	7.6%
τ_1[ns]	35 320	±950	2.7%
I_1[kCnts]	177.2	±8.7	4.9%
		-	
A_2[kCnts/Chnl]	2.98	±0.24	7.8%
τ_2[ns]	13 600	±1 100	7.8%
I_2[kCnts]	62.8	±9.0	14%
		-	
$Bkgr_{Dec}$[kCnts]	0.0127	±0.0008	5.9%
		-	
τ_{AvInt}[ns]	29 610	±160	0.5%

Figure S102. Fitting curve for **Cu9** at room temperature (λ exc = 405 nm) in the solid state.

77 K

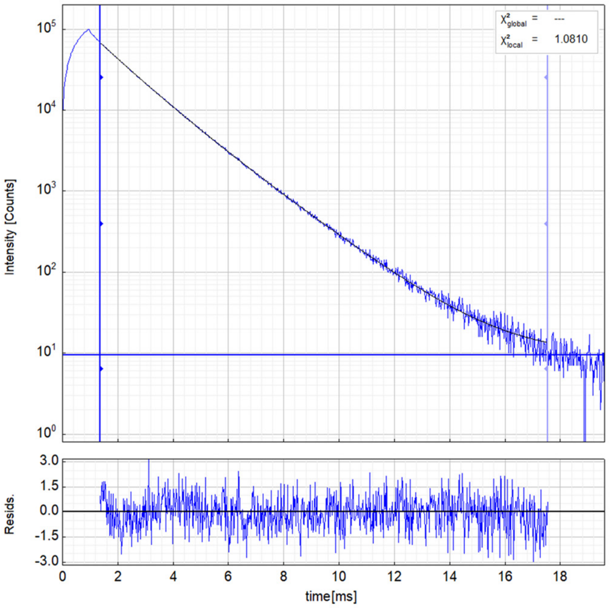

Measurement Context: Decay

Excitation: U pol 405±15nm with PLS-400

Detection: U pol 555±8nm 100000 peak counts

grating 1200/500+

detector UV-red [PMT]

Parameter	Value	Δ	δ
A_1[kCnts/Chnl]	36.7	±2.8	7.4%
τ_1[ns]	1 154 000	±29 000	2.5%
I_1[kCnts]	2 070	±210	10%
		-	
A_2[kCnts/Chnl]	30.9	±2.9	9.3%
τ_2[ns]	1 806 000	±32 000	1.7%
I_2[kCnts]	2 730	±210	7.7%
		-	
$Bkgr_{Dec}$[kCnts]	0.0096	±0.0005	5.0%
		-	
τ_{AvInt}[ns]	1 524 400	±1 500	0.1%
		-	

Figure S103. Fitting curve for **Cu9** at 77 K (λ exc = 405 nm) in the solid state.

Cu10

Rt

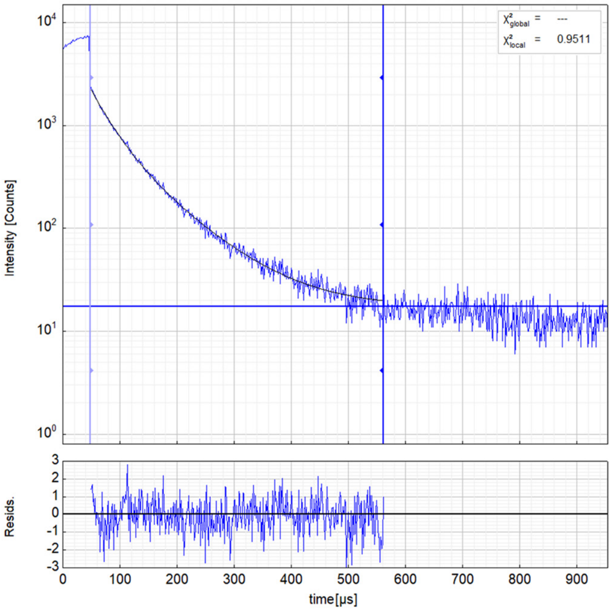

Measurement Context: Decay

Excitation: U pol 405±15nm with PLS-400

Detection: U pol 700±14nm 20000 peak counts

grating 1200/500+

detector UV-red [PMT]

Parameter	Value	Δ	δ
A_1[kCnts/Chnl]	1.39	±0.11	7.7%
τ_1[ns]	33 000	±2 600	7.8%
I_1[kCnts]	35.8	±5.8	16%
		-	
A_2[kCnts/Chnl]	0.82	±0.13	15%
τ_2[ns]	88 100	±6 000	6.7%
I_2[kCnts]	55.8	±5.3	9.3%
		-	
$Bkgr_{Dec}$[kCnts]	0.0174	±0.0010	5.2%
		-	
τ_{AvInt}[ns]	66 600	±1 100	1.6%

Figure S104. Fitting curve for **Cu10** at room temperature (λ exc = 405 nm) in the solid state.

77 K

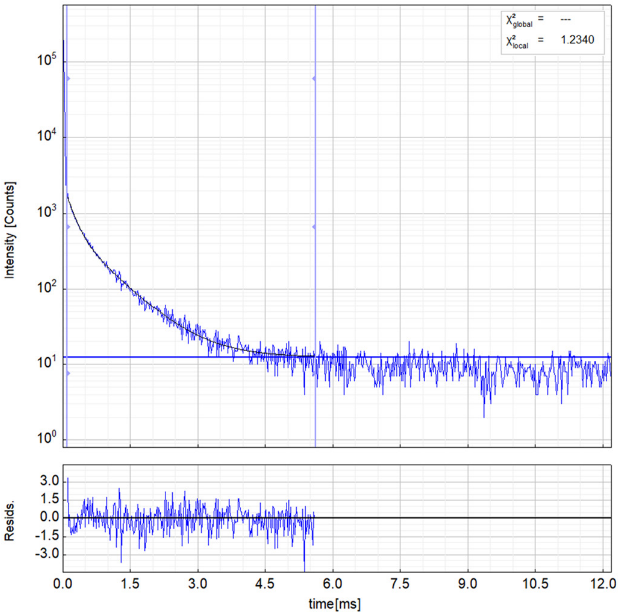

Measurement Context: Decay

Excitation: U pol 370±14nm with Xe-Lamp

Detection: U pol 700±14nm 500000 peak counts

grating 1200/500+

detector UV-red [PMT]

Parameter	Value	Δ	δ
A_1[kCnts/Chnl]	0.593	±0.027	4.4%
τ_1[ns]	738 000	±20 000	2.6%
I_1[kCnts]	21.34	±0.67	3.1%
		-	
A_2[kCnts/Chnl]	1.043	±0.038	3.6%
τ_2[ns]	174 000	±12 000	6.5%
I_2[kCnts]	8.82	±0.52	5.8%
		-	
$Bkgr_{Dec}$[kCnts]	0.0125	±0.0005	3.6%
		-	
τ_{AvInt}[ns]	572 300	±8 200	1.4%
		-	

Figure S105. Fitting curve for **Cu10** at 77 K (λ exc = 370 nm) in the solid state.

Cu11

Rt

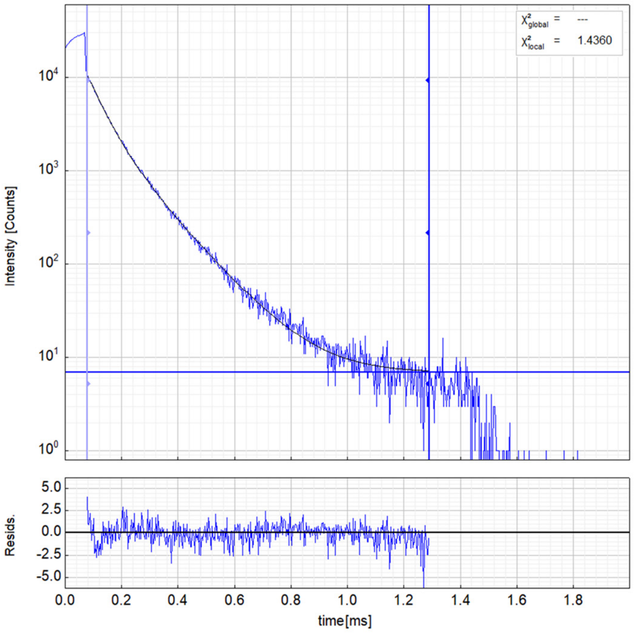

Measurement Context: Decay

Excitation: U pol 405±15nm with PLS-400

Detection: U pol 698±14nm 30000 peak counts

grating 1200/500+

detector UV-red [PMT]

Parameter	Value	Δ	δ
A_1[kCnts/Chnl]	3.24	±0.17	5.1%
τ_1[ns]	130 400	±2 100	1.6%
I_1[kCnts]	164.9	±6.1	3.6%
	-		
A_2[kCnts/Chnl]	7.24	±0.17	2.3%
τ_2[ns]	53 400	±1 100	2.0%
I_2[kCnts]	151.0	±6.2	4.1%
	-		
$Bkgr_{Dec}$[kCnts]	0.0069	±0.0004	4.8%
	-		
τ_{AvInt}[ns]	93 560	±200	0.2%
	-		

Figure S106. Fitting curve for **Cu11** at room temperature (λ exc = 405 nm) in the solid state.

77 K

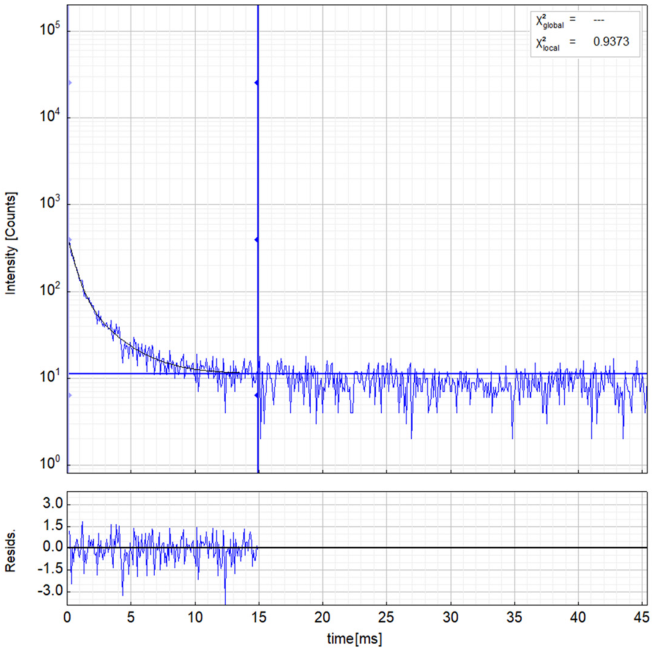

Measurement Context: Decay

Excitation: U pol 380±14nm with Xe-Lamp

Detection: U pol 680±14nm 100000 peak counts

grating 1200/500+

detector UV-red [PMT]

Parameter	Value	Δ	δ
A_1[kCnts/Chnl]	0.263	±0.015	5.5%
τ_1[ns]	589 000	±44 000	7.4%
I_1[kCnts]	1.89	±0.23	12%
	-		
A_2[kCnts/Chnl]	0.092	±0.013	13%
τ_2[ns]	2 410 000	±280 000	11%
I_2[kCnts]	2.69	±0.18	6.6%
	-		
$Bkgr_{Dec}$[kCnts]	0.0113	±0.0005	4.3%
	-		
τ_{AvInt}[ns]	1 660 000	±130 000	7.3%
	-		

Figure S107. Fitting curve for **Cu11** at 77 K (λ exc = 380 nm) in the solid state.

S6.- TADF fitting

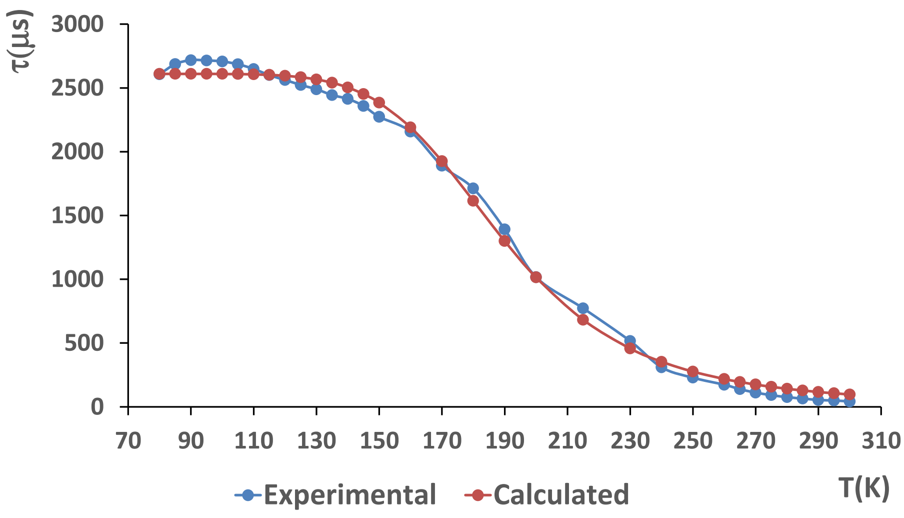

Figure S108. Fitting curve for **Cu2** (Chi Square: 0.996) in the solid state.

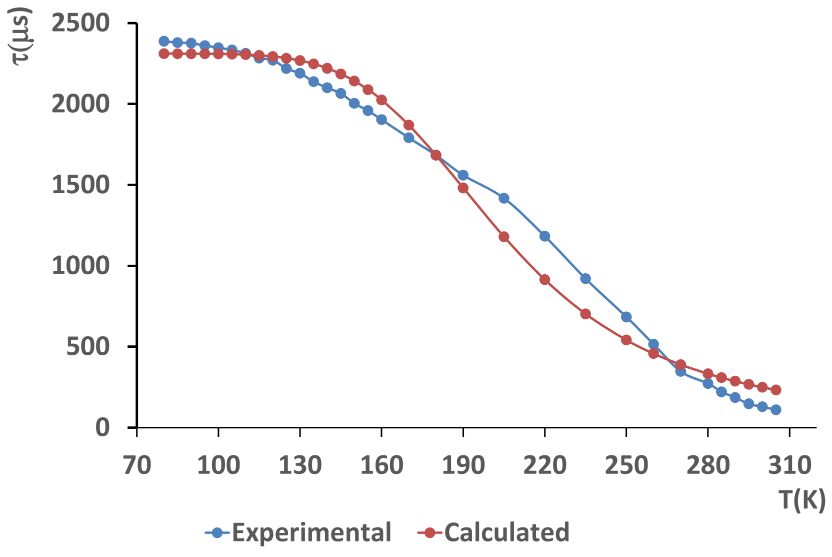

Figure S109. Fitting curve for **Cu3** (Chi Square: 0.982) in the solid state.

S7.- CIE 1931 x, y coordinates

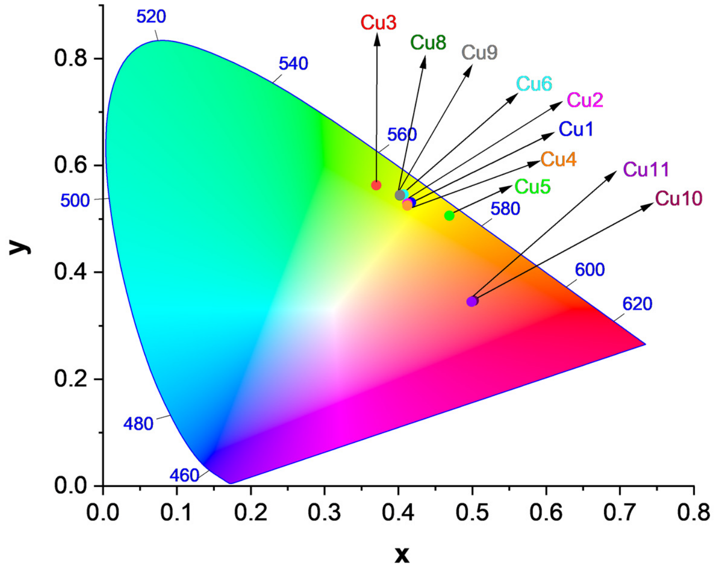

Figure S110. CIE 1931 xy coordinates for copper complexes in the solid state.

S8.- Thermal gravimetric analysis (TGA) curves

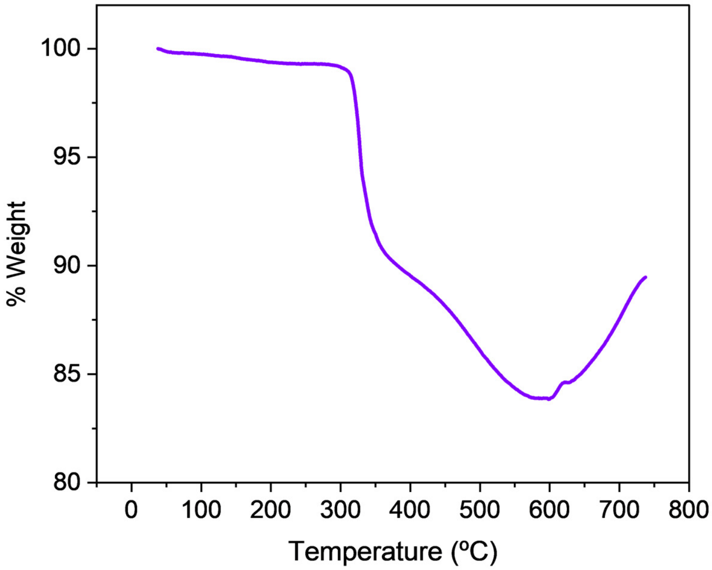

Figure S111. Thermal gravimetric analysis (TGA) curve of **Cu2**.

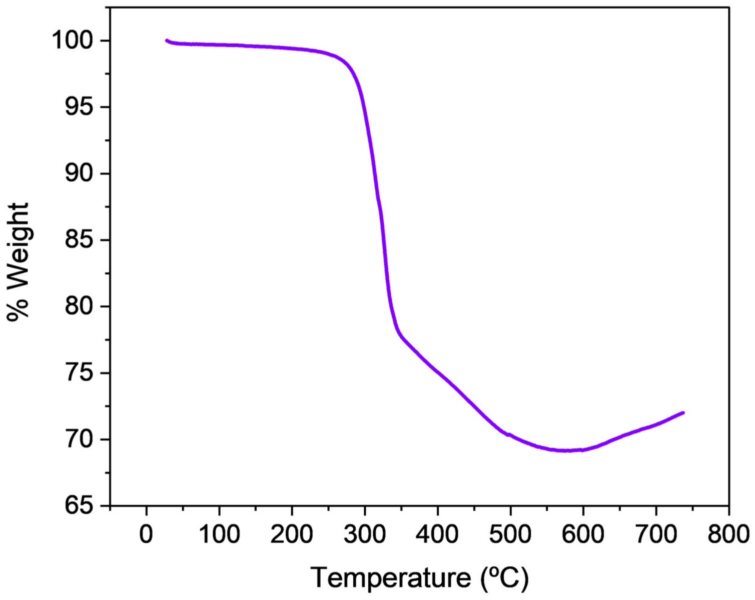

Figure S112. Thermal gravimetric analysis (TGA) curve of **Cu3**.

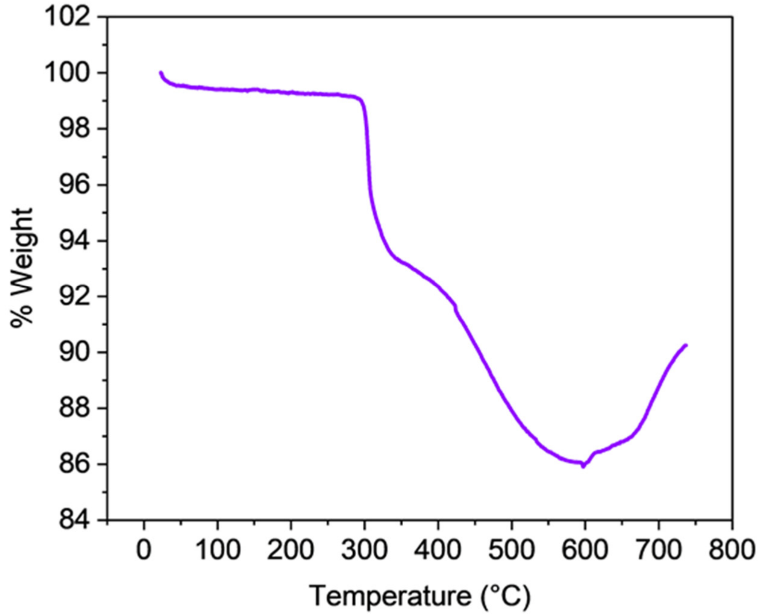

Figure S113. Thermal gravimetric analysis (TGA) curve of **Cu8**.

S9.- % Buried volumes

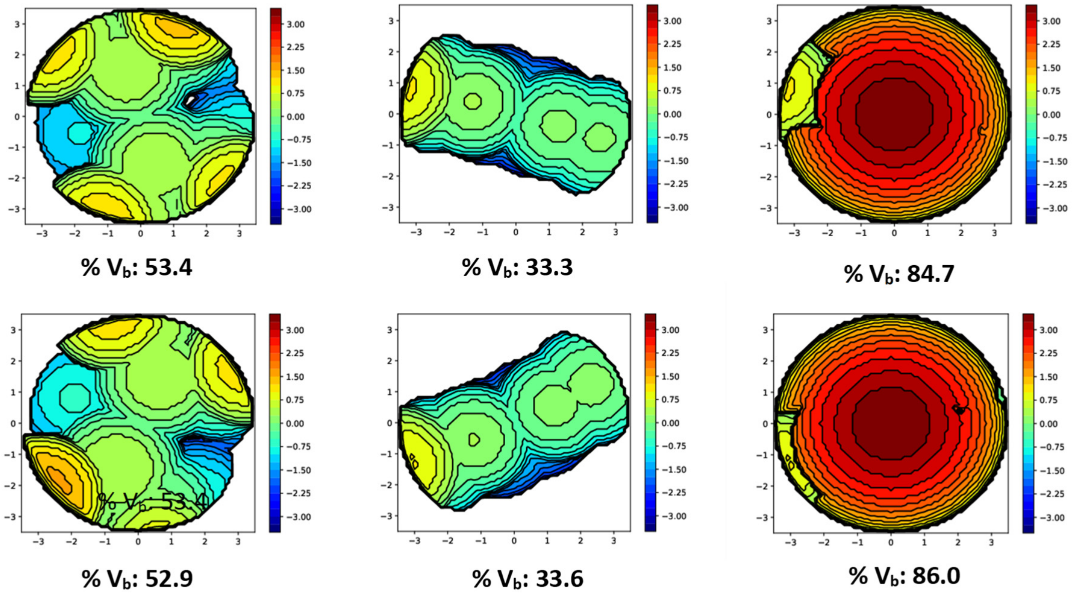

Figura S114. % Buried volume for **Cu2. Right:** diphosphane. **Centre:** diimine. **Right:** total.

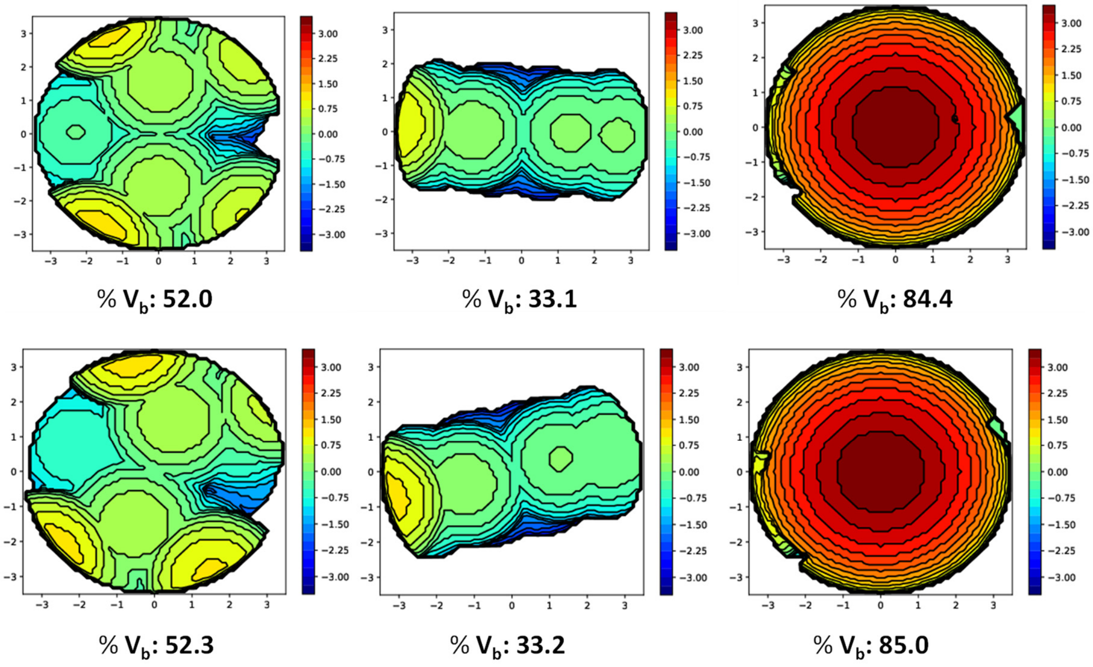

Figura S115. % Buried volume for **Cu3. Right:** diphosphane. **Centre:** diimine. **Right:** total.

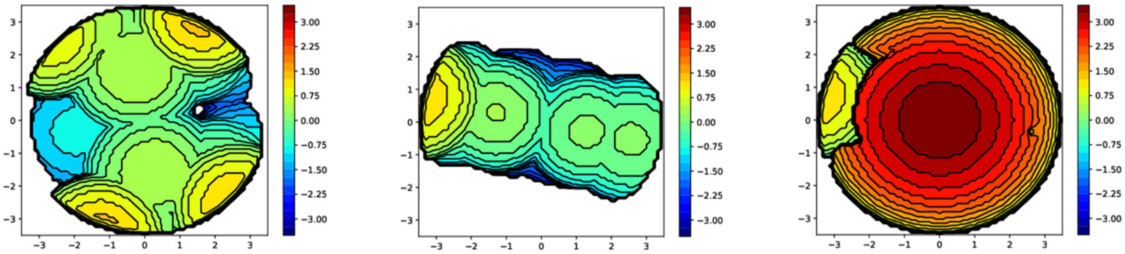

% V_b: 52.7 | % V_b: 33.8 | % V_b: 84.1

Figura S116. % Buried volume for **Cu4. Right:** diphosphane. **Centre:** diimine. **Right:** total

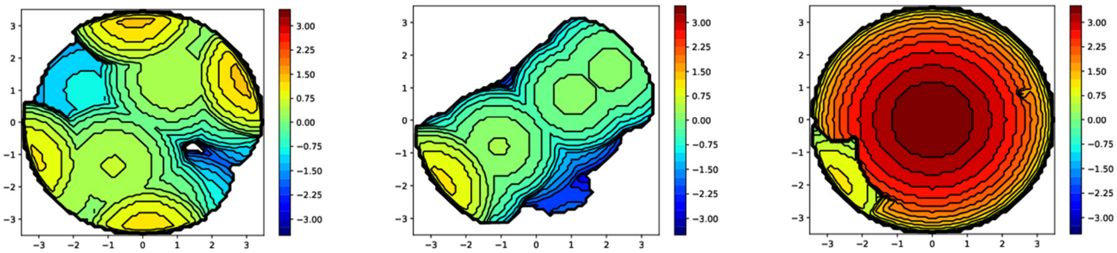

% V_b: 53.4 | % V_b: 34.0 | % V_b: 85.1

Figura S117. % Buried volume for **Cu5. Right:** diphosphane. **Centre:** diimine. **Right:** total.

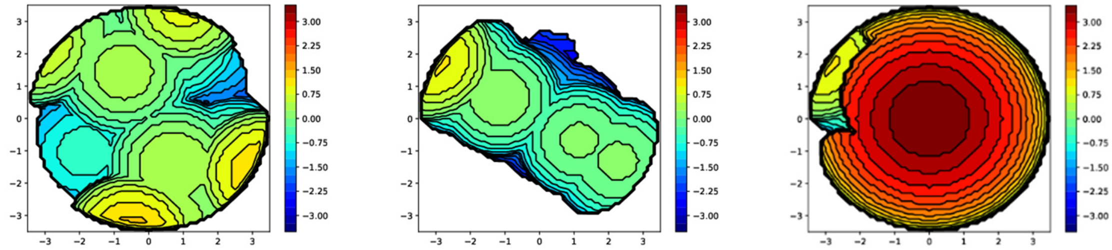

% V_b: 51.3 | % V_b: 33.4 | % V_b: 82.9

Figura S118. % Buried volume for **Cu6. Right:** diphosphane. **Centre:** diimine. **Right:** total.

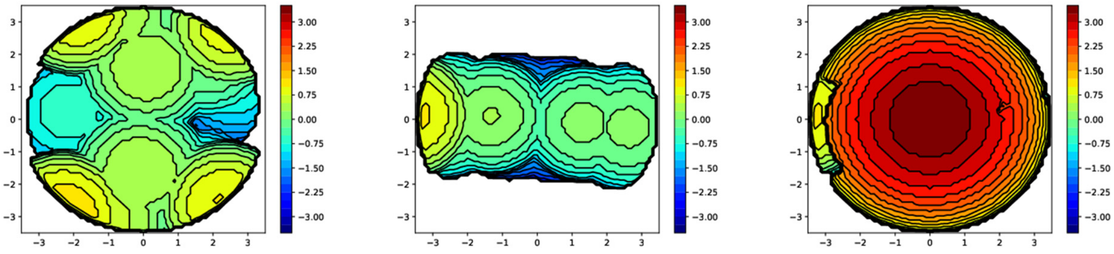

% V_b: 52.8 | % V_b: 33.5 | % V_b: 85.0

Figura S119. % Buried volume for **Cu8. Right:** diphosphane. **Centre:** diimine. **Right:** total.

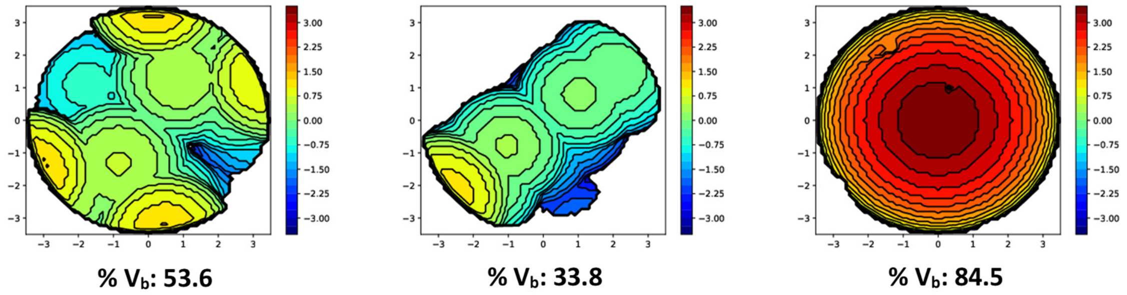

Figura S120. % Buried volume for **Cu9. Right:** diphosphane. **Centre:** diimine. **Right:** total.

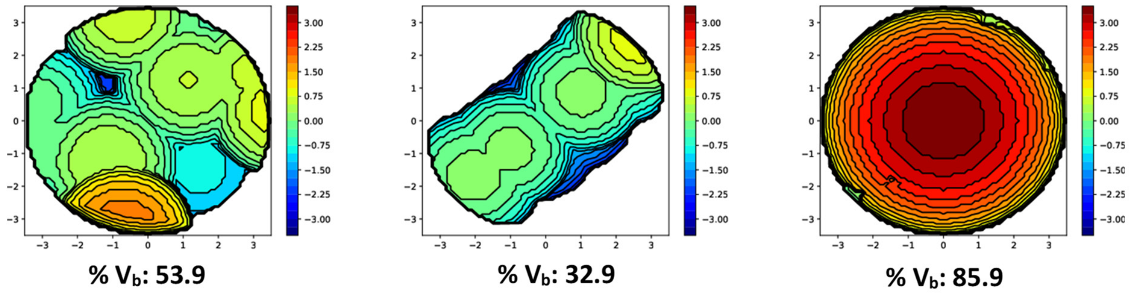

Figura S121. % Buried volume for **Cu11. Right:** diphosphane. **Centre:** diimine. **Right:** total.

S10.- Crystal X-ray data: Diagrams, bond distances (Å) and angles (°)

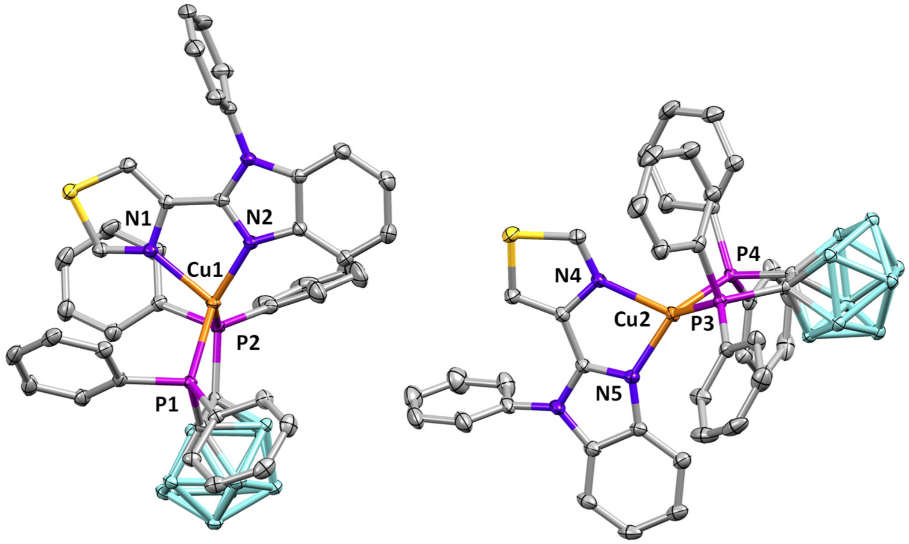

Figure S122. Diagrams of **Cu2**. Hydrogen atoms have been omitted for clarity. Ortep diagram showing the labeling scheme. Ellipsoids represent 50 % probability level. **Left**: molecule 1. Bond distances (Å) and lengths (°) Cu1-P1 2.2191(5), Cu1-P2 2.2301(6), N1-Cu1 2.1188(15), N2-Cu1 2.0031(14), N1-Cu1-N2 80.06(12), P2-Cu1-P1 92.325(18). α = 89.54. **Right**: molecule 2. Bond distances (Å) and lengths (°) Cu2-P3 2.2033(6), Cu2-P4 2.2283(5), N4-Cu2 2.0874(15), N5-Cu2 2.0076(14), N5-Cu2-N4 81.34(6), P3-Cu2-P4 92.738(17). α = 89.96°.

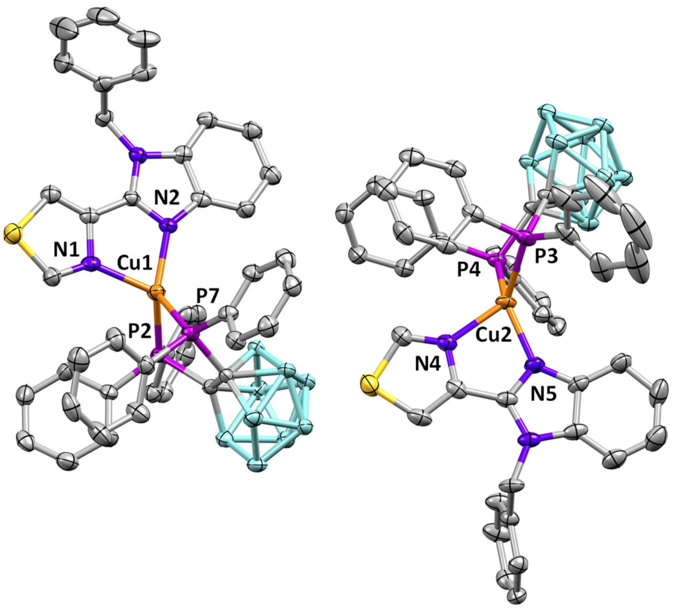

Figure S123. Diagrams of **Cu3**. Hydrogen atoms have been omitted for clarity. Ortep diagram showing the labeling scheme. Ellipsoids represent 50 % probability level. **Left**: molecule 1. Bond distances (Å) and lengths (°): Cu1-N1 2.115(6), Cu1-N2 2.029(5), Cu1-P2 2.2562(18), Cu1-P7 2.2446(18), P7-Cu1-P2 89.08(6), N2-Cu1-N1 80.7(2), α = 87.90°. **Right**: molecule 2. Bond distances (Å) and lengths (°): Cu2-N4 2.144(6), Cu2-N5 2.014(6), Cu2-P3 2.2498(19), Cu2-P4 2.2388(17), P4-Cu2-P3 90.09(6), N5-Cu2-N4 79.0(2), α = 85.53°.

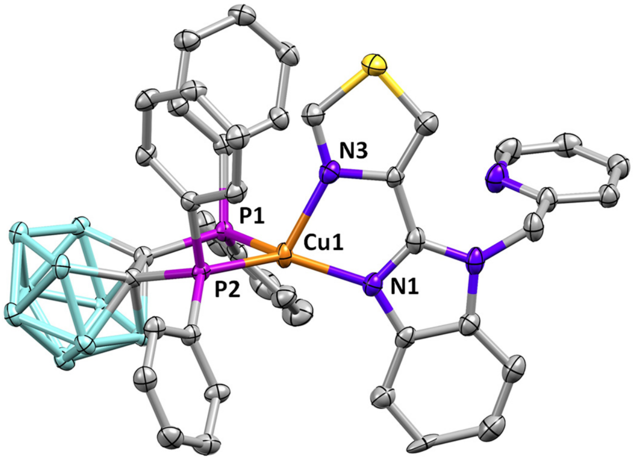

Figure S124. Diagram of **Cu4**. Hydrogen atoms have been omitted for clarity. Ortep diagram showing the labeling scheme. Ellipsoids represent 50 % probability level. Bond distances (Å) and lengths (°) Cu1-P1 2.2373(9), Cu1-P2 2.2093(10), N1-Cu1 1.989(3), N3-Cu1 2.111(3), N1-Cu1-N3 80.06(12), P2-Cu1-P1 91.76(3). α = 89.41°.

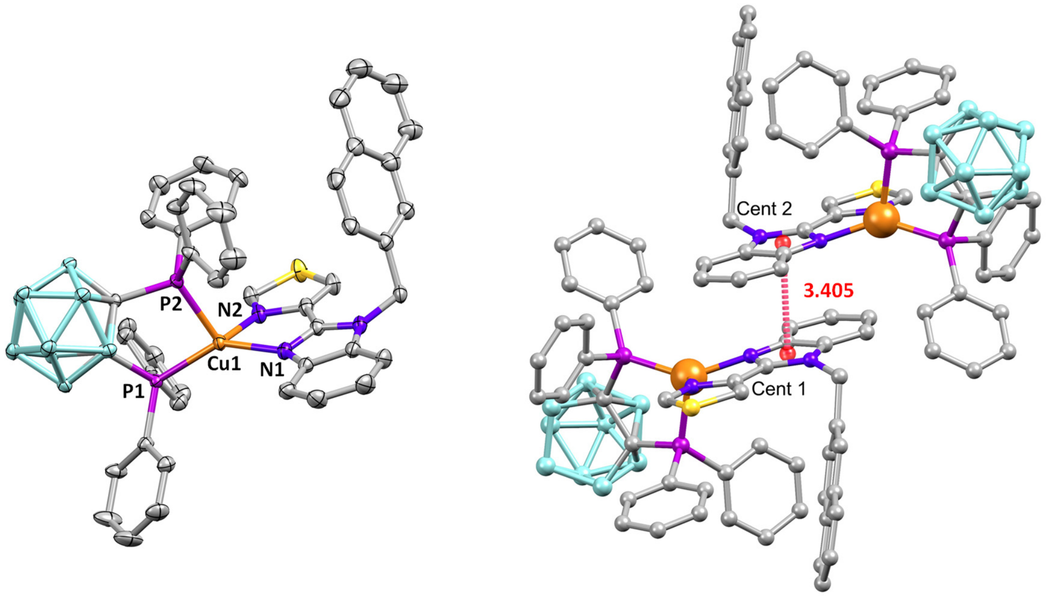

Figure S125. Diagrams of **Cu5.** Hydrogen atoms have been omitted for clarity. **Left**: Ortep diagram showing the labeling scheme. Ellipsoids represent 50 % probability level. Bond distances (Å) and lengths (°) Cu1-P1 2.1920(6), Cu1 P2 2.2474(6), N1-Cu1 1.996(2), N2-Cu1 2.094(2), N2-Cu1-N1 81.22(8), P2-Cu1-P1 91.66(2). α = 88.58°. **Right**: Molecules associate in dimers through π-π interactions involving pyrazol fragments of R-tbz fragments of different molecules (distance of 3.405 Å between centroids).

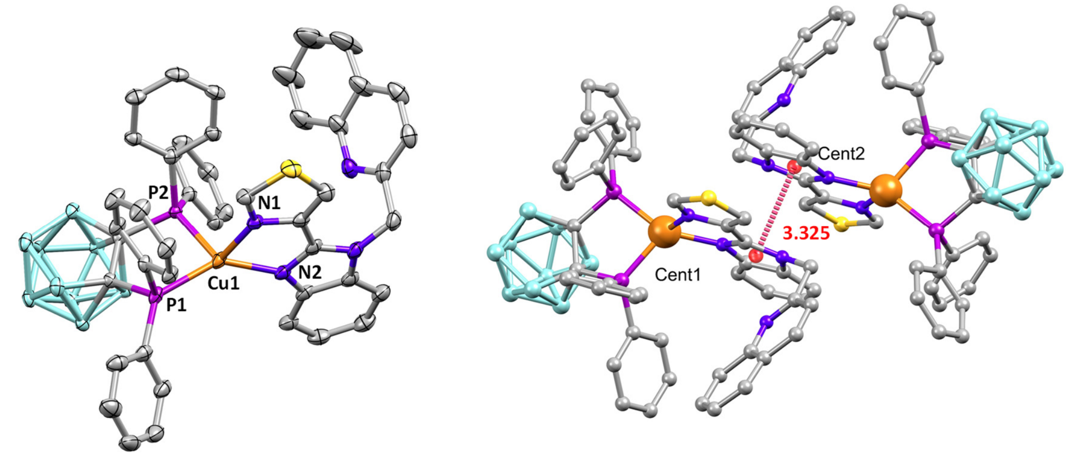

Figure S126. Diagrams of **Cu6.** Hydrogen atoms have been omitted for clarity. **Left**: Ortep diagram showing the labeling scheme. Ellipsoids represent 50 % probability level. Bond distances (Å) and lengths (°) Cu1-P1 2.2982(10), Cu1 -P2 2.2432(8), N1-Cu1 2.116(3), N2-Cu1 2.016(3), N2-Cu1-N1 80.35(10), P2-Cu1-P1 90.70(3). α = 88.83°. **Right**: Molecules associate in dimers through π-π interactions involving pyrazol fragments of R-tbz fragments of different molecules (distance of 3.325 Å between centroids).

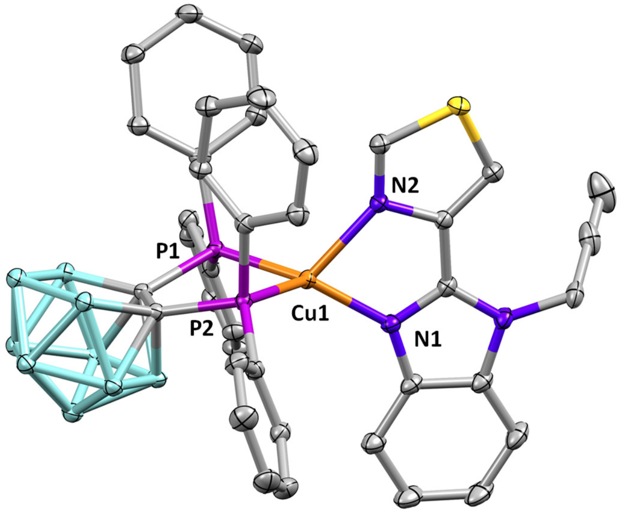

Figure S127. Diagram of **Cu8**. Hydrogen atoms have been omitted for clarity. Ortep diagram showing the labeling scheme. Ellipsoids represent 50 % probability level. Bond distances (Å) and lengths (°) Cu1-P1 2.2186(4), Cu1-P2 2.2297(4), N1-Cu1 2.0037(13), N2-Cu1 2.0998(14), N2-Cu1-N1 81.26(5), P2-Cu1-P1 91.551(16). α = 82.38°.

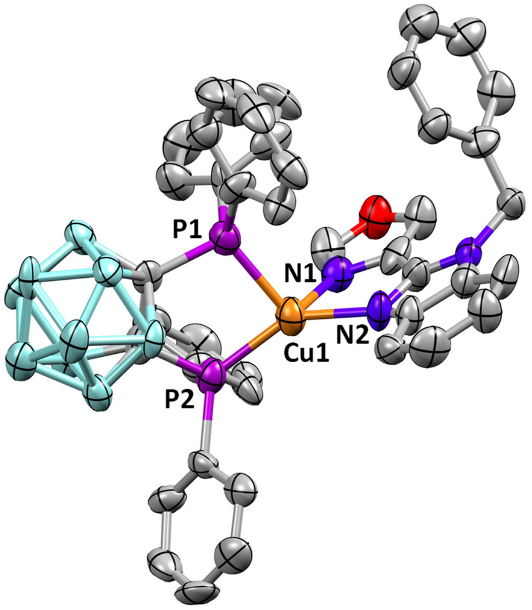

Figure S128. Diagram of **Cu9.** The data were not deposited as explained in the text. Space group P-1. Cell: a = 12.01(4), b=12.84(3) c=14.93(4); α=90.23(14) β=97.47(4) γ=97.14(5). Hydrogen atoms have been omitted for clarity. Ortep diagram showing the labeling scheme. Ellipsoids represent 50 % probability level. Bond distances (Å) and lengths (°) Cu1-P1 2.231(6), Cu1-P2 2.164(7), N1-Cu1 2.109(14), N2-Cu1 1.974(14), N1-Cu1-N2 79.7(6), P1-Cu1-P2 91.4(3). α = 89.74°.

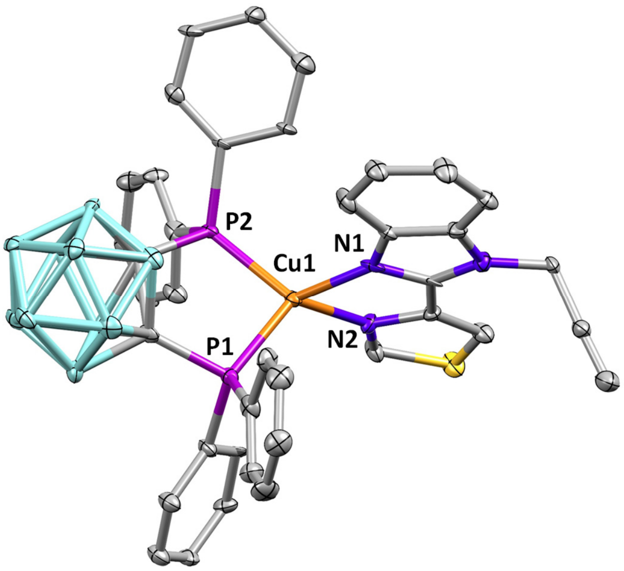

Figure S129. Diagram of the cation **Cu11**. Hydrogen atoms have been omitted for clarity. Ortep diagram showing the labeling scheme. Ellipsoids represent 50 % probability level. Bond distances (Å) and lengths (°) Cu1-P1 2.268(2), Cu1-P2 2.273(2), N1-Cu1 2.072(7), N2-Cu1 2.085(6), N1-Cu1-N2 80.2(2), P1-Cu1-P2 96.83(7). α = 87.29°.

Table S1. Resume of bond distances (Å) and angles (°) for complexes **Cu2-Cu6**, **Cu8, Cu9** and **Cu11**.

**Compound**	**Cu-P**	**Cu-N**	**P-Cu-P**	**N-Cu-N**	α^a
**Cu2** *mol 1*	2.2191(5)<br>2.2301(6)	2.1188(15)<br>2.0031(14)	92.325(18)	80.06(12)	89.54
mol 2	2.2033(6)<br>2.2283(5)	2.0874(15)<br>2.0076(14)	92.738(17)	81.34(6)	89.96
**Cu3** *mol 1*	2.2562(18)<br>2.2446(18)	2.115(6)<br>2.029(5)	89.08(6)	80.7(2)	87.90
mol 2	2.2498(19)<br>2.2388(17)	2.144(6)<br>2.014(6)	90.09(6)	79.0(2)	85.53
**Cu4**	2.2373(9)<br>2.2093(10)	1.989(3)<br>2.111(3)	91.76(3)	80.06(12)	89.41
**Cu5**	2.1920(6)<br>2.2474(6)	1.996(2)<br>2.094(2)	91.66(2).	81.22(8)	88.58
**Cu6**	2.2982(10)<br>2.2432(8)	2.116(3)<br>2.016(3)	80.35(10)	90.70(3)	88.83
**Cu8**	2.2186(4)<br>2.2297(4)	2.0037(13)<br>2.0998(14)	91.551(16)	81.26(5)	82.38
**Cu9**[b]	2.231(6)<br>2.164(7)	2.109(14)<br>1.974(14)	91.4(3)	79.7(6)	89.74
**Cu11**	2.268(2)<br>2.273(2)	2.072(7)<br>2.085(6)	80.2(2)	96.83(7)	87.29

[a]*Angle between the planes defined by the two phosphorus atoms and the copper center and the plane formed by the two nitrogen atoms and the copper cente.* [b]*The data were not deposited as explained in the text.*

References

1 Alexander, R. P.; Schroeder, H. Chemistry of Decaborane-Phosphorus Compounds. IV. Monomeric, Oligomeric, and CyclicPhosphinocarboranes. *Inorg. Chem.* **1963**, 2, 1107−1110.

2 Teixidor, F.; Viñas, C.; Abad, M. M.; Nuñez, R.; Kivekäs, R.; Sillanpäa, R. Procedure for the degradation of 1,2-$(PR_2)_2$ -1,2-dicarba-*closo*-dodecaborane(12) and 1-(PR_2)-2-R′-1,2-dicarba-*closo*-dodecaborane(12). *J. Organomet. Chem.* **1995**, *503*, 193−203.

3 Bourakadi, K. E.; Mekhzoum, M. E. M.; Saby, C.; Morjani, H.; Chakchak, H.; Merghoub, N.; Qaiss, A. E. K.; Bouhfid, R. Synthesis, characterization and *in vitro* anticancer activity of thiabendazole-derived 1,2,3-triazole derivatives. *New J. Chem.* **2020**, *44* , 12099–12106.

4 Zhang, C.; Zhong, B.; Yang, S.; Pan, L.; Yu, S.; Li, Z.; Li, B. Su, S.; Meng, X. Synthesis and biological evaluation of thiabendazole derivatives as anti-angiogenesis and vascular disrupting agents. Bioorg. *Med. Chem.* **2015**, *23*, 3774–3780.

5 Redrado, M.; Miñana, M.; Coogan, M. P.; Gimeno, M. C.; Fernández-Moreira, V. Tunable Emissive Ir(III) Benzimidazole-quinoline Hybrids as Promising Theranostic Lead Compounds, *ChemMedChem* **2022**, *17*, e202200244.

6 Yang, D.; Fokas, D.; Li, J.; Yu, L.; Baldino, C. M. Versatile Method for the Synthesis of Benzimidazoles from o-Nitroanilines and Aldehydes in One Step via a Reductive Cyclization, *Synthesis* **2005**, *1*, 47−56.

7 Shan, G.-G.; Li, H.-B.; Sun, H.-Z.; Cao, H.-T.; Zhu, D.-X.; Su, Z.-M. Enhancing the luminescence properties and stability of cationic iridium(III) complexes based on phenylbenzoimidazole ligand: a combined experimental and theoretical study. *Dalton Trans.* **2013**,*42*,11056–11065.
